# Achieving Diverse Functionalizations of Electron-Deficient Aryl Halides via Carbonate-Mediated Electron-Donor–Acceptor Complexes

**DOI:** 10.1002/ejoc.70519

**Published:** 2026-05-16

**Authors:** Krishnakumar Sachidanandan, Cole Stenftenagel, Athul Joshy, Sébastien Laulhé

**Affiliations:** Department of Chemistry & Chemical Biology, Indiana University Indianapolis, Indianapolis, Indiana, USA

**Keywords:** arylation, aryl halide functionalization, borylation, carbonate anion, chalcogenation, dehalogenation, EDA complex

## Abstract

Aryl halides are excellent precursors for functional group interconversion due to their malleability and diversity. They are also among the most common building blocks due to their use in cross-coupling reactions. Traditional methodologies for activating these motifs make use of expensive precious metals, such as palladium, and often require the use of complex and even more expensive ligands. Electron-donor–acceptor complexes have recently gained attention as a new photo-induced approach for the activation of various molecular motifs, including aryl halides. Herein, further extension of these methodologies led to the development of a straightforward and simple protocol that uses inexpensive carbonate bases for the activation of electron-deficient aryl iodides and bromides. The procedure was employed for the arylation, dehalogenation, deuteration, chalcogenation, and borylation of aryl halide starting materials.

## 1 | Introduction

Aryl halides are quintessential building blocks in organic chemistry. Similarly, aryl halides are ideal handles for subsequent functional group interconversions [1–6]. The most common approaches to achieve these transformations make use of late-transition metals such as palladium [4, 7–11]. With the advent of photoredox reactions and electrochemical approaches, the single electron reduction of aryl halides has gained significant attention in the past few years as a straightforward approach for the transformation of these diverse and valuable starting materials [12–15]. Indeed, upon reduction, aryl halides generate the corresponding aromatic radicals that serve as the reactive species for further functionalization. Recently, electron-donor–acceptor (EDA) complexes have been used for the generation of radicals from various starting materials [16–25], including aryl halides [26–33]. While traditional photo-reduction of aryl halides requires the use of catalysts [9, 34–36], many of which can be expensive [37], an EDA strategy proceeds through the simple use of either an inexpensive sacrificial electron-donor, or electron-donor coupling partners [28, 30].

Recently, the Pericàs group demonstrated, in an elegant amidation of indoles, that carbonate anions generate EDA complexes with highly electron-deficient *N*-aryloxyamides [38]. Similarly, the Watson group reported the use of K_2_CO_3_ as the single-electron transfer agent for the thermal activation of electron-deficient alkylpyridinium salts to generate sulfinimines [39]. Lastly, an EDA complex between the carbonate anion and electron-deficient aryl halides was reported by the Roy group for borylation and phosphorylation [40].

**SCHEME 1 |:**
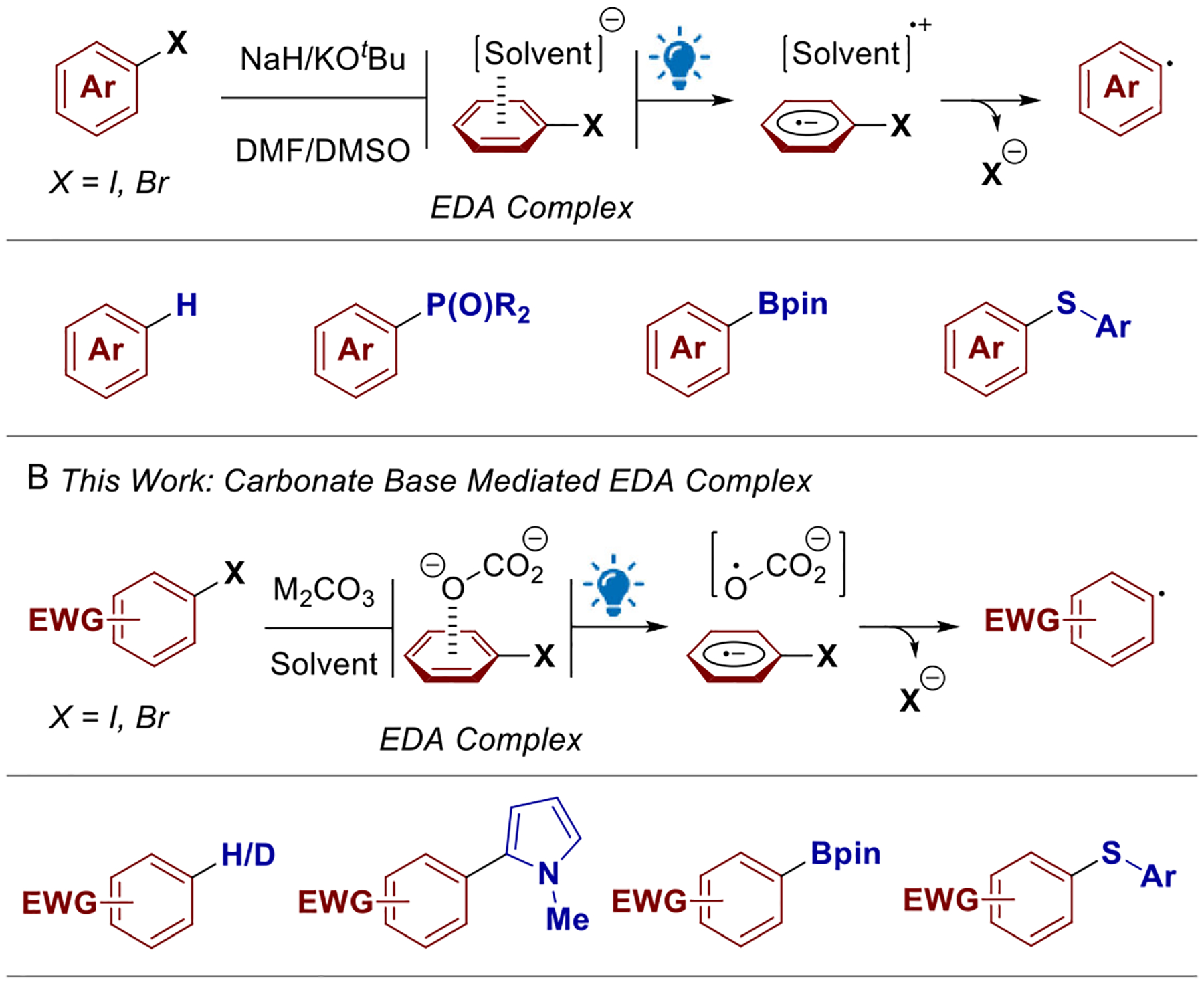
(A) Our previous reports on EDA complex and (B) this work.

In previous work, our group demonstrated that solvent anions of DMF and DMSO behaved as strong electron-donors in EDA complexes with aryl halides achieving borylations, phosphonations, and chalcogenations (Scheme 1A) [26, 27]. As we continued to explore base-mediated transformations of aryl halides, we discovered that electron-deficient aryl iodides underwent facile dehalogenation using a carbonate base under visible light irradiation. Leveraging this observation, herein we further expanded the scope of transformations into dehalogenation, arylation, chalcogenation, and borylation (Scheme 1B).

## 2 | Results and Discussion

Utilizing 4-iodoacetophenone (**1a**) as the model substrate, Li_2_CO_3_ as base and methanol as solvent, we obtained dehalogenated product **1** in 81% yield (Table 1, entry 1). Changing the solvent to dichloromethane drastically reduced the yield to 22% (Entry 2). Changing the counter ion to cesium also reduced the product formation to 65%. Additionally, altering the light source to 427 nm reduced the yield to 17% and no reaction was observed in dark (entry 4 and 5, respectively). Finally, a decrease in yield was observed when the reaction was done under air (entry 6).

Further expanding the facile formation of electron-deficient aryl radicals using carbonate bases and inspired by the ability of *N*-methyl pyrrole to trap aryl radical [41], we explored the arylation of these motifs.

Using 4-iodoacetophenone (**1a**) and *N*-methyl pyrrole (**7a**) as the model substrates, Cs_2_CO_3_ as base, and acetonitrile as the solvent, we obtained the desired cross-coupled product **7** in 70% yield (Table 2, Entry 1). Switching solvent to methanol reduced the yield to 52% (Entry 2). Changing the counter ion to lithium also reduced the yield to 53% (Entry 3). Neither increasing nor reducing the loading of *N*-methyl pyrrole improved the yield (Entry 4 and 5, respectively). Comparable trends were observed when base loadings were altered, with yields of 66% and 60% for 2 equiv. and 4 equiv. of Cs_2_CO_3_, respectively. Changing the lamp to 390 nm reduced the product formation to 64% and the reaction was not generating any desired product in the absence of light (Entry 8 and 9, respectively). Finally, the effect of atmosphere was explored by performing the reaction in air, which drastically dropped the yield to 24% [42].

**SCHEME 2 |:**
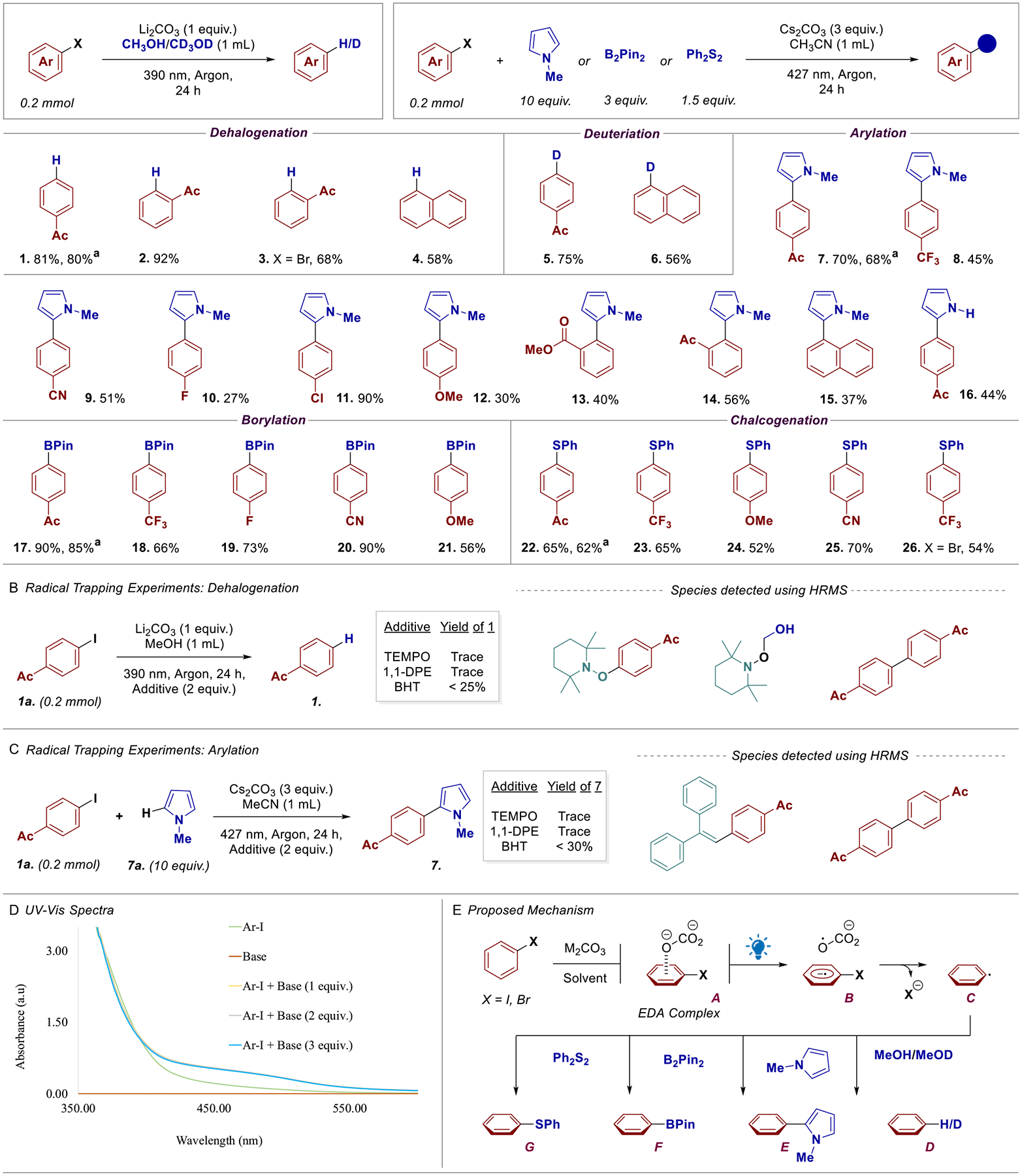
(A) Substrate scope, ^a^1.0 mmol; (B) and (C) radical trapping studies; (D) UV–Vis studies; (E) proposed mechanism.

With the optimized conditions in hand, we explored the scope of this reaction using various aryl halides (Scheme 2A). Dehalohydrogenated products **1** and **2** were formed in excellent yields of 81% and 92%, respectively. Larger scale (1 mmol) reaction of **1** was also performed, providing the product in virtually identical (80%) yield. 2-Bromoacetophenone also afforded the desired product **3** in 68% yield. 1-Iodonapthalene was dehalogenated to form naphthalene **4** within 58% yield. The same starting materials were employed for dehalodeuteriation by replacing CH_3_OH with CD_3_OD, and both provided the desired products **5** and **6** in good yields (75% and 56%, respectively).

Next, we turned our attention toward the arylation of *N*-methyl pyrrole, starting with 4-iodoacetophenone, generating product **7** in good 70% yield, and 68% yield for 1 mmol scale reaction. Other electron withdrawing scaffolds, including 4-iodobenzotrifluride, 4-iodobenzonitrile, and 4-iodoflurobenzene provided the desired products **8**, **9**, and **10** in 45%, 51%, and 27% yield, respectively. Interestingly, 4-iodochlorobenzene afforded the desired arylated product **11** with 90% yield while preserving the chlorine moiety for subsequent transformations.

As expected, electron-donating motifs, such as 4-iodoanisole, afforded product **12** in moderate 30% yield due to their inability to form efficient EDA complexes with weak electron-donor carbonate anions. This result emphasizes the reactivity-selectivity principle applied to EDA complexes when compared to our previous base-mediated activations of aryl halides [26, 27], which use strong KO^*t*^Bu and NaH bases to generate highly reducing solvent anion species that can activate both electron-rich and electron-deficient aryl halides. Ortho-substituted scaffolds generated the desired biaryl systems, **13** and **14**, in 40% and 56% yield, respectively. Electron-neutral 1-iodonaphthalene also afforded product **15** in 37% yield, presumably due to its weakerinteraction as an electron-acceptor. Finally, pyrrole motif was able to generate the desired product **16** with 4-iodoacetophenone in 44% yield. Using the same optimized conditions as for the arylation reaction, we explored the thiolation and the borylation of electron-deficient aryl halides (**17** to **26**) (Scheme 2A). Additionally, 1 mmol scale reactions for substrates **17** and **22** afforded the desired products in comparably high yields. As expected, these transformations were also successful generating the desired products in good yields when compared to previous reports [26–33, 40].

Next, we turned our attention to understanding the underlying mechanism for these transformations, starting with radical trapping experiments (Scheme 2B,C). Adding TEMPO, 1,1-diphenylethylene (1,1-DPE), and butylated hydroxytoluene (BHT) to the reaction mixtures led to a significant reduction of the desired product formation while simultaneously trapping the radical intermediates, including the aryl radical dimers, in both dehalogenation and arylation reactions [43].

Then, we performed UV–Vis experiments to substantiate the possibility of an EDA complex (Scheme 2D) [44]. As shown, the addition of Cs_2_CO_3_ to a solution of electron-deficient aryl halide in CH_3_CN led to a substantial increase in absorbance when compared to aryl halide alone. These results strongly suggest the formation of a photoactive aggregate between the carbonate anion and the aryl halide [45, 46]. Importantly, incremental addition of base to 2 equiv. and 3 equiv. did not lead to an increase in absorbance.

With the collected experimental results and literature precedents, we propose that the electron-deficient aryl halide and the carbonate anion form an EDA complex **A** (Scheme 2E). Photo-excitation of **A** leads to a single-electron reduction of the aryl halides by the carbonate to form radical anion **B** and carbonate radical. Loss of the halogen anion generates the reactive aryl radical intermediate **C**, which undergoes a hydrogen-atom transfer with methanol or methanol-*D*^4^ to generate the desired dehalohydrogenation/deuteriation product **D** [47]. Similarly, aryl radical **C** can perform a radical addition to, (i) *N*-methyl pyrrole, to generate the arylated product **E** [10]; (ii) bis(pinacolato)diboron to form the borylated arenes **F** [26]; and (iii) diphenyl disulfide to provide the chalcogenated product **G** (Scheme 2E) [27].

## 3 | Conclusion

In summary, we have developed a mild and cost-effective carbonate-mediated EDA system for the transformation of aryl halides. This protocol facilitates broad functionalizations, including dehalogenation and arylation, as well as the installation of heteroatoms such as chalcogens and boron. Furthermore, successful deuterium labeling was shown, providing a streamlined route to isotope labeling. By utilizing a simple inorganic base to drive radical generation, this work underscores the growing utility of EDA complexes as robust, accessible tools for modern synthetic chemistry.

## 4 | Experimental Section

### 4.1 | General Procedure for Dehalogenation

Aryl iodide (0.2 mmol, 1.0 equiv), Li_2_CO_3_ (14.8 mg, 0.2 mmol, 1 equiv.) and methanol (1.0 mL) were combined in a 10 mL microwave vial equipped with a magnetic stir bar under an argon atmosphere. The vial was sealed with a septum cap and positioned approximately 3 cm from two 390 nm blue LEDs (40 W). The reaction temperature was maintained at approximately 35°C due to irradiation-induced heating. After stirring for 24 h, the reaction mixture was quenched with water (10 mL) and extracted with DCM (3 × 10 mL). The combined organic layers were dried over anhydrous Na_2_SO_4_, filtered, and concentrated under reduced pressure. The residue was further purified by flash column, eluting with ethyl acetate:hexane to obtain the desired dehalogenated product.

### 4.2 | General Procedure for Arylation

Aryl iodide (0.2 mmol, 1.0 equiv), Cs_2_CO_3_ (195.5 mg, 0.6 mmol, 3.0 equiv), *N*-methyl pyrrole (177.5 μL, 2.0 mmol, 10.0 equiv) and acetonitrile (1.0 mL) were combined in a 10 mL microwave vial equipped with a magnetic stir bar under an argon atmosphere. The vial was sealed with a septum cap and positioned approximately 3 cm from two 390 nm blue LEDs (40 W). The reaction temperature was maintained at approximately 35°C due to irradiation-induced heating. After stirring for 24 h, the reaction mixture was quenched with water (10 mL) and extracted with DCM (3 × 10 mL). The combined organic layers were dried over anhydrous Na_2_SO_4_, filtered, and concentrated under reduced pressure. The residue was further purified by flash column, eluting with ethyl acetate:hexane to obtain the desired arylated product.

## Supplementary Material

Supporting Information

## Figures and Tables

**TABLE 1 |:** Optimization of reaction conditions for dehalogenation.

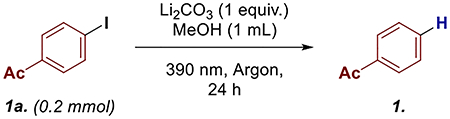		
Entry	Variation from standard condition	Yield of 1, %
1.	—	81
2.	DCM instead of MeOH	22
3.	Cs_2_CO_3_ instead of Li_2_CO_3_	65
4.	427 nm instead of 390 nm	17
5.	Dark instead of 390 nm	N/R
6.	Air instead of Argon	43

^1^H NMR yields using 1,2-dibromoethane as internal standard.

**TABLE 2 |:** Optimization of reaction conditions for arylation.

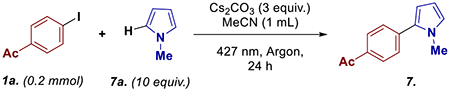		
Entry	Variation from standard condition	Yield of 7, %
1.	—	70
2.	MeOH instead of MeCN	52
3.	Li_2_CO_3_ instead of Cs_2_CO_3_	53
4.	5 equiv. of N-methyl pyrrole	34
5.	20 equiv. of N-methyl pyrrole	49
6.	2 equiv. of Cs_2_CO_3_	66
7.	4 equiv. of Cs_2_CO_3_	60
8.	390 nm instead of 427 nm	64
9.	Dark instead of 427 nm	N/R
10.	Air instead of Argon	24

^1^H NMR yields using 1,2-dibromoethane as internal standard.

## Data Availability

The data that support the findings of this study are available in the supplementary material of this article.
