# Supplementary material for: Achieving Diverse Functionalizations of Electron-Deficient Aryl Halides via Carbonate-Mediated Electron-Donor–Acceptor Complexes
Source: European J Org Chem. Author manuscript; Available in PMC 2026 May 30. (PMC13221195; doi:10.1002/ejoc.70519)
Supplement: Supporting Information [file NIHMS2178474-supplement-Supporting_Information.pdf]

Supporting Information

Achieving Diverse Functionalizations of Electron-Deficient Aryl Halides via Carbonate-Mediated Electron-Donor-Acceptor Complexes

Krishnakumar Sachidanandan, Cole Stenftenagel, Athul Joshy, Sébastien Laulhé*

Indiana University Indianapolis,

Indianapolis, Indiana 46202, United States.

Corresponding Author Email: slaulhe@iu.edu

Table of Contents

1. General Information SI-3
2. General Procedures SI-4
3. Optimization Study SI-6
4. Experimental Details and Characterization Data for Products SI-8
5. UV-Vis Study SI-34
6. Radical Trapping Study SI-38
7. ^{1}H NMR, ^{13}C NMR, ^{19}F NMR Spectra SI-42
8. References SI-74

1. General Information

All reagents and solvents were purchased and used without further purification unless otherwise noted. All reactions were performed under an inert atmosphere unless otherwise stated. Room temperature refers to 26 °C, unless otherwise noted. Moisture-sensitive reactions were performed using flame-dried glassware under an atmosphere of dry argon (Ar). Air- and water sensitive reactions were setup in a Vacuum Atmosphere GENESIS glovebox held under an atmosphere of argon gas (working pressure 2−6 mbar). Flame-dried equipment was stored in a 130 °C oven before use and either allowed to cool in a cabinet desiccator or assembled hot and allowed to cool under an inert atmosphere. Chromatographic purification of products were performed manually, using silica flash column chromatography (Fisher Chemical™ Silica Gel Sorbent 230-400 Mesh, Grade 60) or automatically, using Teledyne Isco Combi*Flash RF*+ UV Flash Chromatography System with RediSep Silver flash columns. Thin-layer chromatography was performed on EMD Millipore silica gel 60 F254 glass-backed plates (layer thickness 250 µm, particle size 10−12 µm, impregnated with a fluorescent indicator). Visualization of the developed chromatogram was accomplished by fluorescence quenching under shortwave UV light and/or by staining with phosphomolybdic acid, p-anisaldehyde, or $KMnO_4$ stains.

LED Lamps. The following Kessil LED lamps were used in this work:
- 390 nm lamp: PR160L-390, 40W (purple visible light)
- 427 nm lamp: PR160L-427, 40W (blue visible light)
- 440 nm lamp: PR160L-440, 40W (blue visible light)
- 525 nm lamp: PR160L-525, 40W (green visible light)

Reaction Vials. We used ChemGlass microwave reaction vials with heavy walls made of borosilicate glass (product # CG-4920-01). The vial was placed approximately 3 cm away from the LED lamps, with the LEDs shining directly at the side of the vial as shown in following picture. Three reactions per lamp could be set up at the same time. And a fan above the reaction vials can keep the temperature around 35 °C. 10 mL microwave reaction vial secured by 20 mm aluminum seals with 0.125-inch thick, blue PTFE / white silicone septa was used for the reaction.

Instrumentation. For NMR spectrometry, NMR spectra were obtained on Bruker spectrometers operating at 400 or 500 MHz for ^{1}H NMR and 101 or 126 MHz for ^{13}C{1 H} NMR. The data were reported in the following order: chemical shifts (δ ppm), multiplicity (s = singlet, d = doublet, dd = doublet of doublets, t = triplet, q = quartet, m = multiplet), coupling constant, (Hz), relative integral made in reference to NMR solvent signals. For mass spectrometry, gas chromatograph−mass spectrometry was obtained using a Agilent GC System Intuvo 9000 Series coupled with a Agilent 5977B GC/MSD Mass Selective Detector. High resolution mass spectra were obtained using a Thermo Orbitrap Fusion Tribid Mass Spectrometer with electrospray ionization (ESI).

2. General Procedures

2.1 General procedure for dehalogenation.

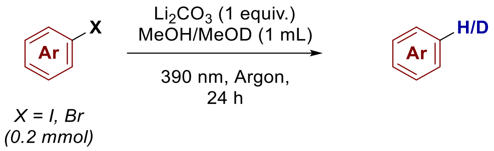

Aryl iodide derivative (0.2 mmol, 1.0 equiv), Li_2CO_3 (14.8 mg, 0.2 mmol, 1 equiv.) and methanol/methanol-D_4 (1.0 mL) were combined in a 10 mL microwave vial equipped with a magnetic stir bar under an argon atmosphere. The vial was sealed with a septum cap and positioned approximately 3 cm from two 390 nm blue LEDs (40 W). The reaction temperature was maintained at approximately 35 °C due to irradiation-induced heating. After stirring for 24 h, the reaction mixture was quenched with water (10 mL) and extracted with DCM (3 × 10 mL). The combined organic layers were dried over anhydrous Na_2SO_4, filtered, and concentrated under reduced pressure. The residue was further purified by flash column chromatography on silica gel.

2.2 General procedure for arylation, borylation and chalcogenation.

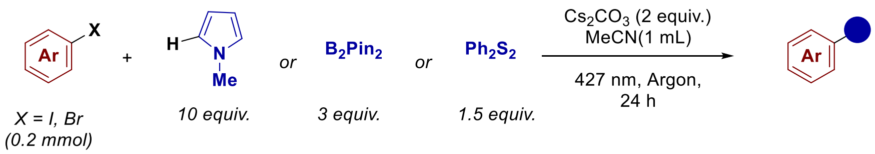

Aryl iodide derivative (0.2 mmol, 1.0 equiv), Cs_2CO_3 (195.5 mg, 0.6 mmol, 3.0 equiv), *N*-methyl pyrole (177.5 μL, 2.0 mmol, 10.0 equiv) or B_2Pin_2 (150.0 mg, 0.6 mmol, 3.0 equiv) or Ph_2S_2 (65.5 mg, 0.3 mmol, 1.5 equiv), and acetonitrile (1.0 mL) were combined in a 10 mL microwave vial equipped with a magnetic stir bar under an argon atmosphere. The vial was sealed with a septum cap and positioned approximately 3 cm from two 390 nm blue LEDs (40 W). The reaction temperature was maintained at approximately 35 °C due to irradiation-induced heating. After stirring for 24 h, the reaction mixture was quenched with water (10 mL) and extracted with DCM (3 × 10 mL). The combined organic layers were dried over anhydrous Na_2SO_4, filtered, and concentrated under reduced pressure. The residue was further purified by flash column chromatography on silica gel.

2.3 General procedure for dehalogenation (1.0 mmol).

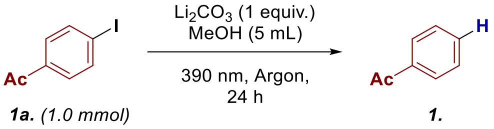

Aryl iodide derivative, 4-iodoacetophenone (**1a**) (246.0 mg, 1.0 mmol, 1.0 equiv), Li_2CO_3 (74.0 mg, 1.0 mmol, 1 equiv.) and methanol (5.0 mL) were combined in a 10 mL microwave vial equipped with a magnetic stir bar under an argon atmosphere. The vial was sealed with a septum cap and positioned approximately 3 cm from two 390 nm blue LEDs (40 W). The reaction temperature was maintained at approximately 35 °C due to irradiation-induced heating. After stirring for 24 h, the reaction mixture was quenched with water (30 mL) and extracted with DCM (3 × 30 mL). The combined organic layers were dried over anhydrous Na_2SO_4, filtered, and concentrated under reduced pressure. The residue was further purified by flash column chromatography on silica gel.

2.4 General procedure for arylation, borylation and chalcogenation (1.0 mmol).

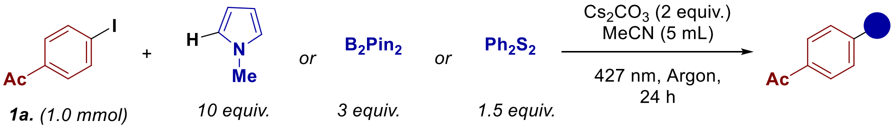

Aryl iodide derivative 4-iodoacetophenone (**1a**) (246.0 mg, 1.0 mmol, 1.0 equiv), Cs_2CO_3 (975.0 mg, 3.0 mmol, 3.0 equiv), *N*-methyl pyrole (890.0 μL, 10.0 mmol, 10.0 equiv) or B_2Pin_2 (750.0 mg, 3.0 mmol, 3.0 equiv) or Ph_2S_2 (330.0 mg, 1.5 mmol, 1.5 equiv), and acetonitrile (5.0 mL) were combined in a 10 mL microwave vial equipped with a magnetic stir bar under an argon atmosphere. The vial was sealed with a septum cap and positioned approximately 3 cm from two 390 nm blue LEDs (40 W). The reaction temperature was maintained at approximately 35 °C due to irradiation-induced heating. After stirring for 24 h, the reaction mixture was quenched with water (30 mL) and extracted with DCM (3 × 30 mL). The combined organic layers were dried over anhydrous Na_2SO_4, filtered, and concentrated under reduced pressure. The residue was further purified by flash column chromatography on silica gel.

3. Optimization Study:

3.1 Optimization for dehalogenation

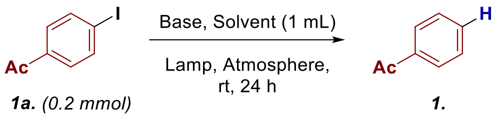

Table S1. ^{1}HNMR yields using 1,2-dibromoethane as internal standard.

Entry	Solvent	Base	Lamp	Atmosphere	Time	Yield (1)
1	MeOH	Cs_2CO_3 (2 equiv)	390 nm	Argon	24 h	49%
2	MeOH	K_2CO_3 (2 equiv)	390 nm	Argon	24 h	56%
3	MeOH	Na_2CO_3 (2 equiv)	390 nm	Argon	24 h	68%
4	MeOH	K_3PO_4 (2 equiv)	390 nm	Argon	24 h	46%
5	MeOH	Li_2CO_3 (2 equiv)	390 nm	Argon	24 h	74%
6	CH_3Cl	Li_2CO_3 (2 equiv)	390 nm	Argon	24 h	49%
7	H_2O	Li_2CO_3 (2 equiv)	390 nm	Argon	24 h	N/R
8	DCM	Li_2CO_3 (2 equiv)	390 nm	Argon	24 h	22%
9	EtOH	Li_2CO_3 (2 equiv)	390 nm	Argon	24 h	52%
10	CH_3CN	Li_2CO_3 (2 equiv)	390 nm	Argon	24 h	9%
11	Acetone	Li_2CO_3 (2 equiv)	390 nm	Argon	24 h	13%
14	MeOH	Li_2CO_3 (2 equiv)	427 nm	Argon	24 h	17%
15	MeOH	Li_2CO_3 (2 equiv)	440 nm	Argon	24 h	11%
16	MeOH	Li_2CO_3 (2 equiv)	456 nm	Argon	24 h	Trace
17	MeOH	Li_2CO_3 (2 equiv)	Dark	Argon	24 h	N/R
16	MeOH	Li_2CO_3 (1 equiv)	390 nm	Argon	24 h	81%
17	MeOH	Li_2CO_3 (2 equiv)	390 nm	Argon	24 h	74%
18	MeOH	Li_2CO_3 (3 equiv)	390 nm	Argon	24 h	44%
20	MeOH	Li_2CO_3 (1 equiv)	390 nm	Nitrogen	24 h	60%
21	MeOH	Li_2CO_3 (1 equiv)	390 nm	Air	24 h	43%

3.2 Optimization for arylation

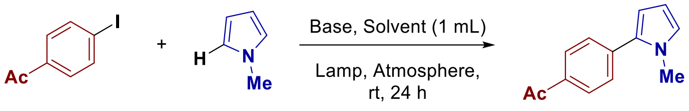

1a. *(0.2 mmol)* *N*-Methyl Pyrrole *(X equiv.)* **7.**

Table S2. ^{1}HNMR yields using 1,2-dibromoethane as internal standard.

Entry	Solvent	Base	Methyl Pyrrole	Lamp	Atmosphere	Time	Yield (7)
1	Acetone	Li_2CO_3 (3 equiv)	10 equiv	390 nm	Argon	24 h	42%
2	MeOH	Li_2CO_3 (3 equiv)	10 equiv	390 nm	Argon	24 h	27%
3	DCM	Li_2CO_3 (3 equiv)	10 equiv	390 nm	Argon	24 h	13%
4	DMF	Li_2CO_3 (3 equiv)	10 equiv	390 nm	Argon	24 h	41%
5	MeCN	Li_2CO_3 (3 equiv)	10 equiv	390 nm	Argon	24 h	53%
6	MeCN	Cs_2CO_3 (3 equiv)	10 equiv	390 nm	Argon	24 h	64%
7	MeCN	K_2CO_3 (3 equiv)	10 equiv	390 nm	Argon	24 h	58%
8	MeCN	Na_2CO_3 (2 equiv)	10 equiv	390 nm	Argon	24 h	61%
9	MeCN	Cs_2CO_3 (3 equiv)	5 equiv	390 nm	Argon	24 h	34%
10	MeCN	Cs_2CO_3 (3 equiv)	10 equiv	390 nm	Argon	24 h	64%
11	MeCN	Cs_2CO_3 (3 equiv)	20 equiv	390 nm	Argon	24 h	49%
12	MeCN	Cs_2CO_3 (3 equiv)	30 equiv	390 nm	Argon	24 h	60%
13	MeCN	Cs_2CO_3 (3 equiv)	40 equiv	390 nm	Argon	24 h	62%
14	MeCN	Cs_2CO_3 (1 equiv)	10 equiv	390 nm	Argon	24 h	54%
15	MeCN	Cs_2CO_3 (2 equiv)	10 equiv	390 nm	Argon	24 h	66%
16	MeCN	Cs_2CO_3 (3 equiv)	10 equiv	390 nm	Argon	24 h	62%
17	MeCN	Cs_2CO_3 (4 equiv)	10 equiv	390 nm	Argon	24 h	60%
18	MeCN	Cs_2CO_3 (5 equiv)	10 equiv	390 nm	Argon	24 h	60%
19	MeCN	Cs_2CO_3 (2 equiv)	10 equiv	427 nm	Argon	24 h	70%
20	MeCN	Cs_2CO_3 (2 equiv)	10 equiv	440 nm	Argon	24 h	49%
21	MeCN	Cs_2CO_3 (2 equiv)	10 equiv	456 nm	Argon	24 h	38%
22	MeCN	Cs_2CO_3 (2 equiv)	10 equiv	Dark	Argon	24 h	N/R
23	MeCN	Cs_2CO_3 (2 equiv)	10 equiv	427 nm	Air	24 h	24%

4. Experimental Details and Characterization Data for Products:

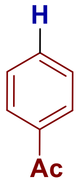

Known compound reported by reference.[1]

Acetophenone (1): Reaction using 4-iodoacetophenone (**1a**) (49.2 mg, 0.2 mmol, 1.0 equiv), under standard condition according to **General Procedure 2.1**. The residue was further purified by flash column, eluting with ethyl acetate:hexane (1:5) as yellow oil 19.4 mg, 81% yield.

^{1}H NMR (400 MHz, $CDCl_3$) δ 7.96 (d, J = 7.3 Hz, 2H), 7.56 (t, J = 7.4 Hz, 1H), 7.46 (t, J = 7.6 Hz, 2H), 2.61 (s, 3H).

^{13}C NMR (101 MHz, $CDCl_3$) δ 198.2, 137.2, 133.1, 128.6, 128.3, 26.6.

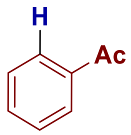

Known compound reported by reference.[1]

Acetophenone (2): Reaction using 2-iodoacetophenone (**2a**) (49.2 mg, 0.2 mmol, 1.0 equiv), under standard condition according to **General Procedure 2.1**. The residue was further purified by flash column, eluting with ethyl acetate:hexane (1:5) as yellow oil 22.1 mg, 92% yield.

^{1}H NMR (400 MHz, $CDCl_3$) δ 7.96 (d, J = 7.7 Hz, 2H), 7.55 (t, J = 7.4 Hz, 1H), 7.46 (t, J = 7.6 Hz, 2H), 2.61 (s, 3H).

^{13}C NMR (101 MHz, $CDCl_3$) δ 198.2, 137.2, 133.1, 128.6, 128.3, 26.6.

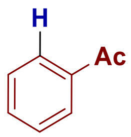

Known compound reported by reference.[1]

Acetophenone (3): Reaction using 2-bromoacetophenone (**3a**) (39.8 mg, 0.2 mmol, 1.0 equiv), under standard condition according to **General Procedure 2.1**. The residue was further purified by flash column, eluting with ethyl acetate:hexane (1:5) as yellow oil 16.3 mg, 68% yield.

^{1}H NMR (400 MHz, $CDCl_3$) δ 7.95 (d, J = 7.1 Hz, 2H), 7.55 (t, J = 7.4 Hz, 1H), 7.45 (t, J = 7.6 Hz, 3H), 2.59 (s, 5H).

^{13}C NMR (101 MHz, $CDCl_3$) δ 198.1, 137.2, 133.1, 128.6, 128.3, 26.6.

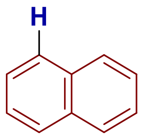

Known compound reported by reference.[1]

Napthalene (4): Reaction using 1-iodonaphthalene (**4a**) (50.8 mg, 0.2 mmol, 1.0 equiv), under standard condition according to **General Procedure 2.1**. The residue was further purified by flash column, eluting with ethyl acetate:hexane (1:99) as white solid 14.8 mg, 58% yield.

^{1}H NMR (400 MHz, $CDCl_3$) δ 7.85 (m, 4H), 7.49 (m, 4H).

^{13}C NMR (101 MHz, $CDCl_3$) δ 133.5, 127.9, 125.8.

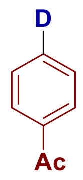

Known compound reported by reference.[1]

1-(phenyl-4-d)ethan-1-one (5): Reaction using 4-iodoaetophenone (**1a**) (49.2 mg, 0.2 mmol, 1.0 equiv), under standard condition. according to **General Procedure 2.1**. The residue was further purified by flash column, eluting with ethyl acetate:hexane (1:5) as colorless oil 18.1 mg, 75% yield.

^{1}H NMR (400 MHz, $CDCl_3$) δ 7.96 (d, J = 8.2 Hz, 2H), 7.46 (d, J = 7.8 Hz, 2H), 2.61 (s, 3H).

^{13}C NMR (101 MHz, $CDCl_3$) δ 198.2, 137.2, 133.1, 128.6, 128.5, 128.3, 26.6.

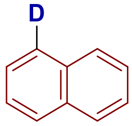

Known compound reported by reference.[1]

Naphthalene-1-d (6): Reaction using 1-Iodonapthalene (**4a**) (50.8 mg, 0.2 mmol, 1.0 equiv), under standard condition. according to **General Procedure 2.1**. The residue was further purified by flash column, eluting with ethyl acetate:hexane (1:5) as yellow oil 14.4 mg, 56% yield.

^{1}H NMR (400 MHz, $CDCl_3$) δ 7.91 – 7.77 (m, 3H), 7.55 – 7.40 (m, 4H).

^{13}C NMR (101 MHz, $CDCl_3$) δ 133.4, 127.9, 127.8, 125.8, 125.7.

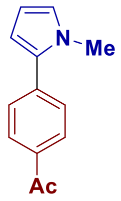

Known compound reported by reference.[2]

1-(4-(1-methyl-1H-pyrrol-2-yl)phenyl)ethan-1-one (7): Reaction using 4-iodoacetophenone (**1a**) (49.2 mg, 0.2 mmol, 1.0 equiv), under standard condition. according to **General Procedure 2.2**. The residue was further purified by flash column, eluting with ethyl acetate:hexane (1:5) as yellow oil 27.9, 70% yield.

^{1}H NMR (400 MHz, $CDCl_3$) δ 7.99 (d, J = 8.3 Hz, 2H), 7.51 (d, J = 8.4 Hz, 2H), 6.83 – 6.74 (m, 1H), 6.41 – 6.33 (m, 1H), 6.28 – 6.19 (m, 1H), 3.72 (s, 3H), 2.62 (s, 3H).

^{13}C NMR (101 MHz, $CDCl_3$) δ 197.6, 138.0, 135.0, 133.4, 128.6, 127.9, 125.4, 110.3, 108.4, 35.5, 26.6.

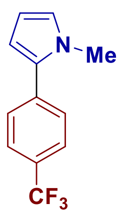

Known compound reported by reference.[3]

1-methyl-2-(4-(trifluoromethyl)phenyl)-1*H*-pyrrole (8): Reaction using 4-iodobenzotrifluride (**8a**) (29.3 μL, 0.2 mmol, 1.0 equiv), under standard condition according to **General Procedure 2.2**. The residue was further purified by flash column, eluting with ethyl acetate:hexane (1:10) as white solid 20.3 mg, 45% yield.

^{1}H NMR (400 MHz, $CDCl_3$) δ 7.65 (d, J = 8.1 Hz, 2H), 7.52 (d, J = 8.6 Hz, 2H), 6.78 – 6.75 (m, 1H), 6.34 – 6.29 (m, 1H), 6.26 – 6.21 (m, 1H), 3.70 (s, 3H).

^{13}C NMR (101 MHz, $CDCl_3$) δ 136.8, 133.1, 128.51 (q, J = 32.5 Hz), 128.4, 125.4 (q, J = 3.8 Hz), 124.9, 124.31 (q, J = 271.8 Hz), 109.9, 108.3, 35.2.

^{19}F NMR (376 MHz, $CDCl_3$) δ -62.43.

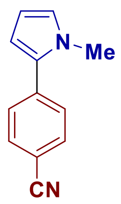

Known compound reported by reference.[3]

4-(1-methyl-1H-pyrrol-2-yl)benzonitrile (9): Reaction using 4-iodobenzonitrile (**9a**) (45.8 mg, 0.2 mmol, 1.0 equiv), under standard condition according to **General Procedure 2.2**. The residue was further purified by flash column, eluting with ethyl acetate:hexane (2:3) as light yellow solid 18.6 mg, 51% yield.

^{1}H NMR (400 MHz, $CDCl_3$) δ 7.67 (d, J = 8.4 Hz, 2H), 7.50 (d, J = 8.4 Hz, 2H), 6.82 – 6.73 (m, 1H), 6.39 – 6.31 (m, 1H), 6.27 – 6.19 (m, 1H), 3.71 (s, 3H).

^{13}C NMR (101 MHz, $CDCl_3$) δ 137.7, 132.6, 132.3, 128.3, 125.9, 119.0, 110.8, 109.8, 108.6, 35.4.

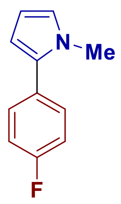

Known compound reported by reference.[3]

2-(4-fluorophenyl)-1-methyl-1*H*-pyrrole (10): Reaction using 4-iodoflurobenzene (**10a**) (22.7 μL, 0.2 mmol, 1.0 equiv), under standard condition according to **General Procedure 2.2**. The residue was further purified by flash column, eluting with ethyl acetate:hexane (1:19) as light-yellow oil 9.4 mg, 27% yield.

^{1}H NMR (400 MHz, $CDCl_3$) δ 7.40 – 7.32 (m, 2H), 7.14 – 7.05 (m, 2H), 6.73 – 6.70 (m, 1H), 6.22 – 6.18 (m, 2H), 3.64 (s, 3H).

^{13}C NMR (101 MHz, $CDCl_3$) δ 161.95 (d, J = 246.2 Hz), 133.7, 130.4 (d, J = 8.0 Hz), 129.5 (d, J = 3.3 Hz), 123.5, 115.30 (d, J = 21.4 Hz), 108.6, 107.8, 35.0.

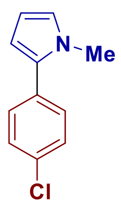

Known compound reported by reference.[3]

2-(4-chlorophenyl)-1-methyl-1*H*-pyrrole (11): Reaction using 1-Chloro-4-iodobenzene (**11a**) (47.7 mg, 0.2 mmol, 1.0 equiv), under standard condition according to **General Procedure 2.2**. The residue was further purified by flash column, eluting with ethyl acetate:hexane (1:20) as colorless oil 34.4 mg, 90% yield.

^{1}H NMR (400 MHz, $CDCl_3$) δ 7.41 – 7.31 (m, 4H), 6.79 – 6.67 (m, 1H), 6.26 – 6.15 (m, 2H), 3.66 (s, 3H).

^{13}C NMR (101 MHz, $CDCl_3$) δ 133.4, 132.7, 131.8, 129.8, 128.6, 124.1, 109.0, 107.9, 35.1.

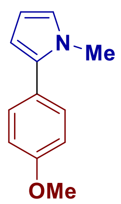

Known compound reported by reference.[3]

2-(4-methoxyphenyl)-1-methyl-1*H*-pyrrole (12): Reaction using 4-iodoanisole (**12a**) (46.8 mg, 0.2 mmol, 1.0 equiv), under standard condition according to **General Procedure 2.2**. The residue was further purified by flash column, eluting with ethyl acetate:hexane (1:5) as brown solid 11.2 mg, 30% yield.

^{1}H NMR (400 MHz, $CDCl_3$) δ 7.32 (d, J = 8.8 Hz, 2H), 6.94 (d, J = 8.8 Hz, 2H), 6.72 – 6.66 (m, 1H), 6.25 – 6.08 (m, 2H), 3.84 (s, 3H), 3.63 (s, 3H).

^{13}C NMR (101 MHz, $CDCl_3$) δ 158.7, 134.4, 130.0, 126.0, 123.0, 113.8, 108.0, 107.6, 55.3, 34.9.

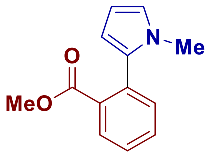

Known compound reported by reference.[4]

Methyl 2-(1-methyl-1*H*-pyrrol-2-yl)benzoate (13): Reaction using methyl 2-iodobenzoate (**13a**) (29.4 μL, 0.2 mmol, 1.0 equiv), under standard condition according to **General Procedure 2.2**. The residue was further purified by flash column, eluting with ethyl acetate:hexane (1:5) as yellow oil 17.2 mg, 40% yield.

^{1}H NMR (400 MHz, $CDCl_3$) δ 7.92 – 7.87 (m, 1H), 7.56 – 7.50 (m, 1H), 7.46 – 7.37 (m, 2H), 6.73 – 6.67 (m, 1H), 6.22 – 6.17 (m, 1H), 6.10 – 6.03 (m, 1H), 3.73 (s, 3H), 3.41 (s, 3H).

^{13}C NMR (101 MHz, $CDCl_3$) δ 168.1, 133.9, 132.7, 132.4, 131.9, 131.3, 129.8, 127.8, 122.26, 108.44, 107.5, 77.0, 52.2, 34.2.

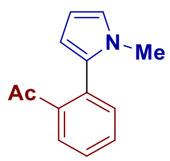

1-(2-(1-methyl-1H-pyrrol-2-yl)phenyl)ethan-1-one (14): Reaction using 2-iodoacetophenone (**2a**) (49.2 mg, 0.2 mmol, 1.0 equiv), under standard condition according to **General Procedure 2.2**. The residue was further purified by flash column, eluting with ethyl acetate:hexane (1:5) as yellow oil 22.3 mg, 56% yield.

^{1}H NMR (400 MHz, $CDCl_3$) δ 7.65 – 7.58 (m, 1H), 7.55 – 7.47 (m, 1H), 7.47 – 7.35 (m, 2H), 6.81 – 6.69 (m, 1H), 6.28 – 6.18 (m, 1H), 6.18 – 6.09 (m, 1H), 3.42 (s, 3H), 1.92 (s, 3H).

^{13}C NMR (101 MHz, $CDCl_3$) δ 203.9, 141.7, 131.9, 131.6, 131.4, 130.8, 128.0, 127.9, 123.3, 110.3, 108.4, 34.3, 28.8.

HRMS (ESI-TOF) m/z: $[M + H]^+$ calcd. for $C_{13}H_{13}NO$ 200.1075, found 200.1078

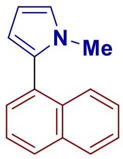

Known compound reported by reference.[5]

1-methyl-2-(naphthalen-1-yl)-1H-pyrrole (15): Reaction using 1-iodonaphthalene (**4a**) (50.8 mg, 0.2 mmol, 1.0 equiv), under standard condition according to **General Procedure 2.2**. The residue was further purified by flash column, eluting with ethyl acetate:hexane (1:5) as colorless oil 15.3 mg, 37% yield.

^{1}H NMR (400 MHz, $CDCl_3$) δ 7.94 – 7.85 (m, 2H), 7.77 – 7.69 (m, 1H), 7.56 – 7.43 (m, 4H), 6.87 – 6.79 (m, 1H), 6.38 – 6.30 (m, 1H), 6.30 – 6.20 (m, 1H), 3.40 (s, 3H).

^{13}C NMR (101 MHz, $CDCl_3$) δ 133.7, 133.4, 132.2, 131.3, 128.8, 128.2, 128.2, 126.3, 126.3, 125.9, 125.2, 122.4, 110.0, 107.6, 34.5.

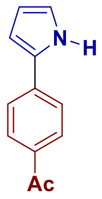

Known compound reported by reference.[5]

1-(4-(1H-pyrrol-2-yl)phenyl)ethan-1-one (16): Reaction using 4-iodoacetophenone (**1a**) (49.2 mg, 0.2 mmol, 1.0 equiv), and Pyrrole (138.0 μL, 2.0 mmol, 10.0 equiv.) under standard condition according to **General Procedure 2.2**. The residue was further purified by flash column, eluting with ethyl acetate:hexane (1:20) as yellow oil 16.3 mg, 44% yield.

^{1}H NMR (400 MHz, $CDCl_3$) δ 8.90 (s, 1H), 7.94 (d, J = 8.4 Hz, 2H), 7.55 (d, J = 8.5 Hz, 2H), 6.97 – 6.89 (m, 1H), 6.74 – 6.63 (m, 1H), 6.41 – 6.27 (m, 1H), 2.59 (s, 3H).

^{13}C NMR (101 MHz, $CDCl_3$) δ 197.7, 137.1, 134.4, 130.9, 129.3, 123.2, 120.6, 110.7, 108.2, 26.5.

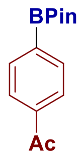

Known compound reported by reference.[6]

1-(4-(4,4,5,5-tetramethyl-1,3,2-dioxaborolan-2-yl)phenyl)ethan-1-one (17): Reaction using 4-iodoacetophenone (**1a**) (49.2 mg, 0.20 mmol, 1.0 equiv), under standard condition according to **General Procedure 2.2**. The residue was further purified by flash column, eluting with ethyl acetate:hexane (1:5) as colorless oil 44.3 mg, 90% yield.

^{1}H NMR (400 MHz, $CDCl_3$) δ 7.97 – 7.84 (m, 4H), 2.62 (s, 3H), 1.36 (s, 12H).

^{13}C NMR (101 MHz, $CDCl_3$) δ 198.5, 139.0, 134.9, 127.3, 84.2, 83.5, 26.8, 24. 9.

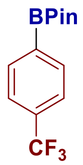

Known compound reported by reference.[6]

4,4,5,5-tetramethyl-2-(4-(trifluoromethyl)phenyl)-1,3,2-dioxaborolane (18): Reaction using 4-iodobenzotrifluride (**8a**) (29.3 μL, 0.2 mmol, 1.0 equiv), under standard condition according to **General Procedure 2.2**. The residue was further purified by flash column, eluting with ethyl acetate:hexane (1:5) as colorless oil 35.9 mg, 66% yield.

^{1}H NMR (400 MHz, $CDCl_3$) δ 7.92 (d, J = 7.7 Hz, 2H), 7.61 (d, J = 7.8 Hz, 2H), 1.36 (s, 12H).

^{13}C NMR (101 MHz, $CDCl_3$) δ 135.0, 132.8 (q, J = 32.1 Hz), 124.3 (q, J = 3.9 Hz), 124.1 (d, J = 272.4 Hz), 84.3, 24.9.

^{19}F NMR (376 MHz, $CDCl_3$) δ -63.05.

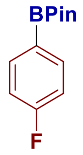

Known compound reported by reference.[7]

2-(4-fluorophenyl)-4,4,5,5-tetramethyl-1,3,2-dioxaborolane (19): Reaction using 4-iodoflurobenzene (**10a**) (22.7 μL, 0.2 mmol, 1.0 equiv), under standard condition according to **General Procedure 2.2**. The residue was further purified by flash column, eluting with ethyl acetate:hexane (1:5) as colorless oil 32.4 mg, 73% yield.

^{1}H NMR (400 MHz, $CDCl_3$) δ 7.85 – 7.76 (m, 2H), 7.10 – 7.01 (m, 2H), 1.34 (s, 12H).

^{13}C NMR (101 MHz, $CDCl_3$) δ 165.1 (d, J = 250.2 Hz), 136.9 (d, J = 8.3 Hz), 114.8 (d, J = 20.2 Hz), 83.9, 24.9.

^{19}F NMR (376 MHz, $CDCl_3$) δ -108.46.

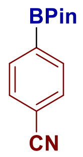

Known compound reported by reference.[7]

4-(4,4,5,5-tetramethyl-1,3,2-dioxaborolan-2-yl)benzonitrile (20): Reaction using 4-iodobenzonitrile (**9a**) (45.8 mg, 0.2 mmol, 1.0 equiv), under standard condition according to **General Procedure 2.2**. The residue was further purified by flash column, eluting with ethyl acetate:hexane (1:5) as yellow oil 41.2 mg, 90% yield.

^{1}H NMR (400 MHz, $CDCl_3$) δ 7.88 (d, J = 8.1 Hz, 2H), 7.63 (d, J = 8.2 Hz, 2H), 1.35 (s, 12H).

^{13}C NMR (101 MHz, $CDCl_3$) δ 135.1, 131.1, 118.9, 114.6, 84.5, 24.9.

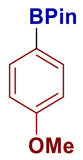

Known compound reported by reference.[7]

2-(4-methoxyphenyl)-4,4,5,5-tetramethyl-1,3,2-dioxaborolane (21): Reaction using 4-iodoanisole (**12a**) (46.8 mg, 0.2 mmol, 1.0 equiv), under standard condition according to **General Procedure 2.2**. The residue was further purified by flash column, eluting with ethyl acetate:hexane (1:15) as clear oil 26.2 mg, 56% yield.

^{1}H NMR (400 MHz, $CDCl_3$) δ 7.76 (d, J = 8.6 Hz, 2H), 6.90 (d, J = 8.6 Hz, 2H), 3.83 (s, 3H), 1.33 (s, 12H).

^{13}C NMR (101 MHz, $CDCl_3$) δ 162.2, 136.5, 113.3, 83.6, 55.1, 24.9.

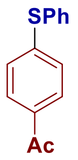

Known compound reported by reference.[8]

4-Methoxyphenyl) (phenyl)sulfane (22): Reaction using 4-iodoacetophenone (**1a**) (49.2 mg, 0.2 mmol, 1.0 equiv), under standard condition according to **General Procedure 2.2**. The residue was further purified by flash column, eluting with ethyl acetate:hexane (1:5) as colorless oil 29.6 mg, 65% yield.

^{1}H NMR (400 MHz, $CDCl_3$) δ 7.82 (d, *J* = 8.5 Hz, 2H), 7.52 – 7.45 (m, 2H), 7.45 – 7.36 (m, 3H), 7.21 (d, *J* = 8.6 Hz, 2H), 2.55 (s, 3H).

^{13}C NMR (101 MHz, $CDCl_3$) *δ* 197.1, 144.9, 134.5, 133.9, 132.1, 129.7, 128.9, 128.8, 127.5, 26.5.

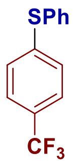

Known compound reported by reference.[7]

Phenyl(4-(trifluoromethyl)phenyl)sulfane (23): Reaction using 4-iodobenzotrifluride (**8a**) (29.3 μL, 0.2 mmol, 1.0 equiv), under standard condition according to **General Procedure 2.2**. The residue was further purified by flash column, eluting with ethyl acetate:hexane (1:5) as colorless oil 33.0 mg, 65% yield.

^{1}H NMR (400 MHz, $CDCl_3$) δ 7.52 – 7.45 (m, 4H), 7.43 – 7.36 (m, 3H), 7.30 – 7.25 (m, 2H).

^{13}C NMR (101 MHz, $CDCl_3$) δ 142.9, 133.6, 132.6, 129.7, 128.7, 128.4, 128.12 (d, J = 32.8 Hz), 125.9 (q, J = 3.9 Hz), 124.1 (q, J = 271.8 Hz).

^{19}F NMR (376 MHz, $CDCl_3$) δ -62.49.

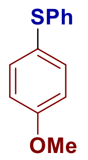

Known compound reported by reference.[7]

(4-methoxyphenyl)(phenyl)sulfane (24): Reaction using 4-iodoanisole (**12a**) (46.8 mg, 0.2 mmol, 1.0 equiv), under standard condition according to **General Procedure 2.2**. The residue was further purified by flash column, eluting with ethyl acetate:hexane (1:10) as colorless oil 22.5 mg, 52% yield.

^{1}H NMR (400 MHz, $CDCl_3$) δ 7.50 – 7.43 (m, 2H), 7.32 – 7.26 (m, 2H), 7.25 – 7.16 (m, 3H), 6.98 – 6.91 (m, 2H), 3.87 (s, 3H).

^{13}C NMR (101 MHz, $CDCl_3$) δ 159.9, 138.6, 135.4, 128.9, 128.3, 125.8, 124.4, 115.0, 55.4.

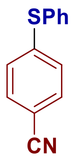

Known compound reported by reference.[9]

4-(Phenylthio)benzonitrile (25): Reaction using 4-iodobenzonitrile (**9a**) (45.8 mg, 0.2 mmol, 1.0 equiv), under standard condition according to **General Procedure 2.2**. The residue was further purified by flash column, eluting with ethyl acetate:hexane as a colorless oil 25.3 mg, 60% yield.

^{1}H NMR (400 MHz, $CDCl_3$) δ 7.54 – 7.50 (m, 2H), 7.50 – 7.46 (m, 2H), 7.46 – 7.41 (m, 3H), 7.19 – 7.15 (m, 2H).

^{13}C NMR (101 MHz, $CDCl_3$) δ 145.7, 134.5, 132.4, 130. 9, 129.9, 129.4, 127.4, 118.8, 108.8.

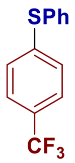

Known compound reported by reference.[7]

Phenyl(4-(trifluoromethyl)phenyl)sulfane (26): Reaction using 4-bromobenzotrifluride (**8a**) (28.1 μL, 0.2 mmol, 1.0 equiv), under standard condition according to **General Procedure 2.2**. The residue was further purified by flash column, eluting with ethyl acetate:hexane (1:5) as colorless oil 25.4 mg, 50% yield.

^{1}H NMR (400 MHz, $CDCl_3$) δ 7.51 – 7.45 (m, 4H), 7.43 – 7.36 (m, 3H), 7.29 – 7.24 (m, 2H).

^{13}C NMR (101 MHz, $CDCl_3$) δ 142.9, 133.6, 132.6, 129.7, 128.7, 128.4, 128.12 (d, J = 32.8 Hz)125.9 (q, J = 3.9 Hz), 124.15 (q, J = 271.8 Hz).

^{19}F NMR (376 MHz, $CDCl_3$) δ -62.49.

5. UV-Vis Studies:

5.1. Dehalogenation:

Preparation of Stock Solutions:

1. 4-iodoacetophenone (**Ar-I**): 0.2 mmol/mL in Acetonitrile.
2. Cs_2CO_3 (**Base** (**1 equiv.**)): 0.2 mmol/mL in Acetonitrile.
3. Cs_2CO_3 (**Base** (**2 equiv.**)): 0.4 mmol/mL in Acetonitrile.
4. Cs_2CO_3 (**Base** (**3 equiv.**)): 0.6 mmol/mL in Acetonitrile.

UV-Vis Spectra Experiments:

- Final concentrations: 0.1 mmol/mL of 4-iodoacetophenone, and 0.1/0.2/0.3 mmol/mL of Cs_2CO_3.

Experiments:

- Experiment A: 1 mL of stock 1, diluted to 2 mL in Acetonitrile.
- Experiment B: 1 mL of stock 2, diluted to 2 mL in Acetonitrile.
- Experiment C: 1 mL of stock 1 and 1 mL of stock 2.
- Experiment D: 1 mL of stock 1 and 1 mL of stock 3.
- Experiment E: 1 mL of stock 1 and 1 mL of stock 4.

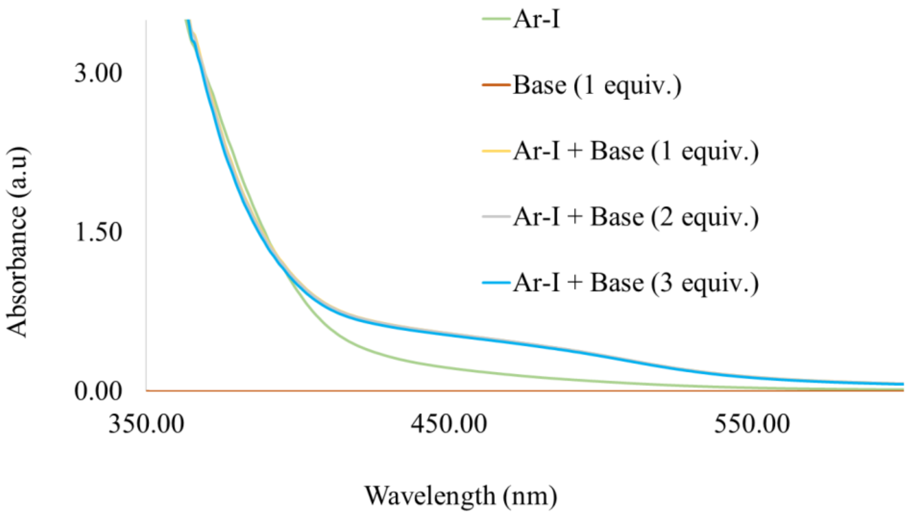

These results strongly suggest the formation of an EDA complex between the carbonate anion and the aryl halide. Importantly, incremental addition of base to 2 and 3 equivalents did not lead to an increase in absorbance.

5.2. Arylation:

Preparation of Stock Solutions:

1. 4-iodoacetophenone (**Ar-I**): 0.2 mmol/mL in Acetonitrile.
2. *N*-Methyl pyrrole (**Pyrrole**): 0.2 mmol/mL in Acetonitrile.
3. Cs_2CO_3 (**Base**): 0.2 mmol/mL in Acetonitrile.
4. **Ar-I** + **Base**: 0.2 mmol/mL of **Ar-I** & in 0.2 mmol/mL of **Base** in Acetonitrile.

UV-Vis Spectra Experiments:

- Final concentrations: 0.1 mmol/mL of 4-iodoacetophenone, 0.1 mmol/mL of *N*-Methyl pyrrole, and 0.1 mmol/mL of Cs_2CO_3.

Experiments:

- Experiment A: 1 mL of stock 1, diluted to 2 mL in Acetonitrile.
- Experiment B: 1 mL of stock 2, diluted to 2 mL in Acetonitrile.
- Experiment C: 1 mL of stock 3 diluted to 2 mL in Acetonitrile.
- Experiment D: 1 mL of stock 4 diluted to 2 mL in Acetonitrile.
- Experiment E: 1 mL of stock 2 and 1 mL of stock 3.
- Experiment F: 1 mL of stock 2 and 1 mL of stock 4.

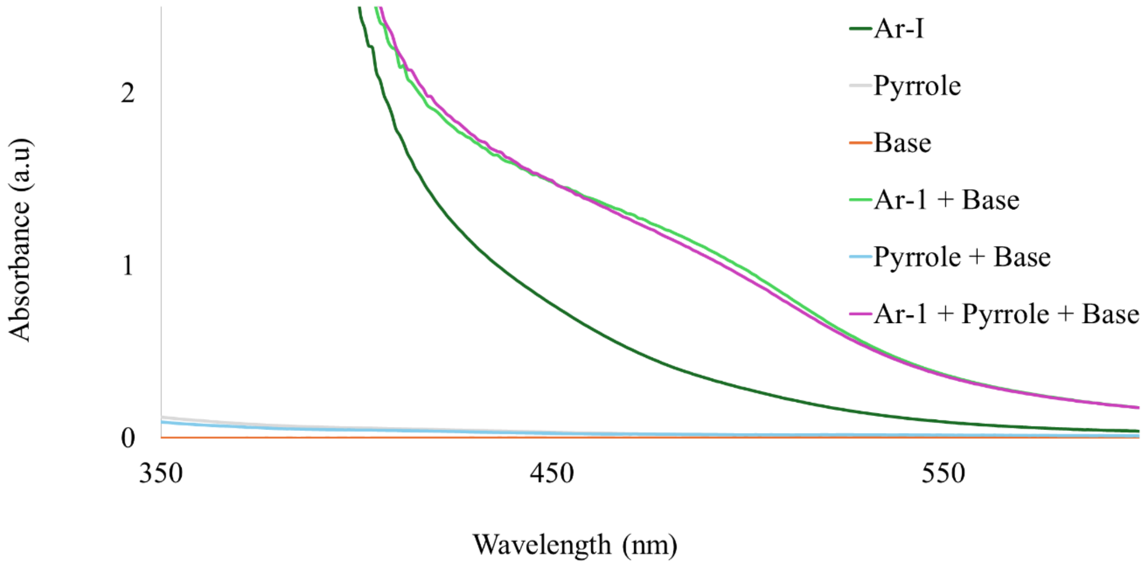

5.3. Borylation:

Preparation of Stock Solutions:

1. 4-iodoacetophenone (**Ar-I**): 0.2 mmol/mL in Acetonitrile.
2. B_2pin_2 (**B_2pin_2**): 0.2 mmol/mL in Acetonitrile.
3. Cs_2CO_3 (**Base**): 0.2 mmol/mL in Acetonitrile.
4. **Ar-I** + **Base**: 0.2 mmol/mL of **Ar-I** & in 0.2 mmol/mL of **Base** in Acetonitrile.

UV-Vis Spectra Experiments:

- Final concentrations: 0.1 mmol/mL of 4-iodoacetophenone, 0.1 mmol/mL of B_2pin_2, and 0.1 mmol/mL of Cs_2CO_3.

Experiments:

- Experiment A: 1 mL of stock 1, diluted to 2 mL in Acetonitrile.
- Experiment B: 1 mL of stock 2, diluted to 2 mL in Acetonitrile.
- Experiment C: 1 mL of stock 3 diluted to 2 mL in Acetonitrile.
- Experiment D: 1 mL of stock 4 diluted to 2 mL in Acetonitrile.
- Experiment E: 1 mL of stock 2 and 1 mL of stock 3.
- Experiment F: 1 mL of stock 2 and 1 mL of stock 4.

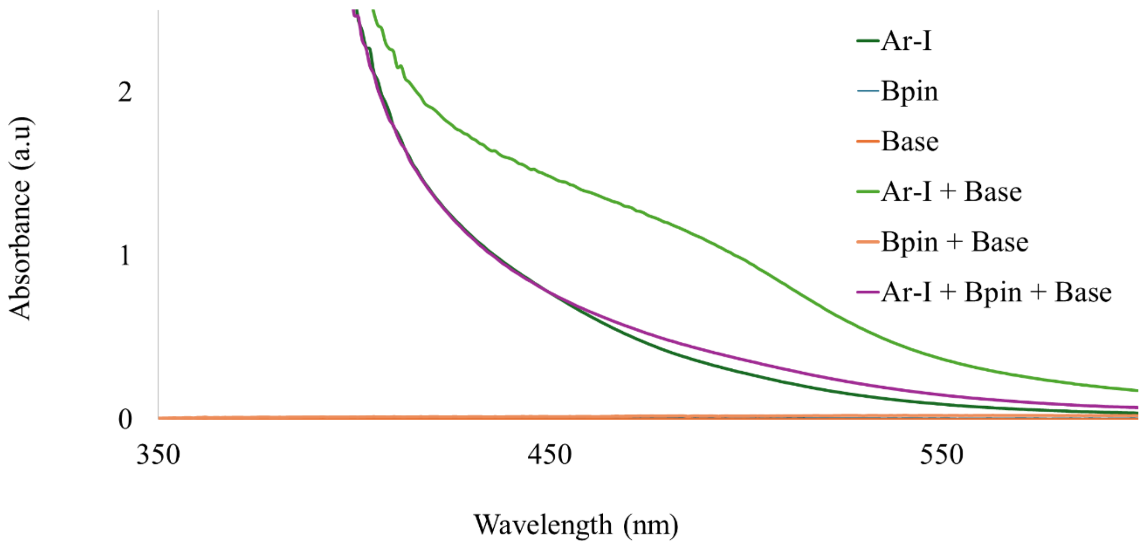

5.4. Chalcogenation:

Preparation of Stock Solutions:

5. 4-iodoacetophenone (**Ar-I**): 0.2 mmol/mL in Acetonitrile.
6. Ph_2S_2 (**Ph_2S_2**): 0.2 mmol/mL in Acetonitrile.
7. Cs_2CO_3 (**Base**): 0.2 mmol/mL in Acetonitrile.
8. **Ar-I** + **Base**: 0.2 mmol/mL of **Ar-I** & in 0.2 mmol/mL of **Base** in Acetonitrile.

UV-Vis Spectra Experiments:

- Final concentrations: 0.1 mmol/mL of 4-iodoacetophenone, 0.1 mmol/mL of Ph_2S_2, and 0.1 mmol/mL of Cs_2CO_3.

Experiments:

- Experiment A: 1 mL of stock 1, diluted to 2 mL in Acetonitrile.
- Experiment B: 1 mL of stock 2, diluted to 2 mL in Acetonitrile.
- Experiment C: 1 mL of stock 3 diluted to 2 mL in Acetonitrile.
- Experiment D: 1 mL of stock 4 diluted to 2 mL in Acetonitrile.
- Experiment E: 1 mL of stock 2 and 1 mL of stock 3.
- Experiment F: 1 mL of stock 2 and 1 mL of stock 4.

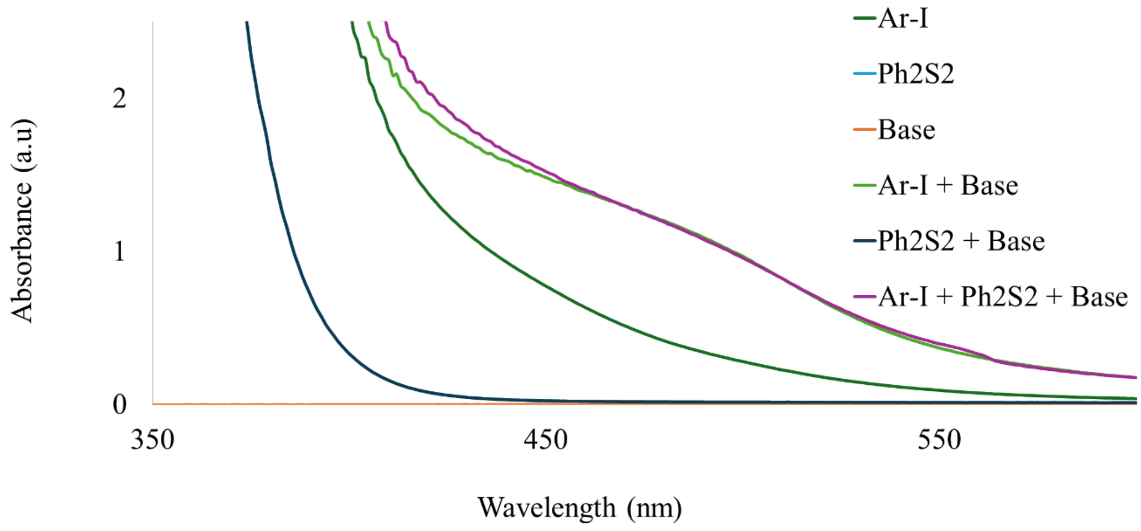

6. Radical Trapping Studies:

6.1. Dehalogenation:

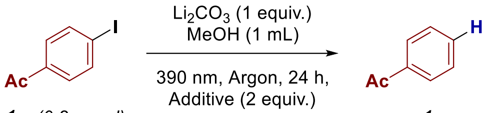

1a. *(0.2 mmol)* **1.**

Additive	Yield of 1
TEMPO	Trace
1,1-DPE	Trace
BHT	< 25%

4-iodoacetophenone (**1a**) (49.2 mg, 0.2 mmol, 1.0 equiv), Li_2CO_3 (14.8 mg, 0.2 mmol, 1 equiv.), methanol/methanol-D^4 (1.0 mL) and additive (2 equiv.) were combined in a 10 mL microwave vial equipped with a magnetic stir bar under an argon atmosphere. The vial was sealed with a septum cap and positioned approximately 3 cm from two 390 nm blue LEDs (40 W). The reaction temperature was maintained at approximately 35 °C due to irradiation-induced heating. After stirring for 24 h, the reaction mixture was poured into water (20 mL) and extracted with DCM (3 × 20 mL). The combined organic layers were dried over anhydrous Na_2SO_4, filtered, and concentrated under reduced pressure. Aliquots were collected for HRMS analysis and the crude yields were obtained using ^{1}H NMR analysis with dibromomethane as internal standard.

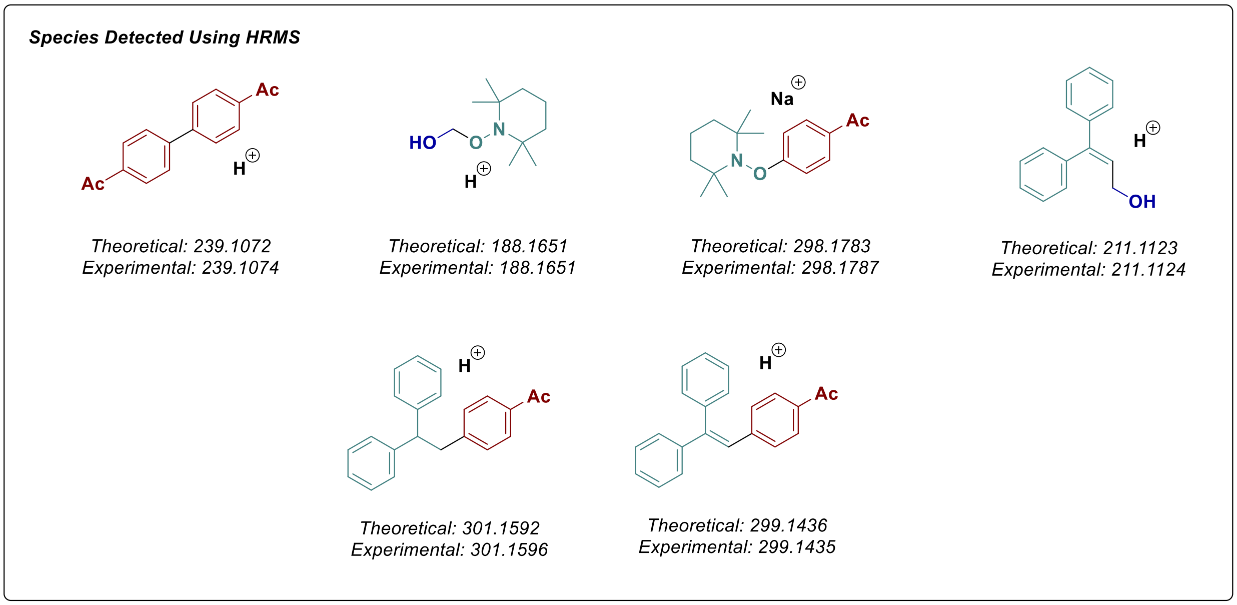

6.2. Arylation:

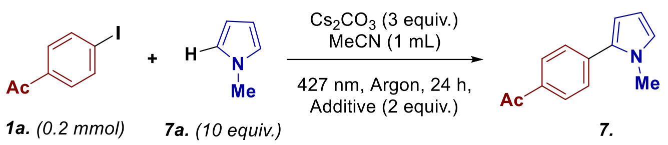

Additive	Yield of 7
TEMPO	Trace
1,1-DPE	Trace
BHT	< 30%

4-iodoacetophenone (**1a**) (49.2 mg, 0.2 mmol, 1.0 equiv), Cs_2CO_3 (195.5 mg, 0.6 mmol, 3.0 equiv), *N*-methyl pyrrole (177.5 µL, 2.0 mmol, 10.0 equiv), acetonitrile (1.0 mL), and additive (2 equiv.) were combined in a 10 mL microwave vial equipped with a magnetic stir bar under an argon atmosphere. The vial was sealed with a septum cap and positioned approximately 3 cm from two 390 nm blue LEDs (40 W). The reaction temperature was maintained at approximately 35 °C due to irradiation-induced heating. After stirring for 24 h, the reaction mixture was poured into water (20 mL) and extracted with DCM (3 × 20 mL). The combined organic layers were dried over anhydrous Na_2SO_4, filtered, and concentrated under reduced pressure. Aliquots were collected for HRMS analysis and the crude yields were obtained using ^{1}H NMR analysis with dibromomethane as internal standard.

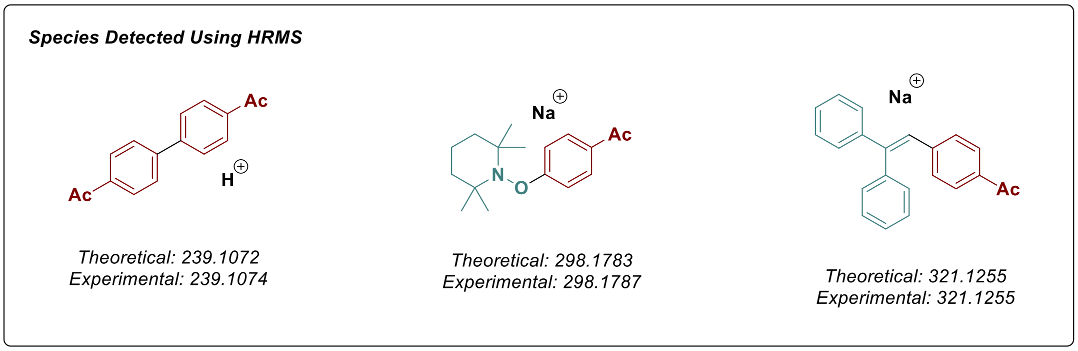

6.3. Borylation:

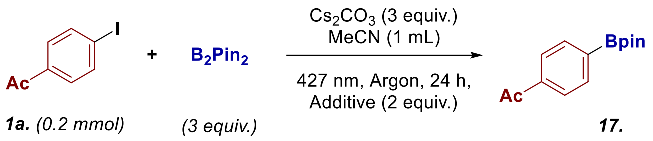

Additive	Yield of 17
TEMPO	Trace
1,1-DPE	Trace
BHT	<10%

4-iodoacetophenone (**1a**) (49.2 mg, 0.2 mmol, 1.0 equiv), Cs_2CO_3 (195.5 mg, 0.6 mmol, 3.0 equiv), B_2Pin_2 (150.0 mg, 0.6 mmol, 3.0 equiv), acetonitrile (1.0 mL), and additive (2 equiv.) were combined in a 10 mL microwave vial equipped with a magnetic stir bar under an argon atmosphere. The vial was sealed with a septum cap and positioned approximately 3 cm from two 390 nm blue LEDs (40 W). The reaction temperature was maintained at approximately 35 °C due to irradiation-induced heating. After stirring for 24 h, the reaction mixture was poured into water (20 mL) and extracted with DCM (3 × 20 mL). The combined organic layers were dried over anhydrous Na_2SO_4, filtered, and concentrated under reduced pressure. Aliquots were collected for HRMS analysis and the crude yields were obtained using ^{1}H NMR analysis with dibromomethane as internal standard.

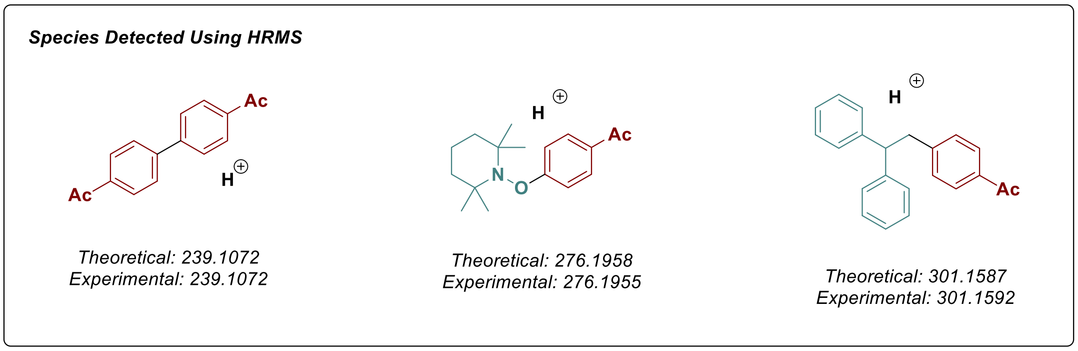

6.3. Chalcogenation:

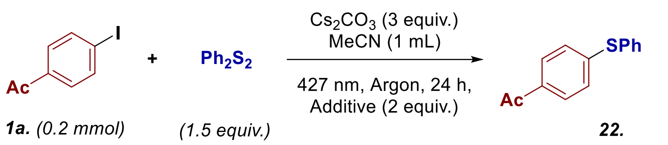

Additive	Yield of 22
TEMPO	Trace
1,1-DPE	Trace
BHT	< 20%

4-iodoacetophenone (**1a**) (49.2 mg, 0.2 mmol, 1.0 equiv), Cs_2CO_3 (195.5 mg, 0.6 mmol, 3.0 equiv), Ph_2S_2 (65.5 mg, 0.3 mmol, 1.5 equiv), acetonitrile (1.0 mL), and additive (2 equiv.) were combined in a 10 mL microwave vial equipped with a magnetic stir bar under an argon atmosphere. The vial was sealed with a septum cap and positioned approximately 3 cm from two 390 nm blue LEDs (40 W). The reaction temperature was maintained at approximately 35 °C due to irradiation-induced heating. After stirring for 24 h, the reaction mixture was poured into water (20 mL) and extracted with DCM (3 × 20 mL). The combined organic layers were dried over anhydrous Na_2SO_4, filtered, and concentrated under reduced pressure. Aliquots were collected for HRMS analysis and the crude yields were obtained using ^1H NMR analysis with dibromomethane as internal standard.

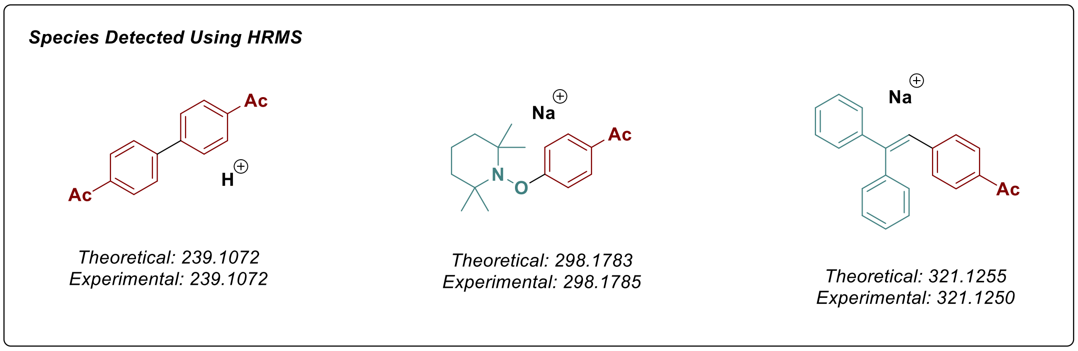

7. ^{1}H NMR, ^{13}C NMR, ^{19}F NMR, Spectra

[1]**H NMR** (400 MHz, $CDCl_3$)

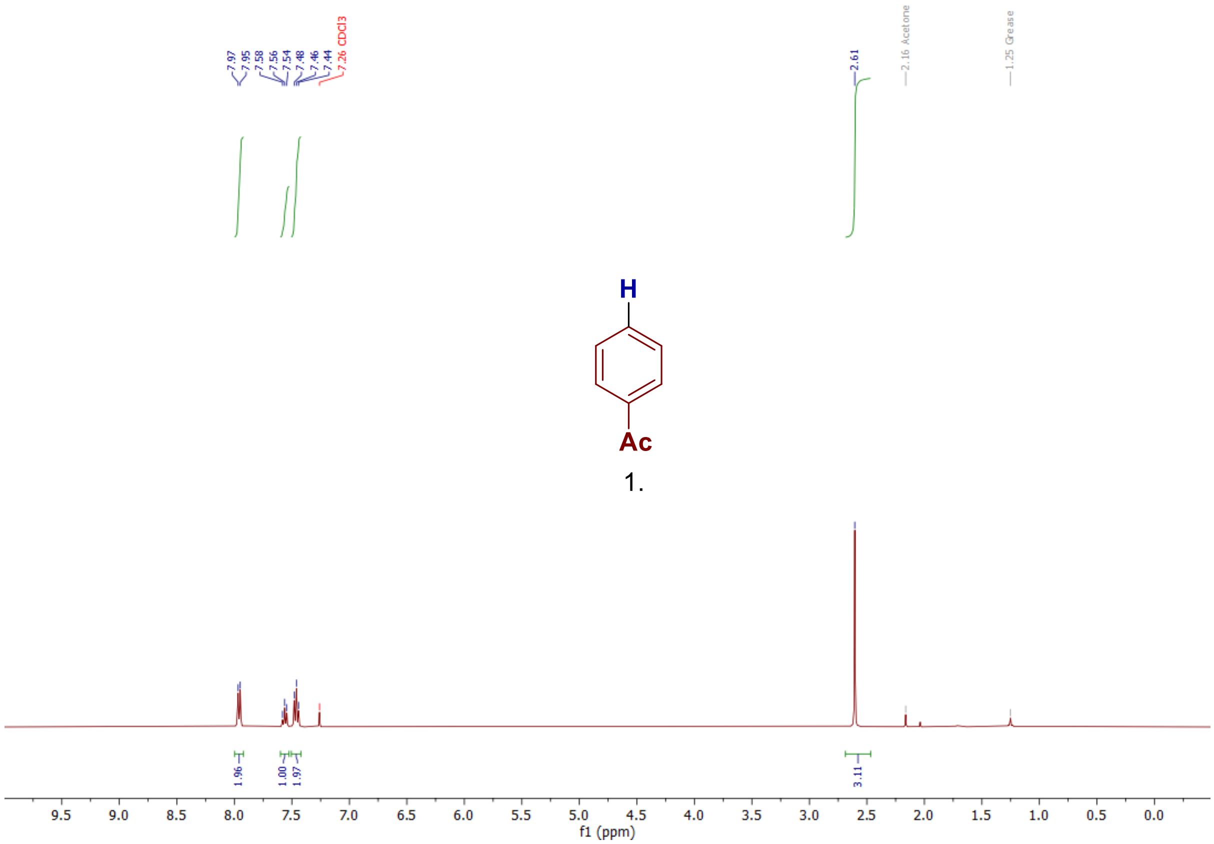

[13]**C NMR** (101 MHz, $CDCl_3$)

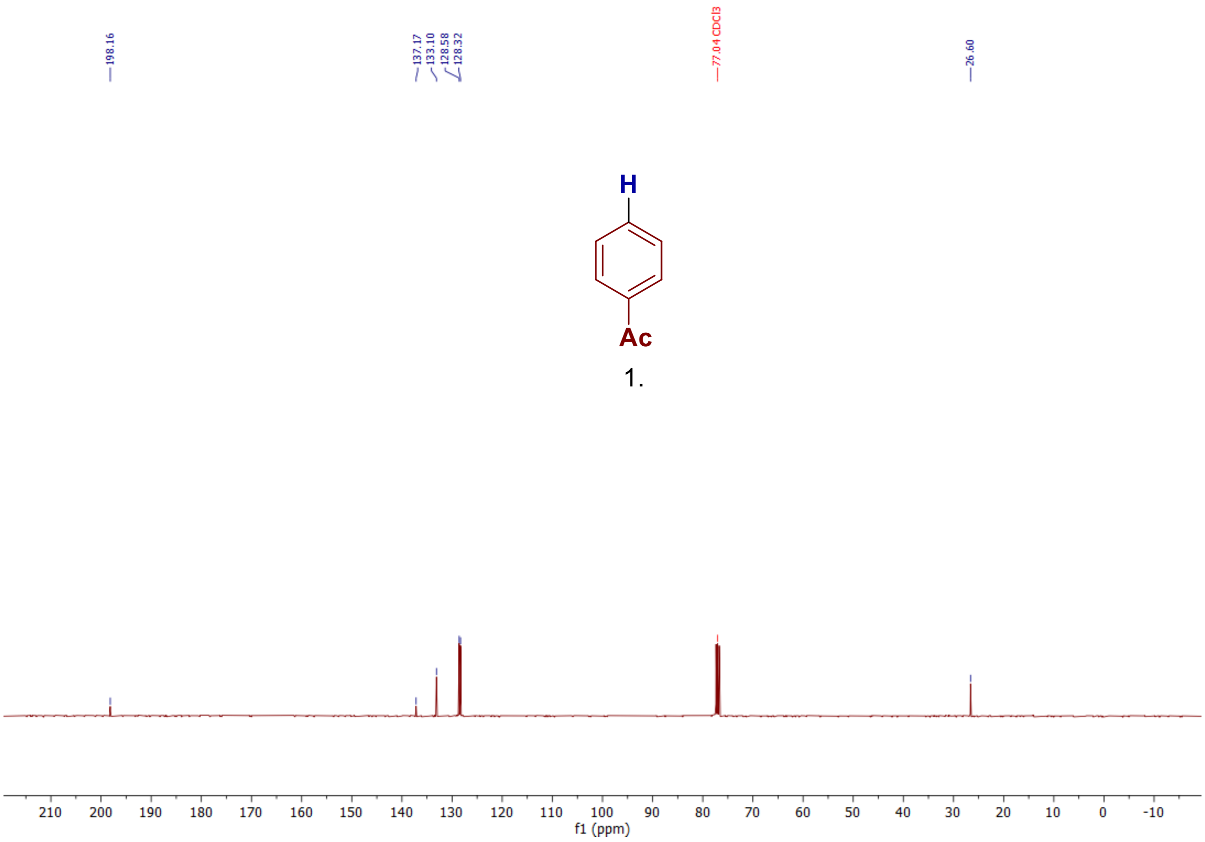

^{1}H NMR (400 MHz, $CDCl_3$)

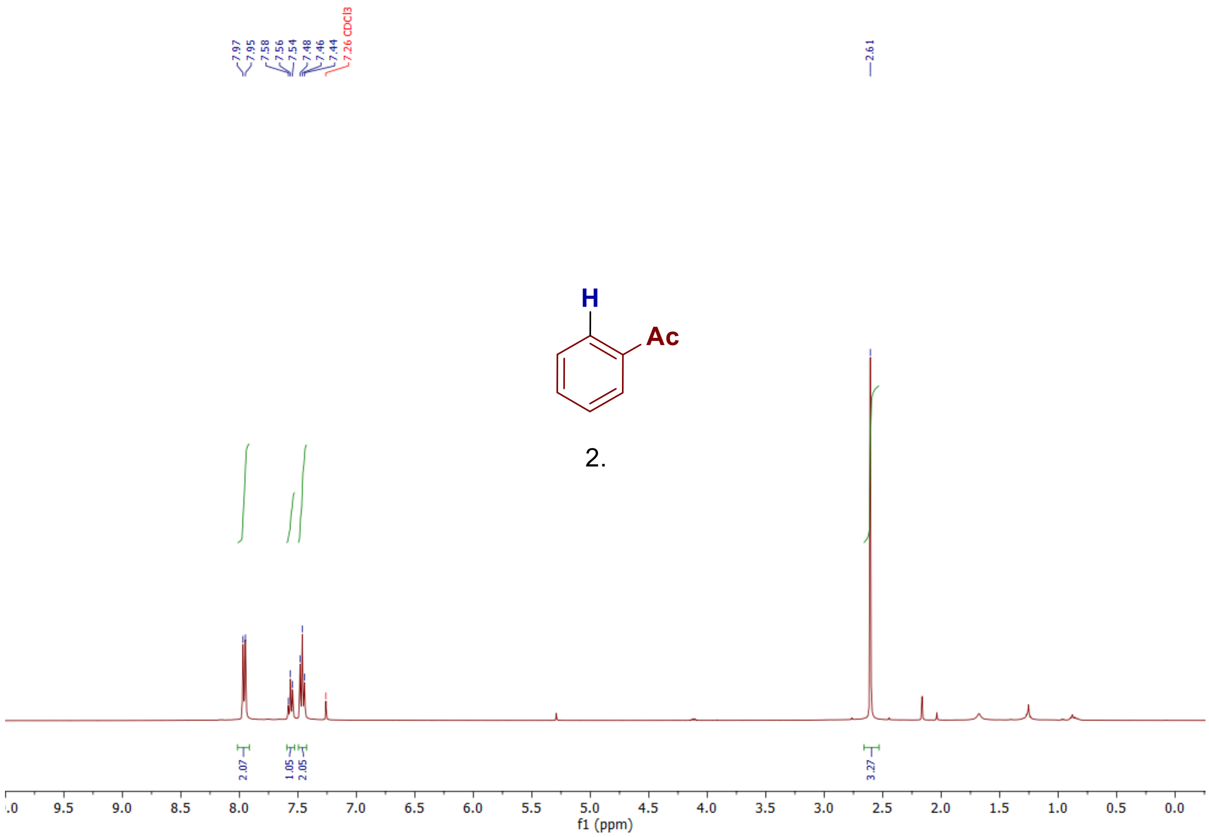

^{13}C NMR (101 MHz, $CDCl_3$)

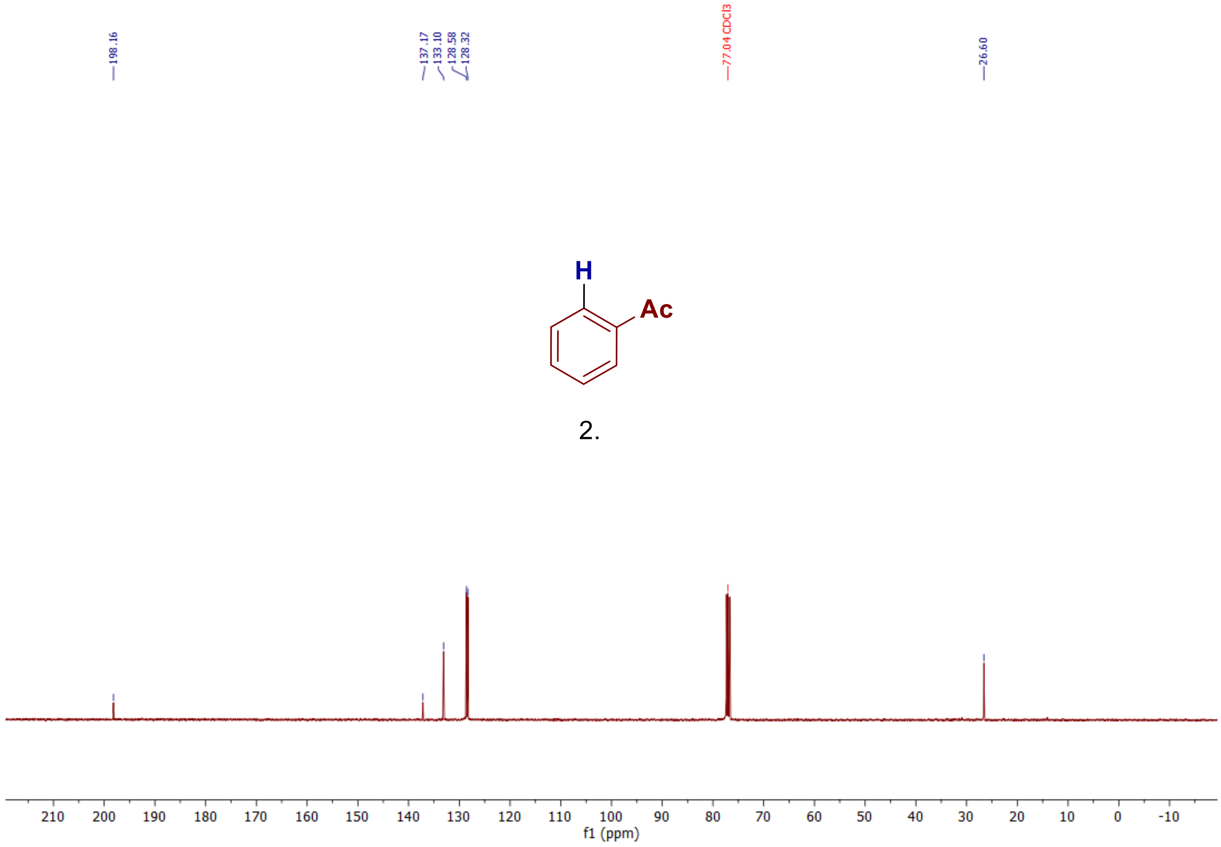

^{1}H NMR (400 MHz, $CDCl_3$)

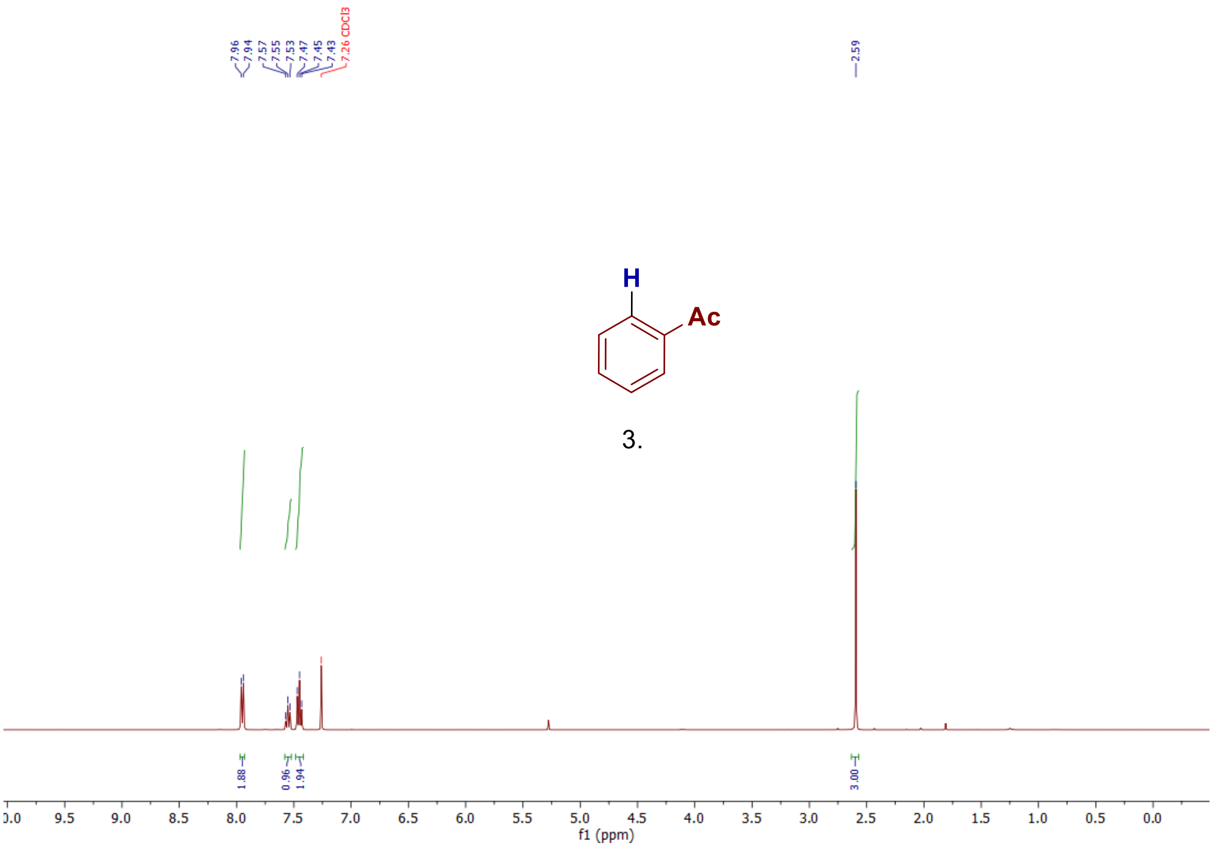

^{13}C NMR (101 MHz, $CDCl_3$)

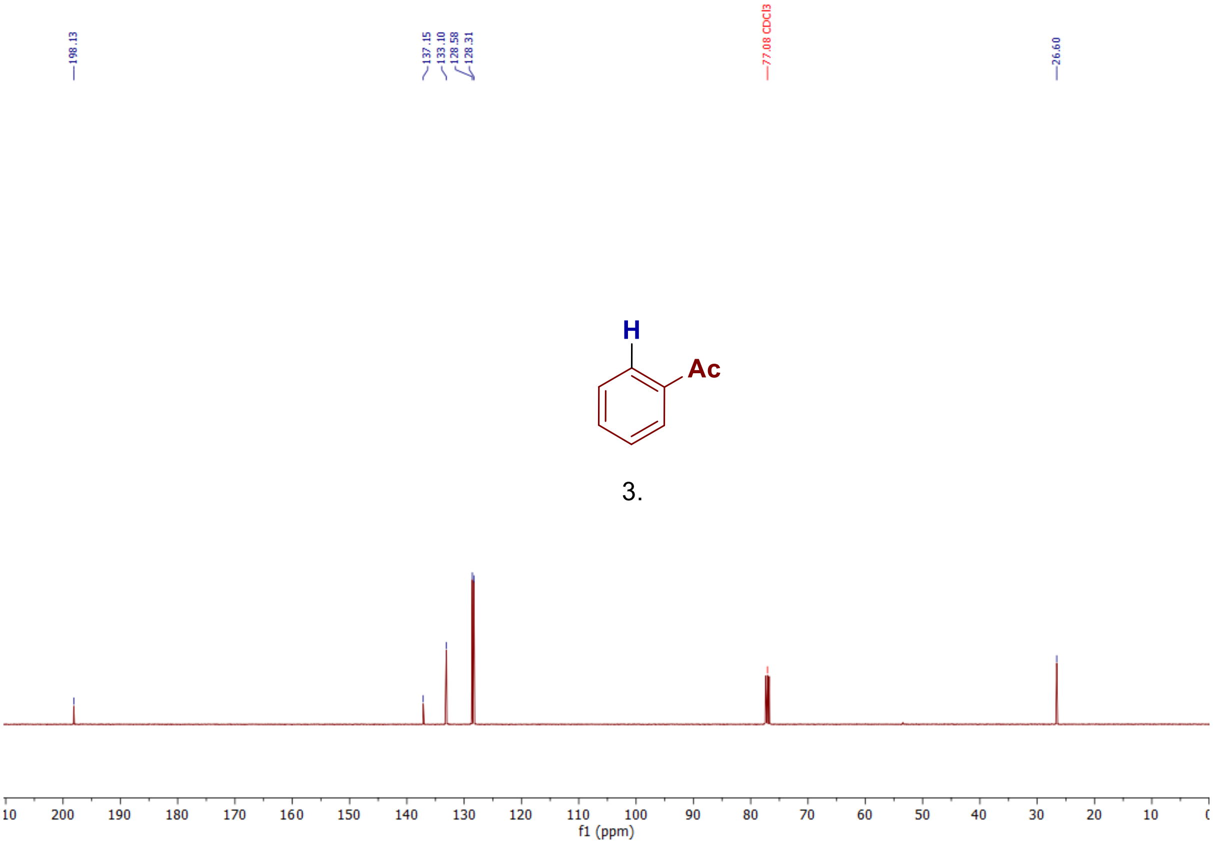

^{1}H NMR (400 MHz, $CDCl_3$)

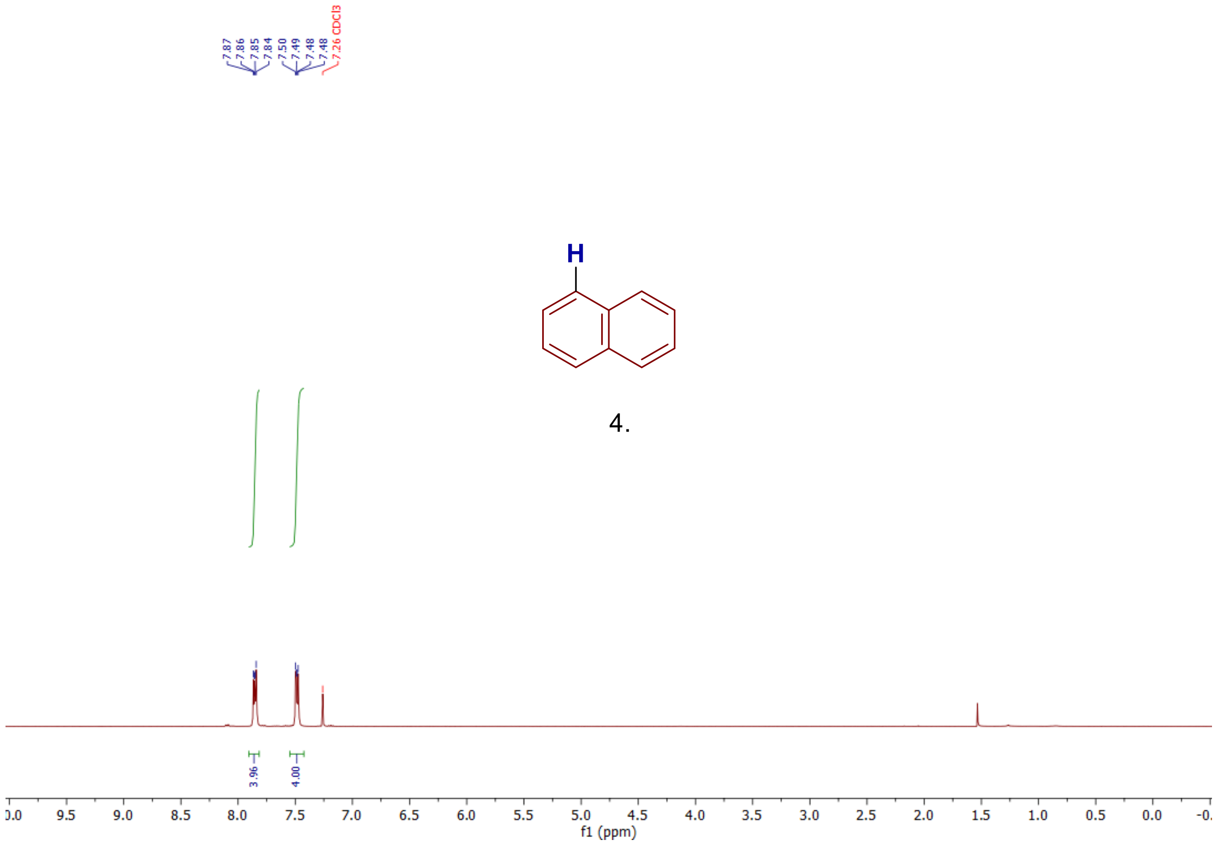

^{13}C NMR (101 MHz, $CDCl_3$)

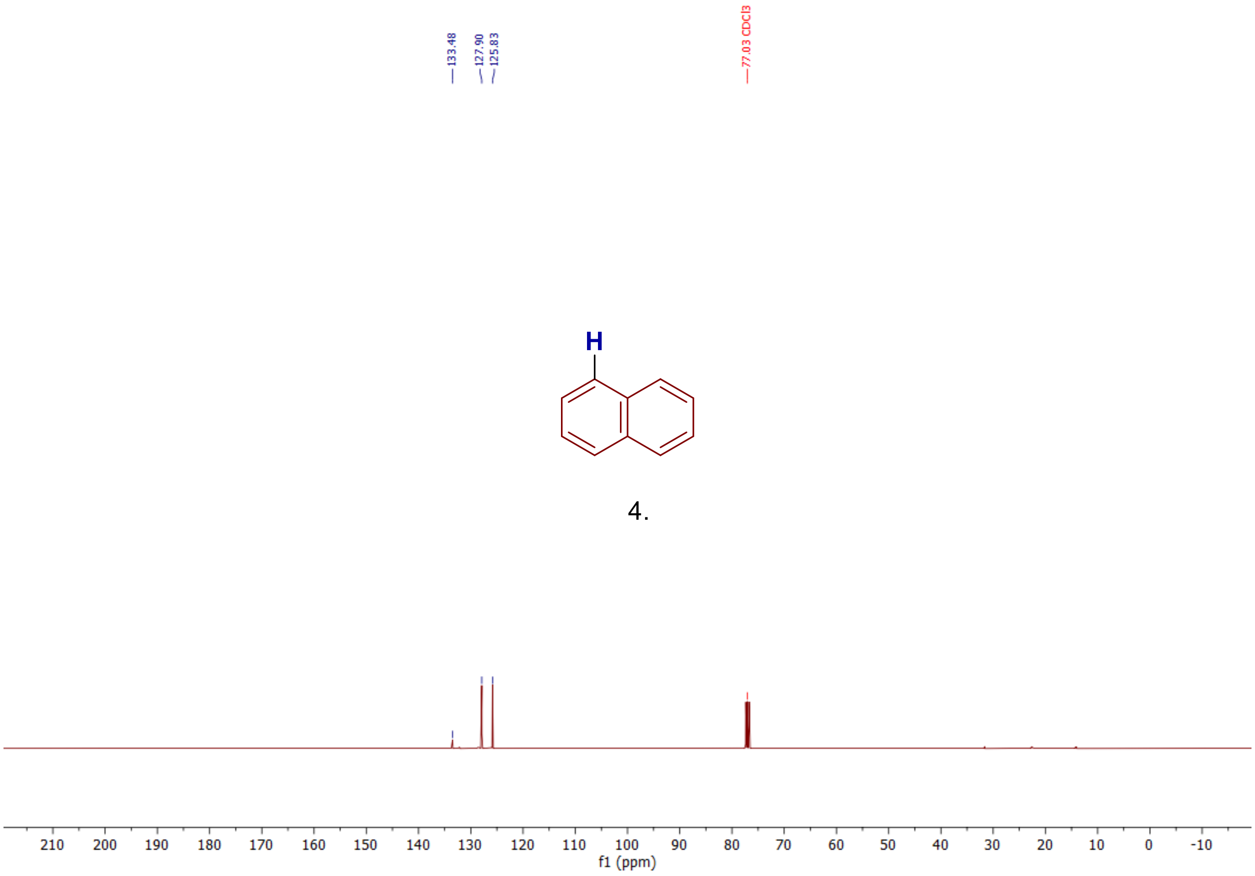

^{1}H NMR (400 MHz, $CDCl_3$)

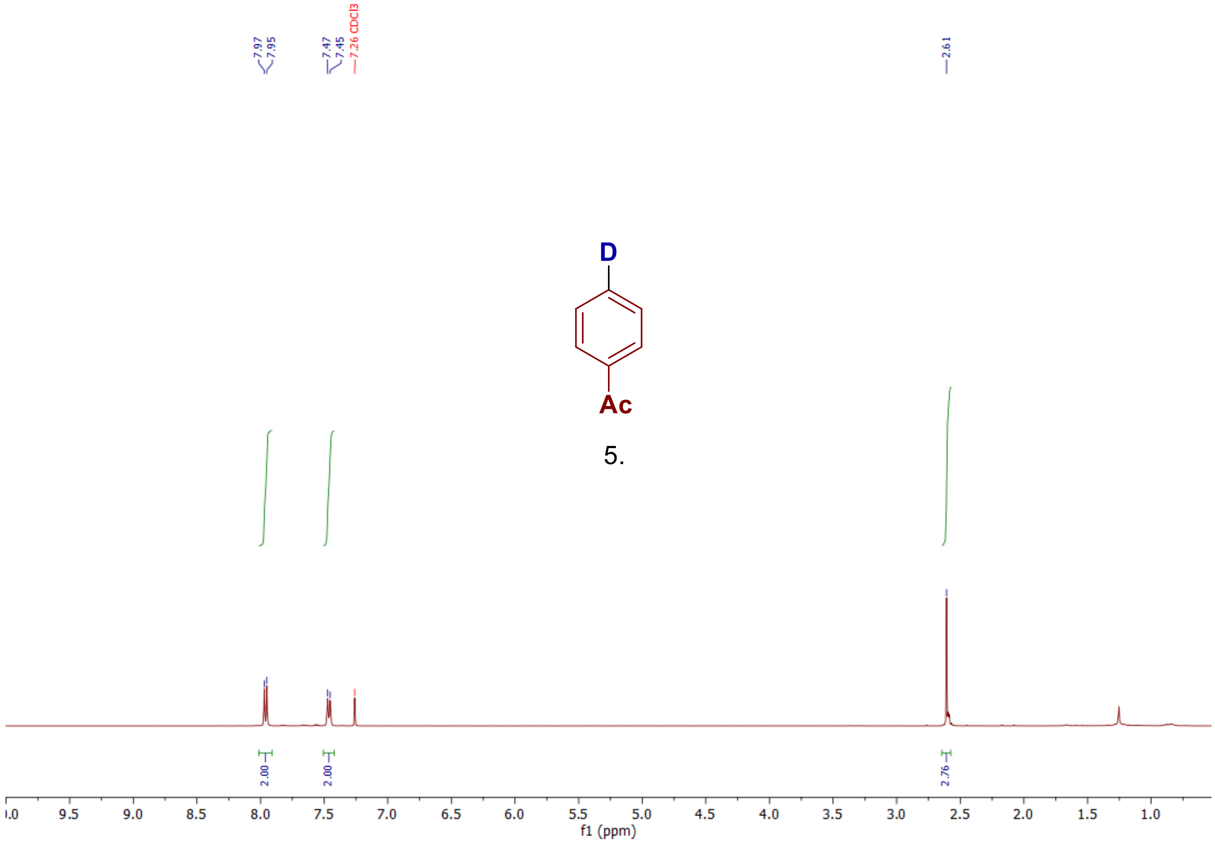

^{13}C NMR (101 MHz, $CDCl_3$)

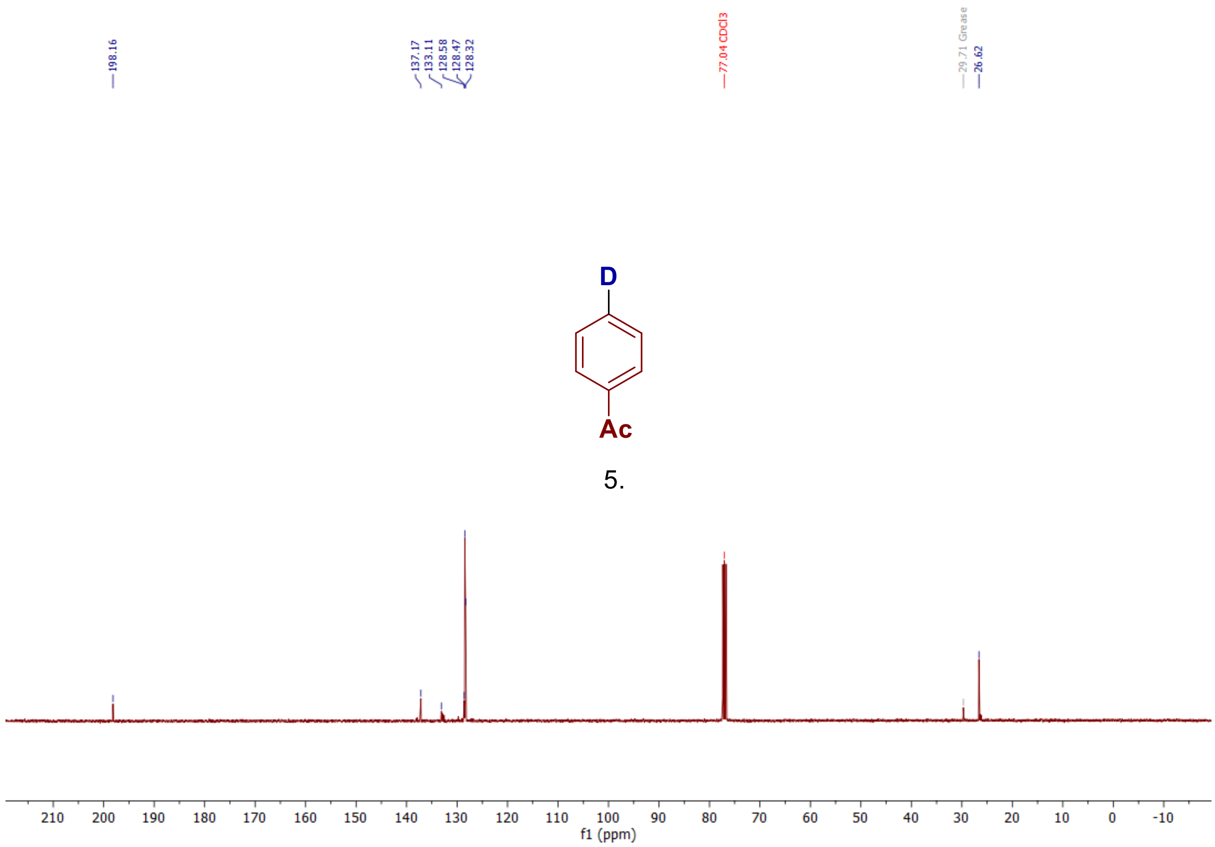

^{1}H NMR (400 MHz, $CDCl_3$)

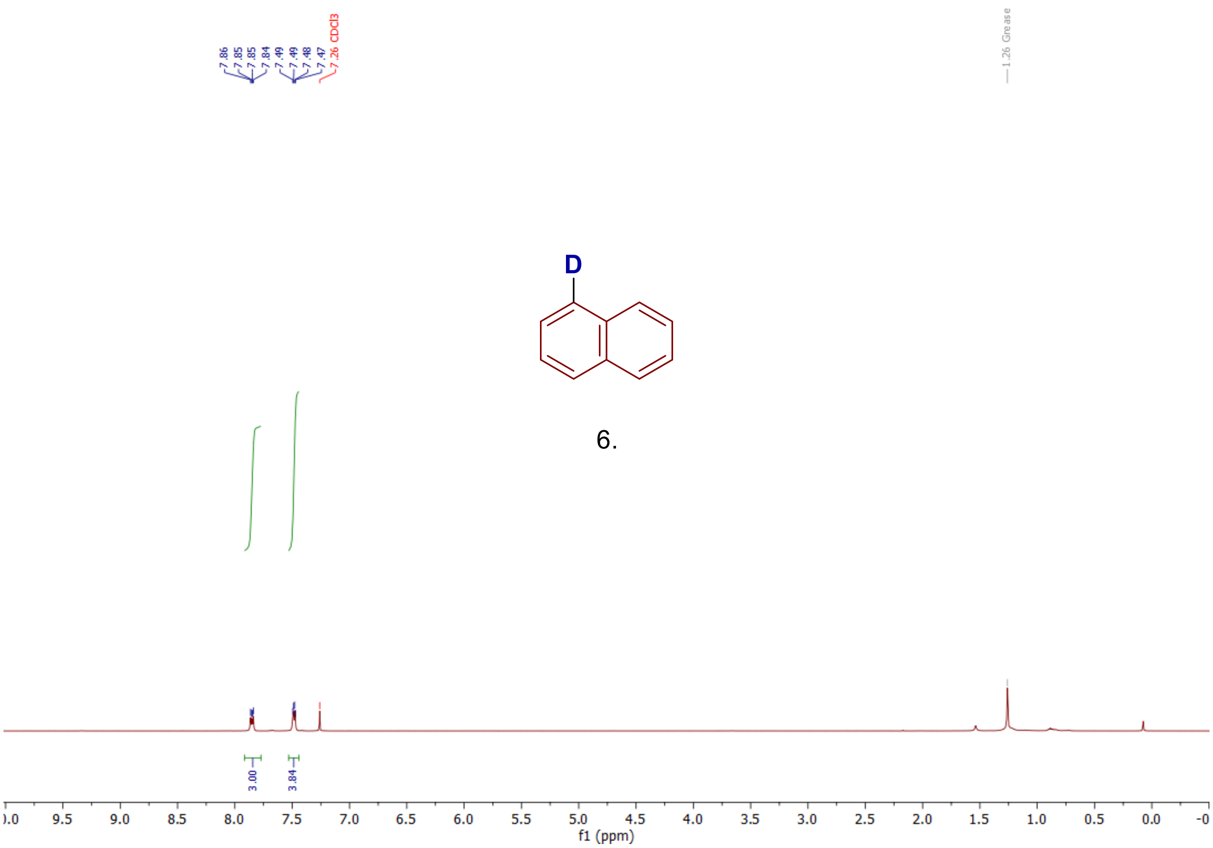

^{13}C NMR (101 MHz, $CDCl_3$)

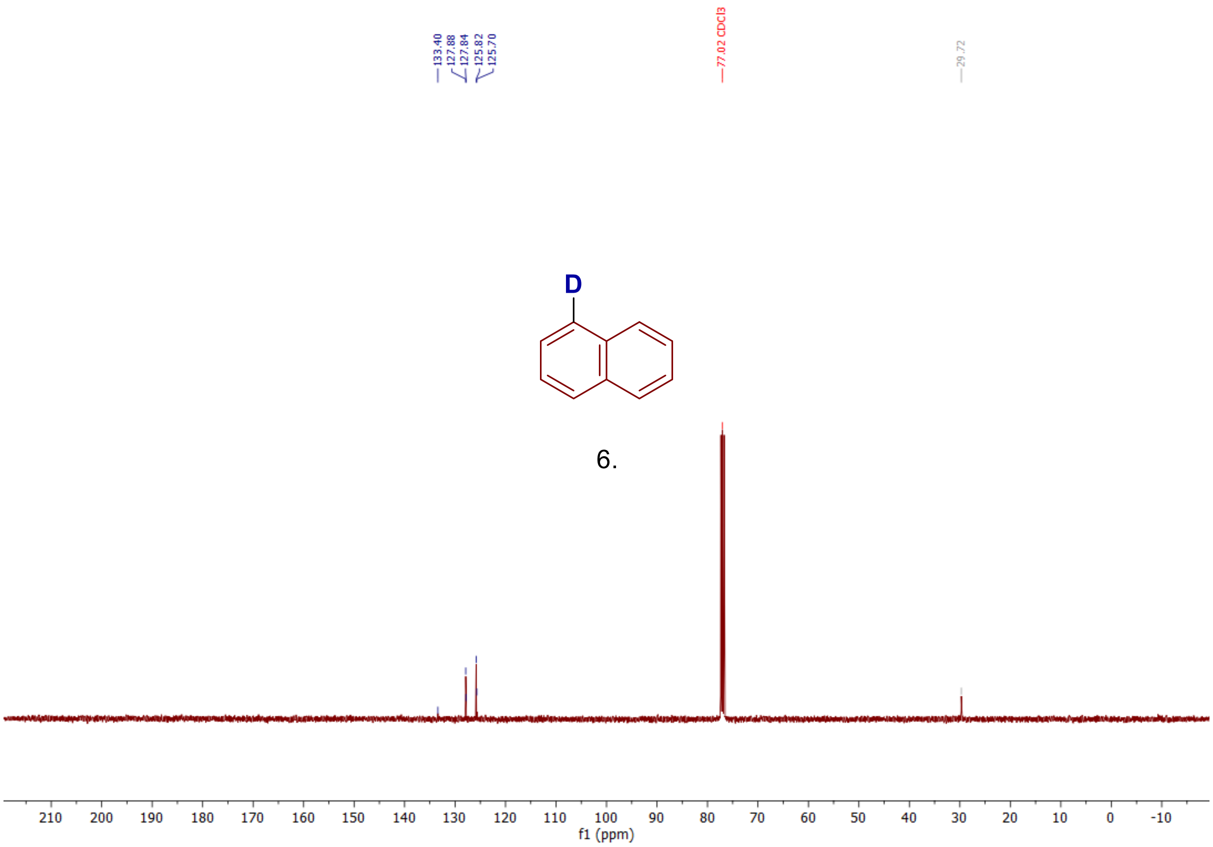

^{1}**H NMR** (400 MHz, $CDCl_3$)

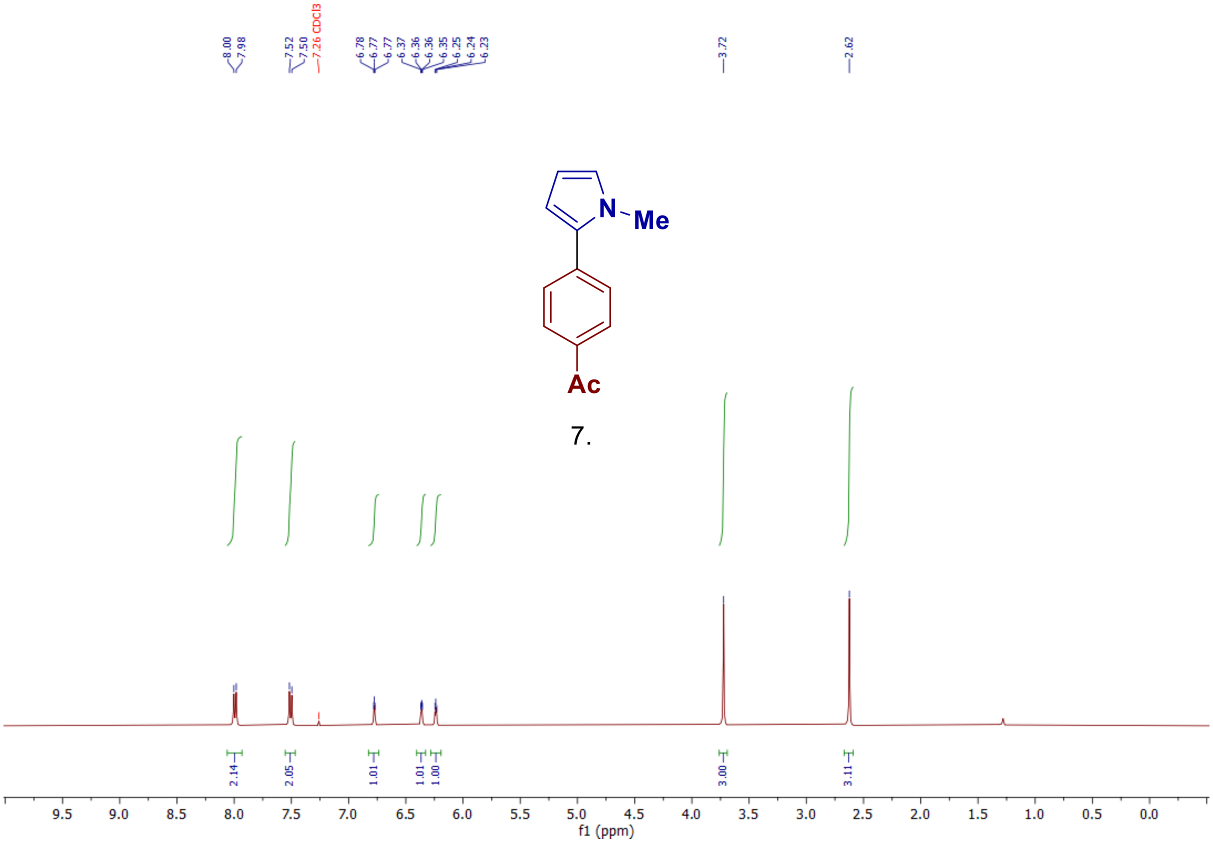

^{13}**C NMR** (101 MHz, $CDCl_3$)

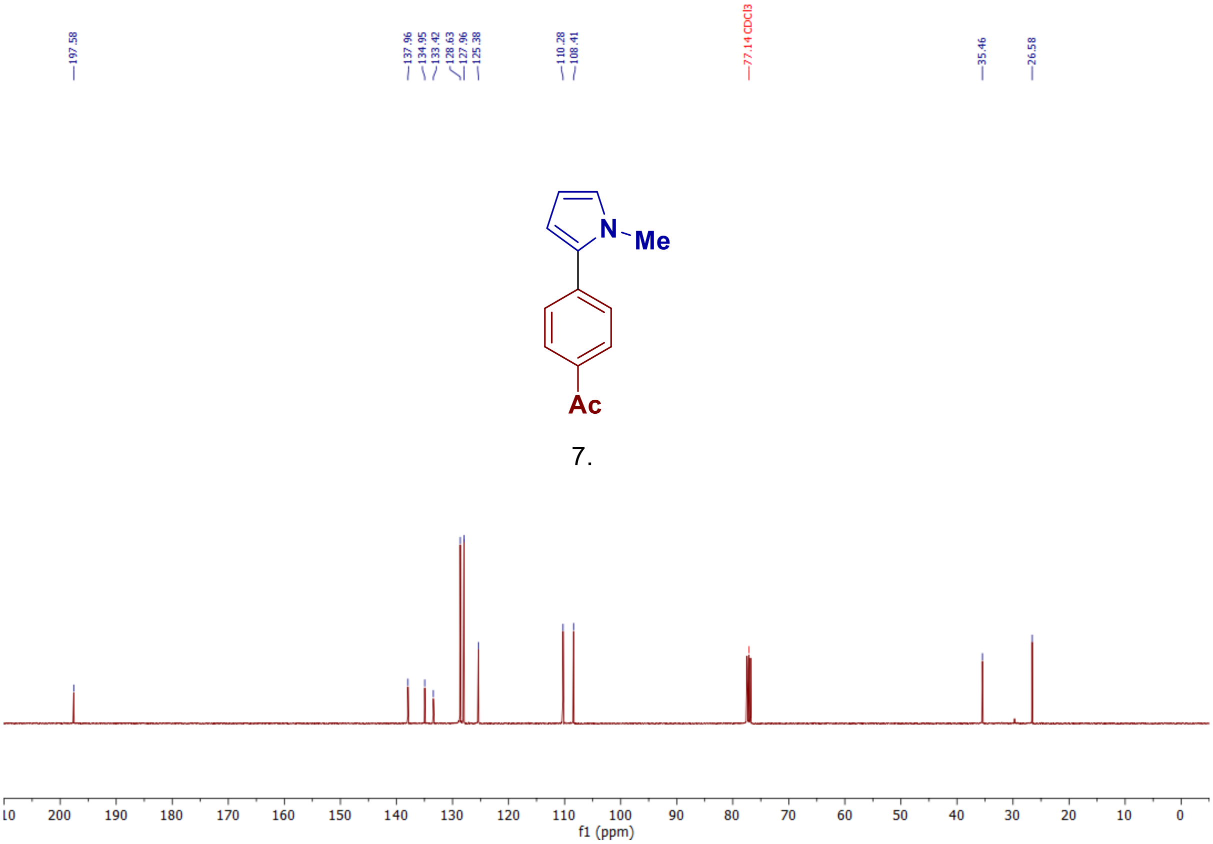

^{1}H NMR (400 MHz, $CDCl_3$)

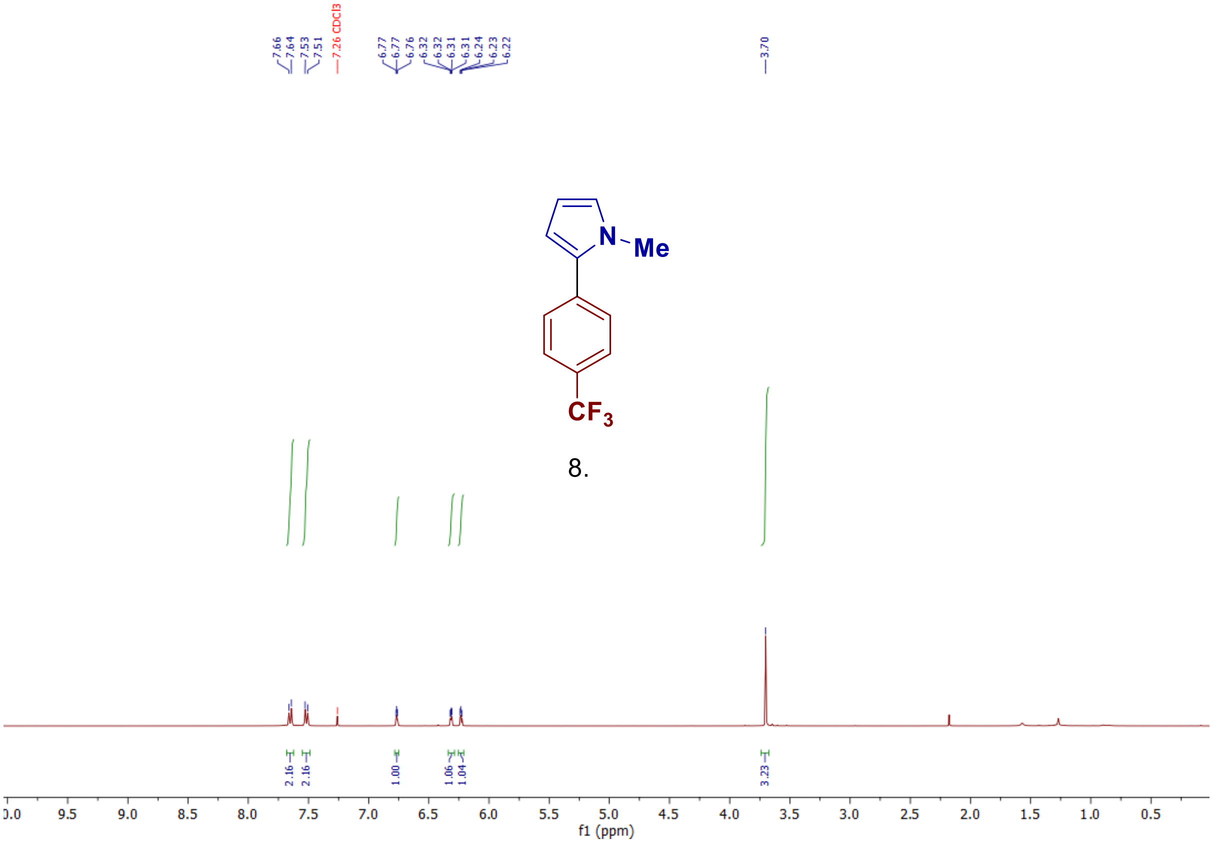

^{13}C NMR (101 MHz, $CDCl_3$)

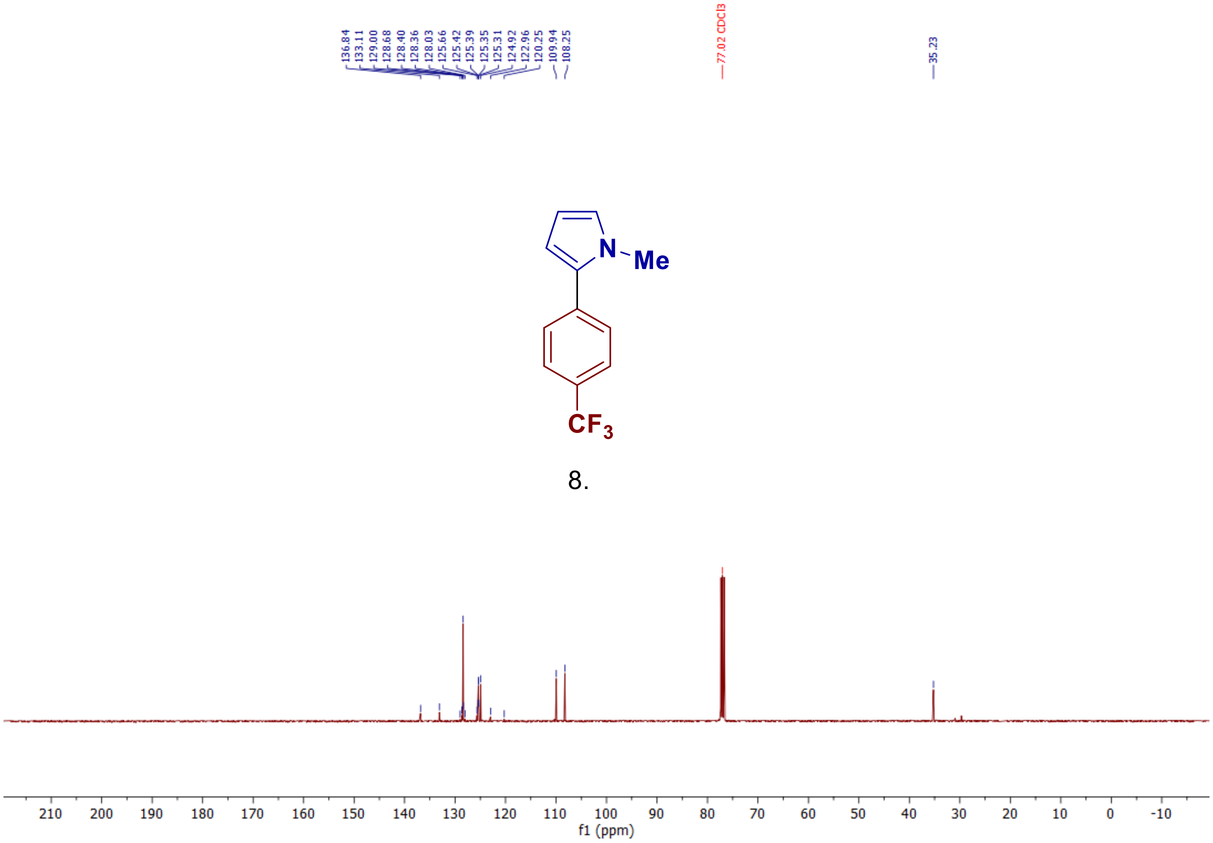

^{19}F NMR (377 MHz, $CDCl_3$)

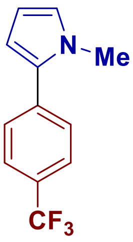

8.

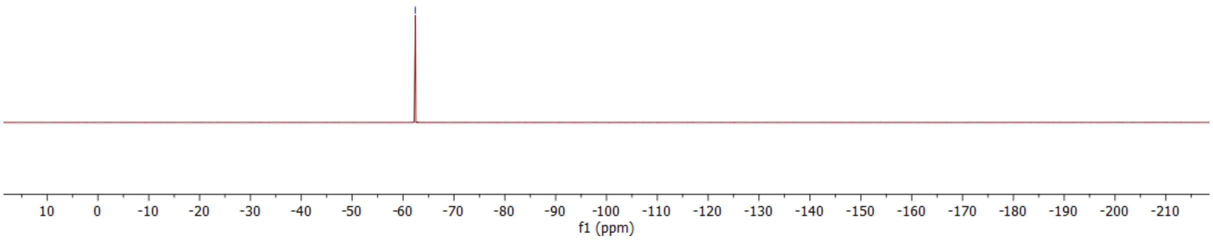

[1]**H NMR** (400 MHz, $CDCl_3$)

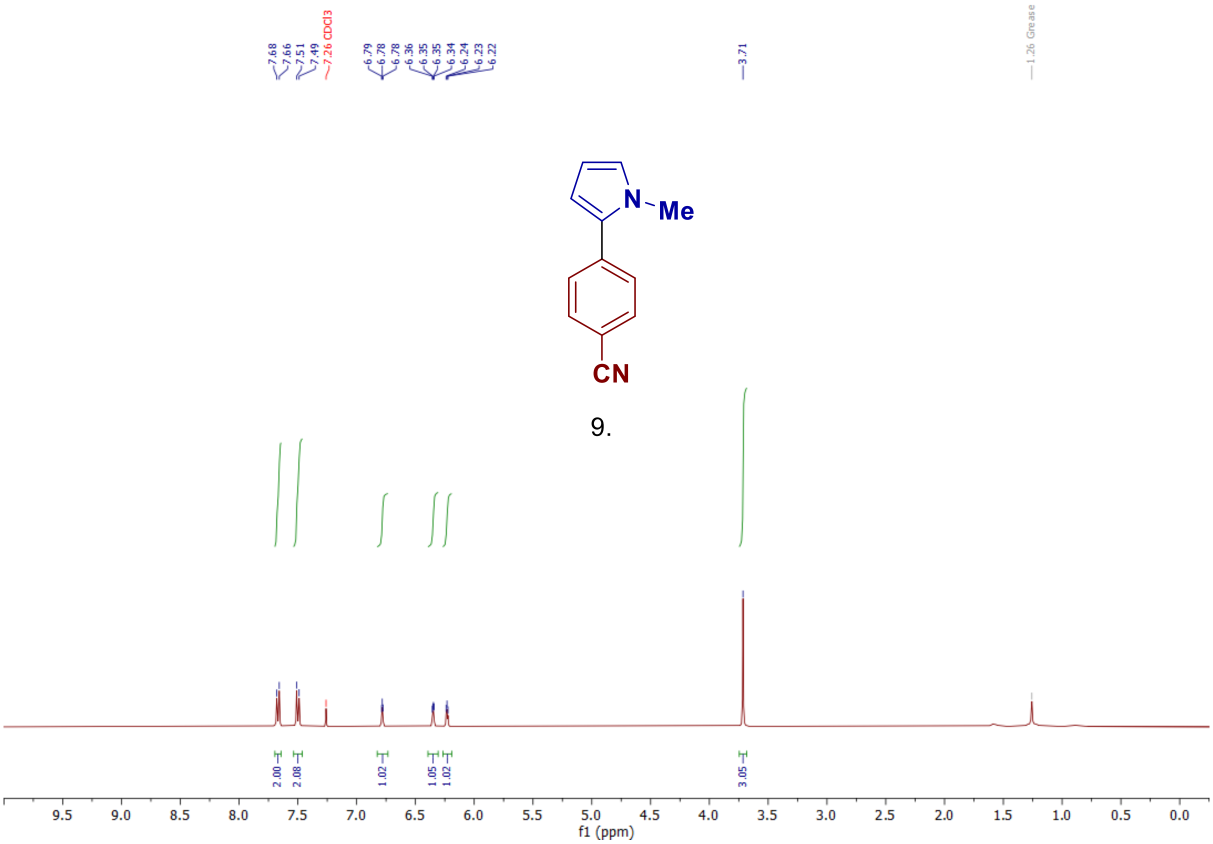

[13]**C NMR** (101 MHz, $CDCl_3$)

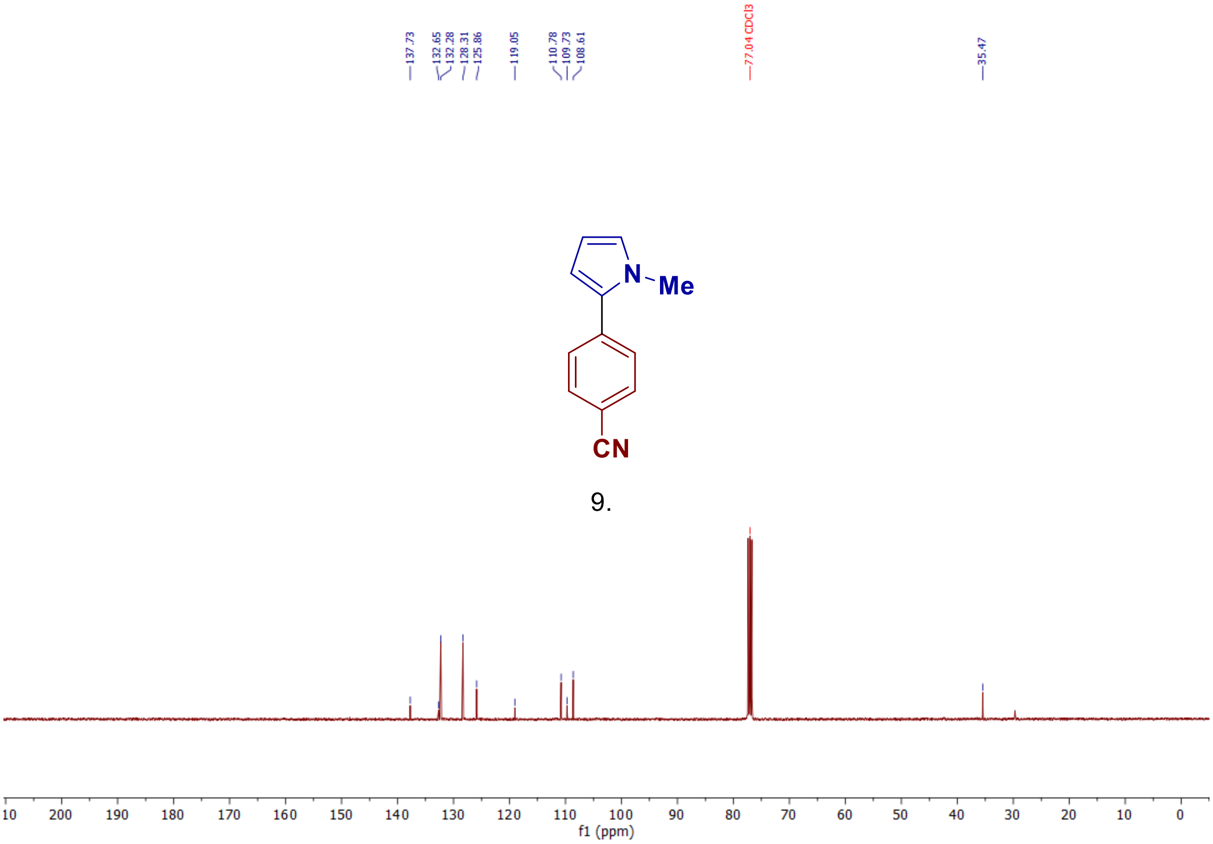

^{1}H NMR (400 MHz, $CDCl_3$)

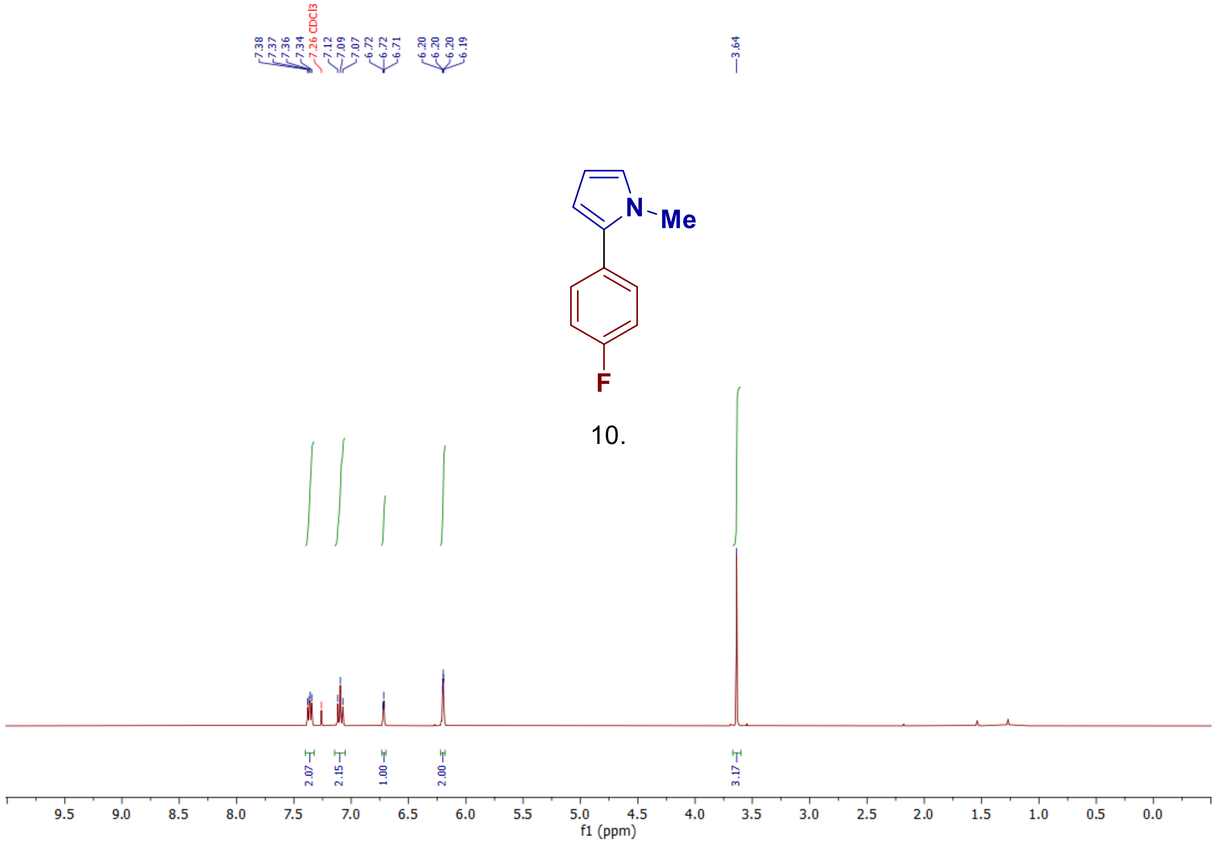

^{13}C NMR (101 MHz, $CDCl_3$)

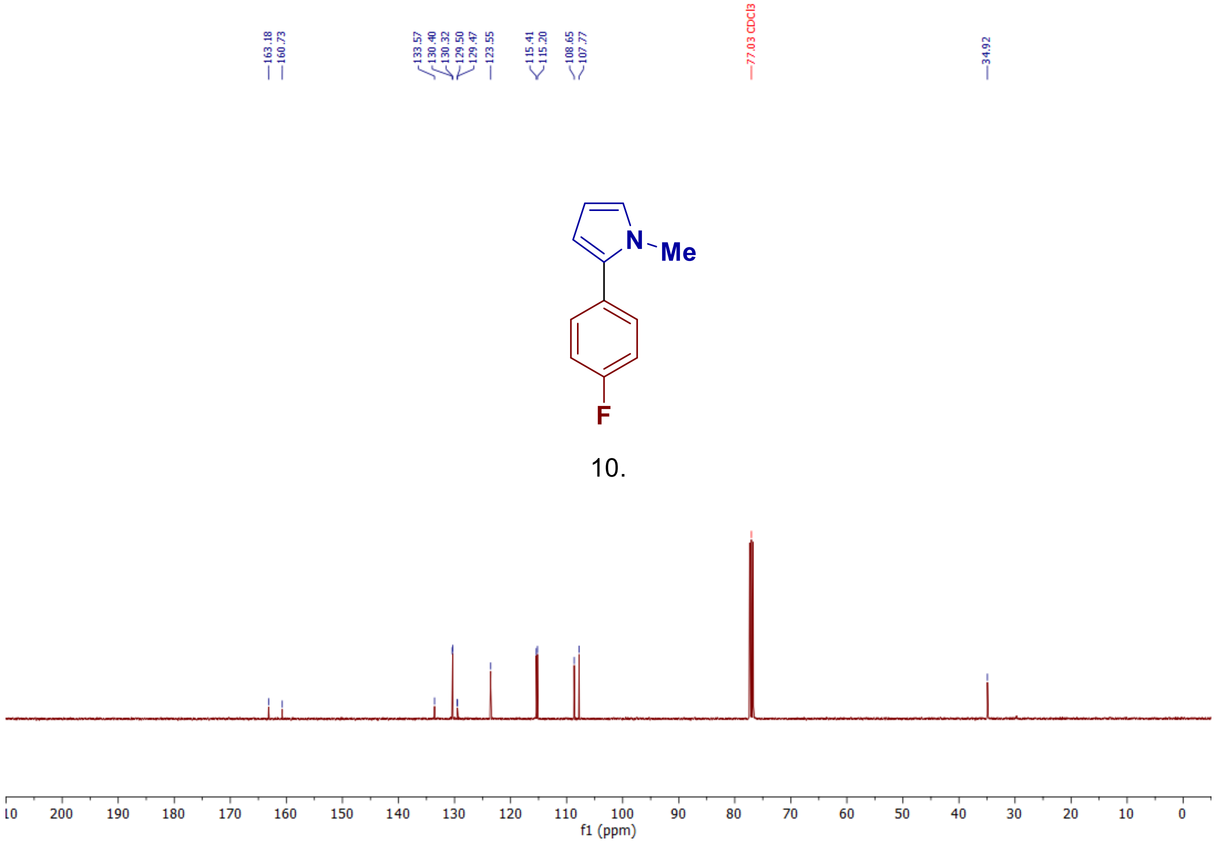

^{1}H NMR (400 MHz, $CDCl_3$)

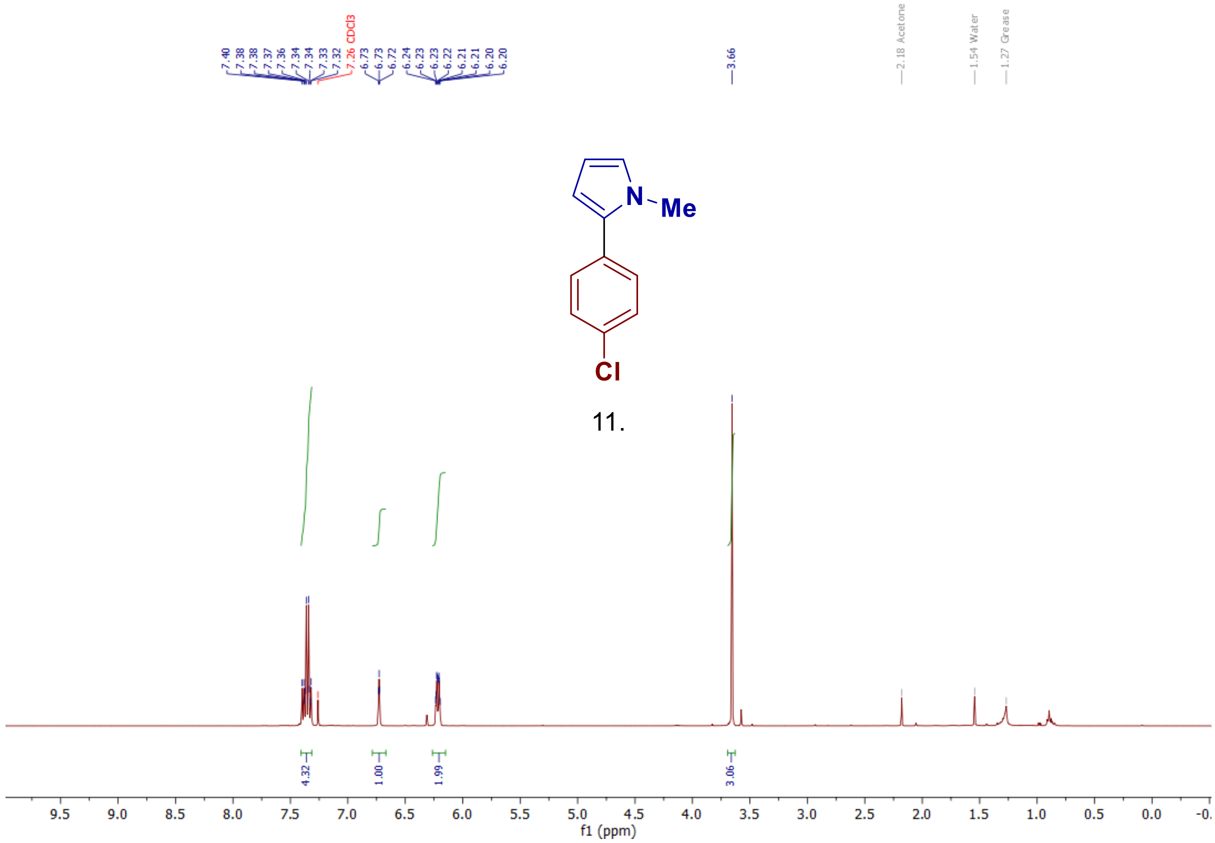

^{13}C NMR (101 MHz, $CDCl_3$)

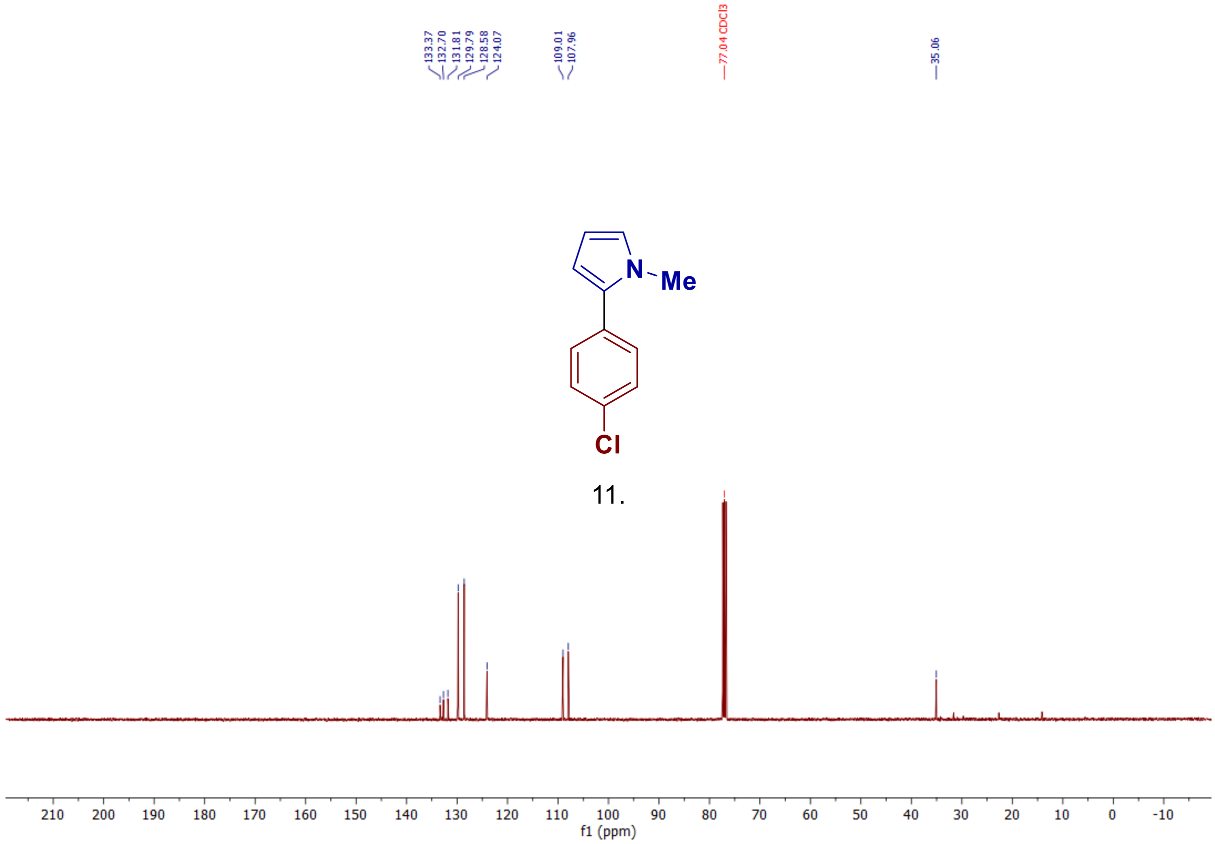

^{1}H NMR (400 MHz, $CDCl_3$)

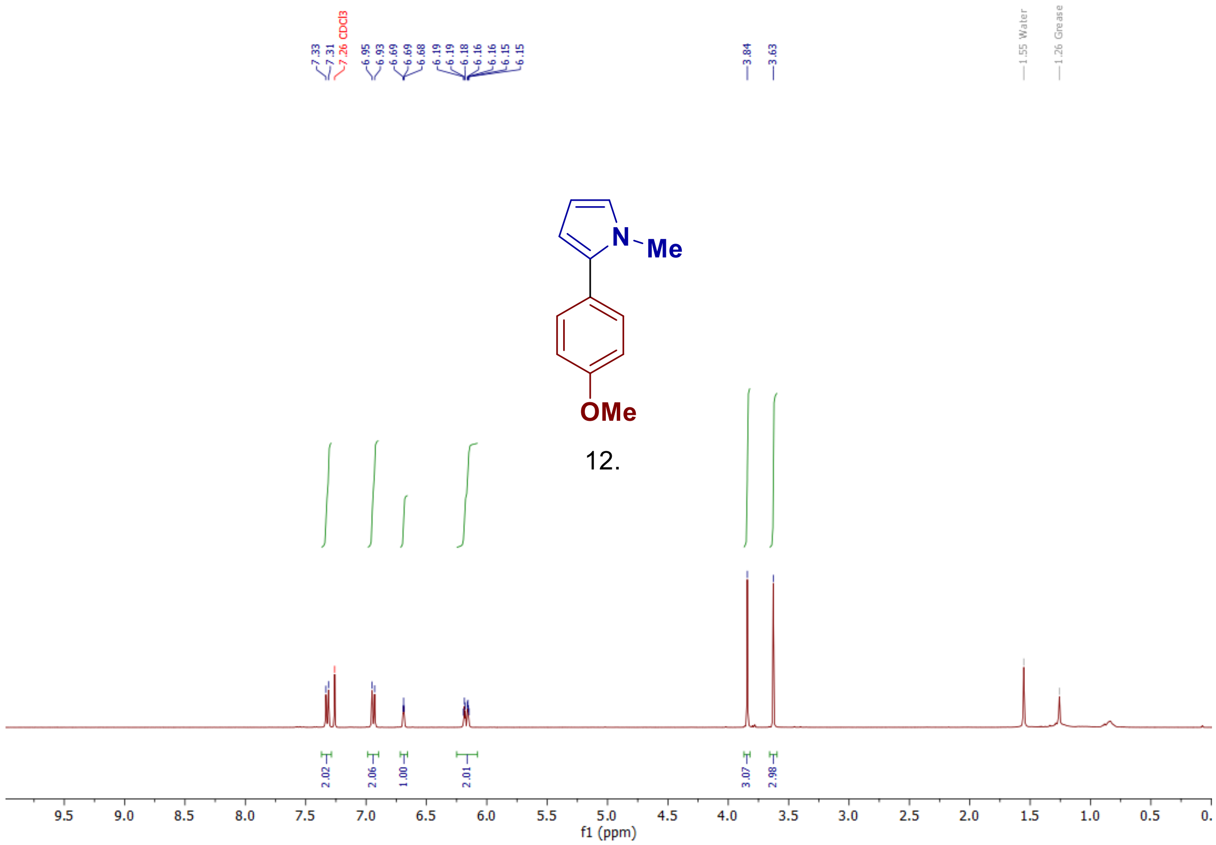

^{13}C NMR (101 MHz, $CDCl_3$)

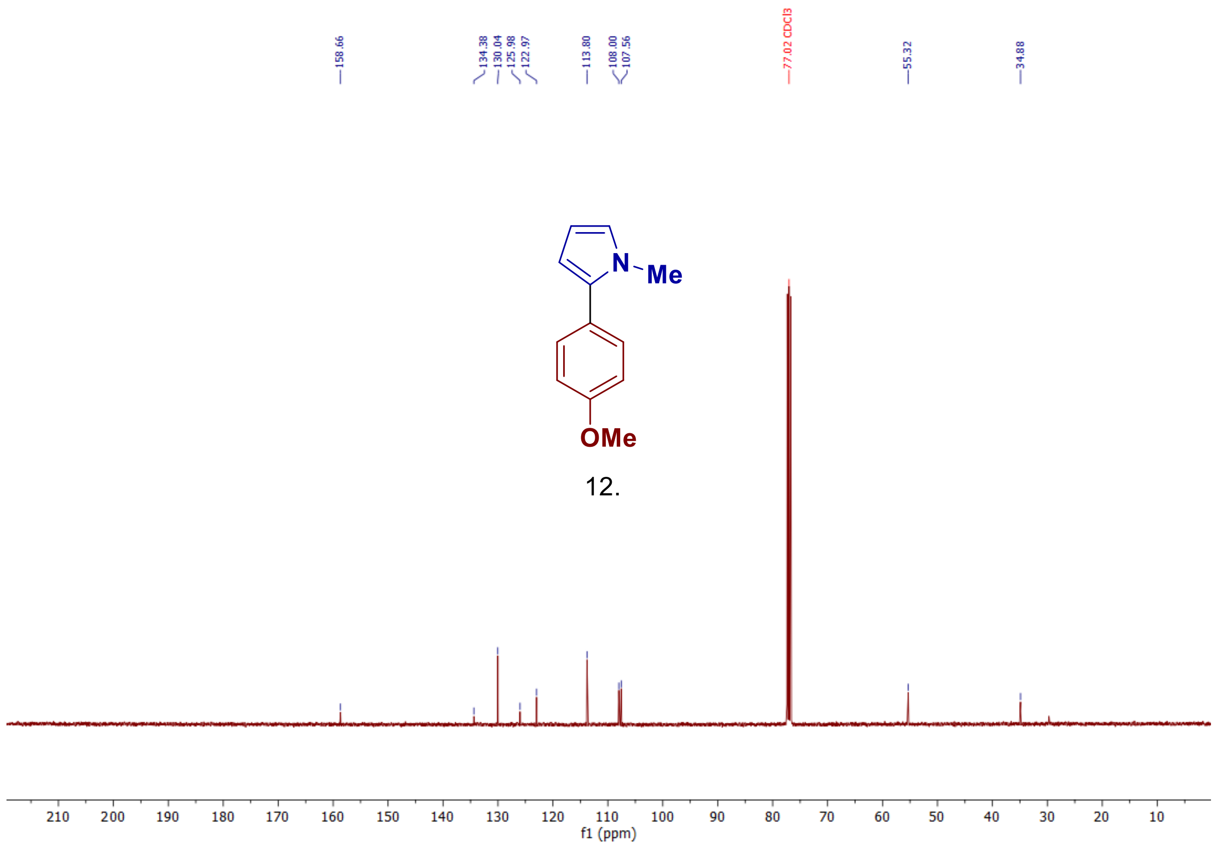

^{1}H NMR (400 MHz, $CDCl_3$)

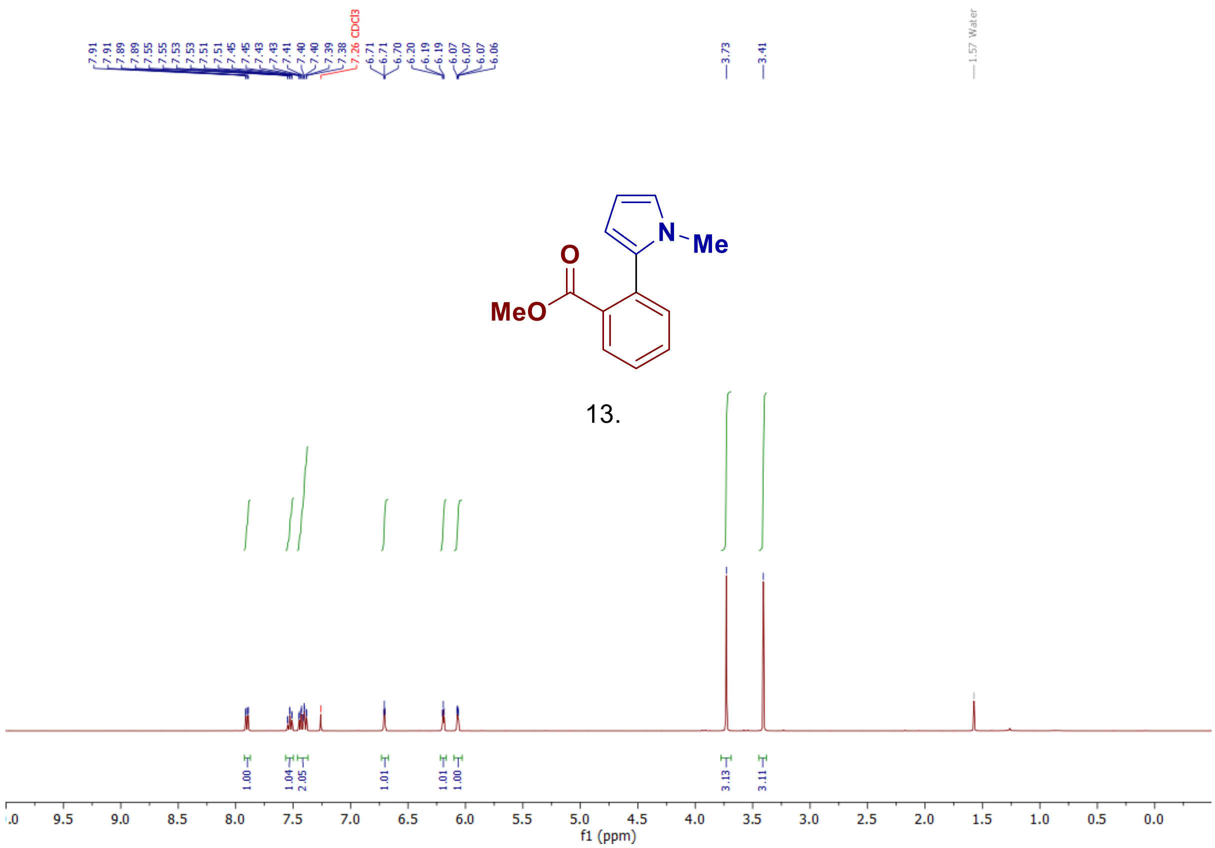

^{13}C NMR (101 MHz, $CDCl_3$)

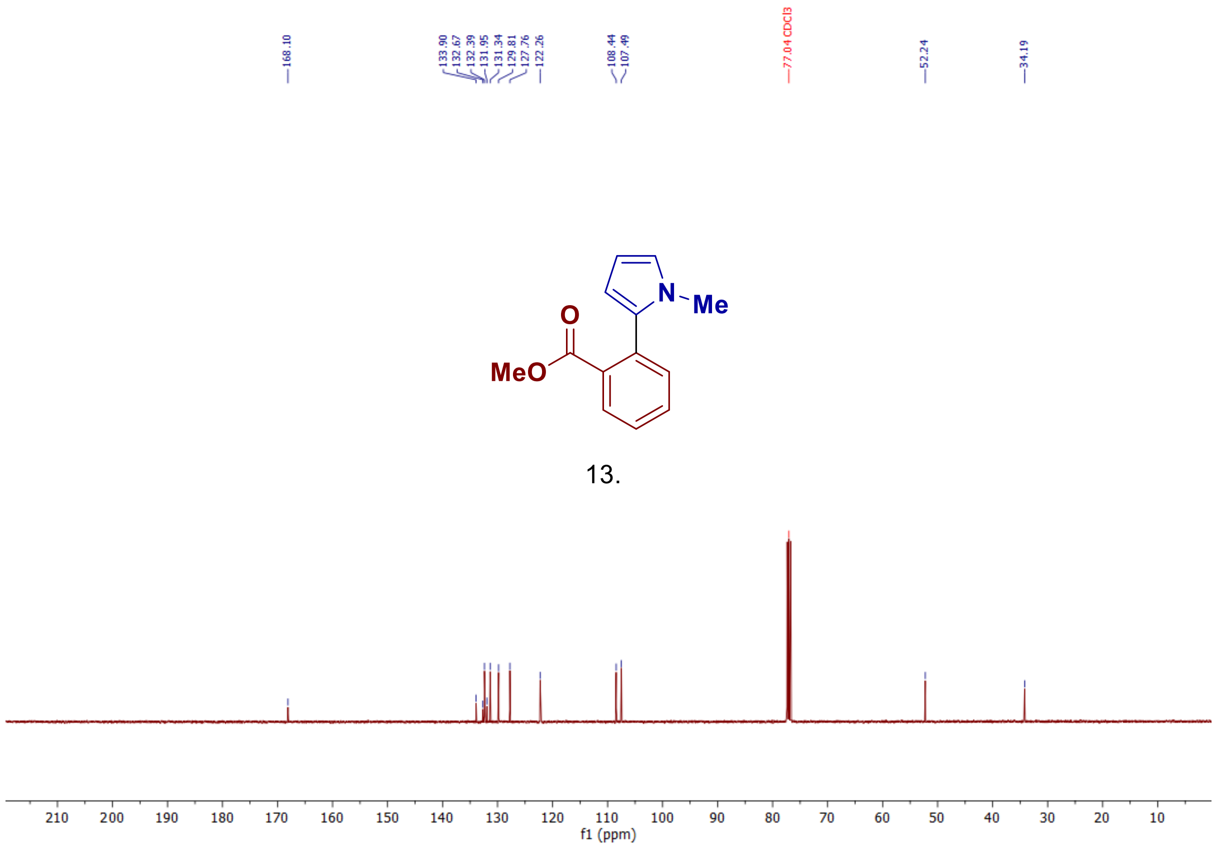

^{1}H NMR (400 MHz, $CDCl_3$)

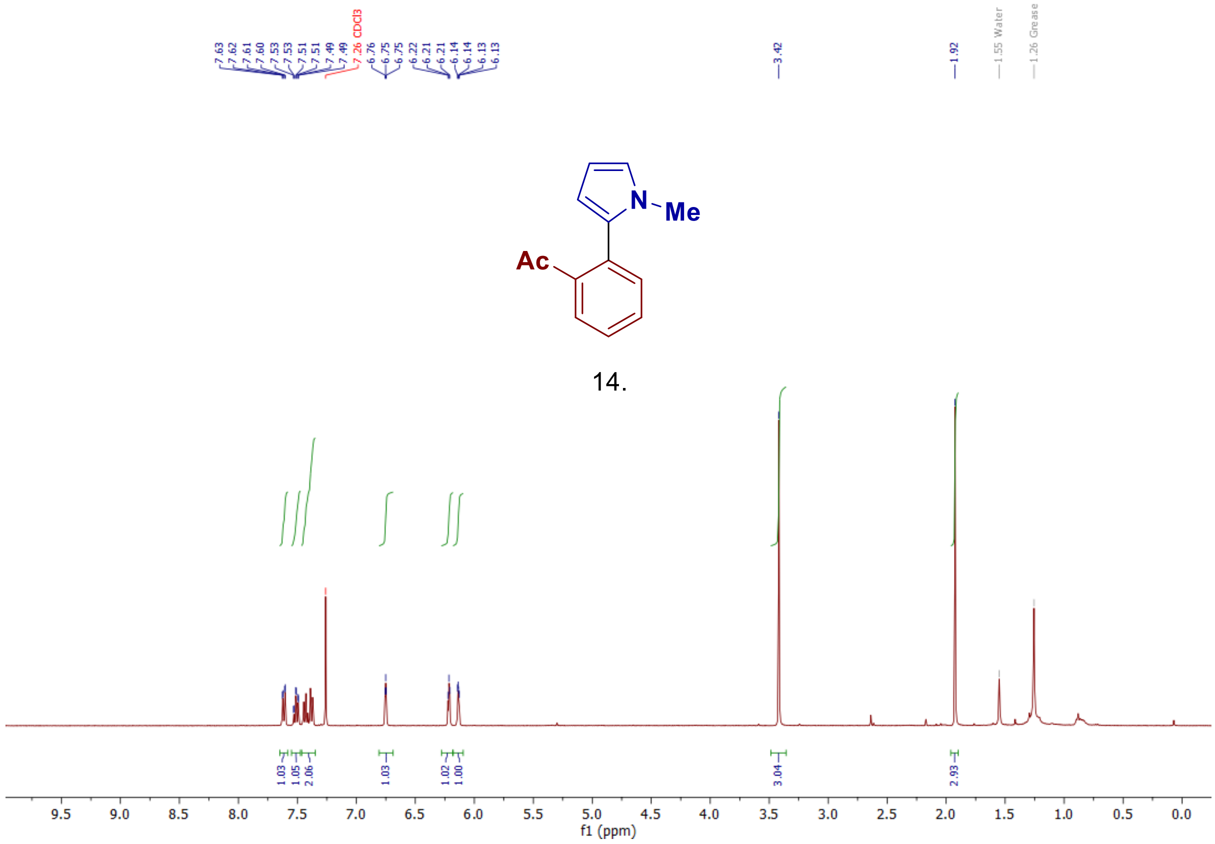

^{13}C NMR (101 MHz, $CDCl_3$)

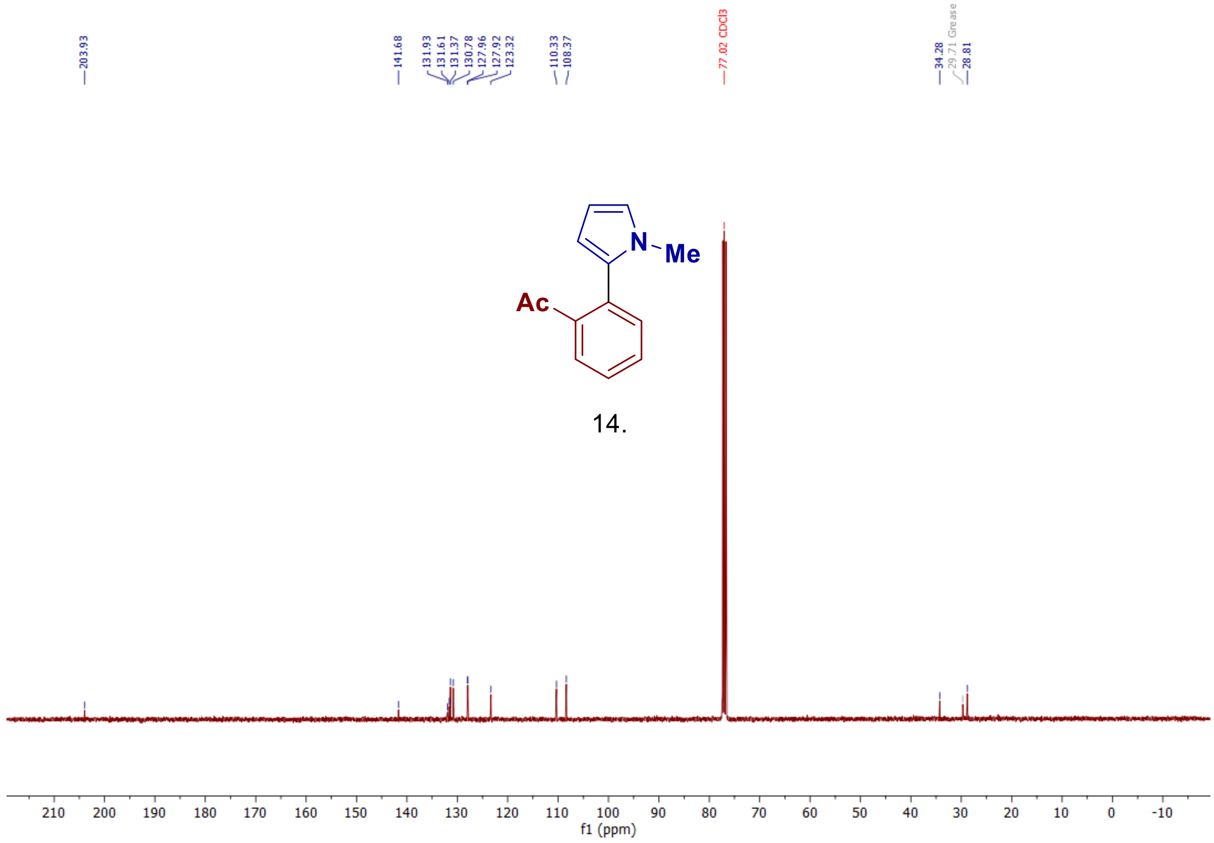

^{1}**H NMR** (400 MHz, $CDCl_3$)

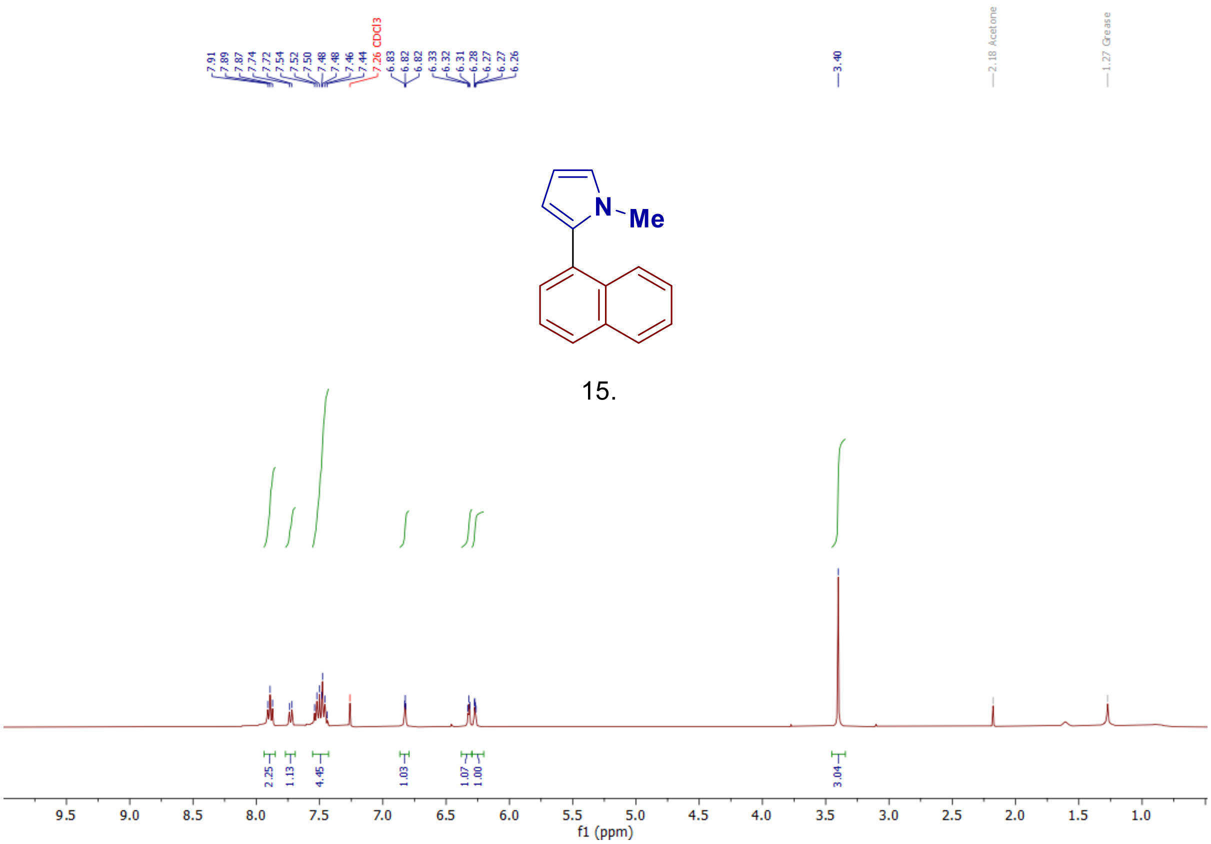

^{13}**C NMR** (101 MHz, $CDCl_3$)

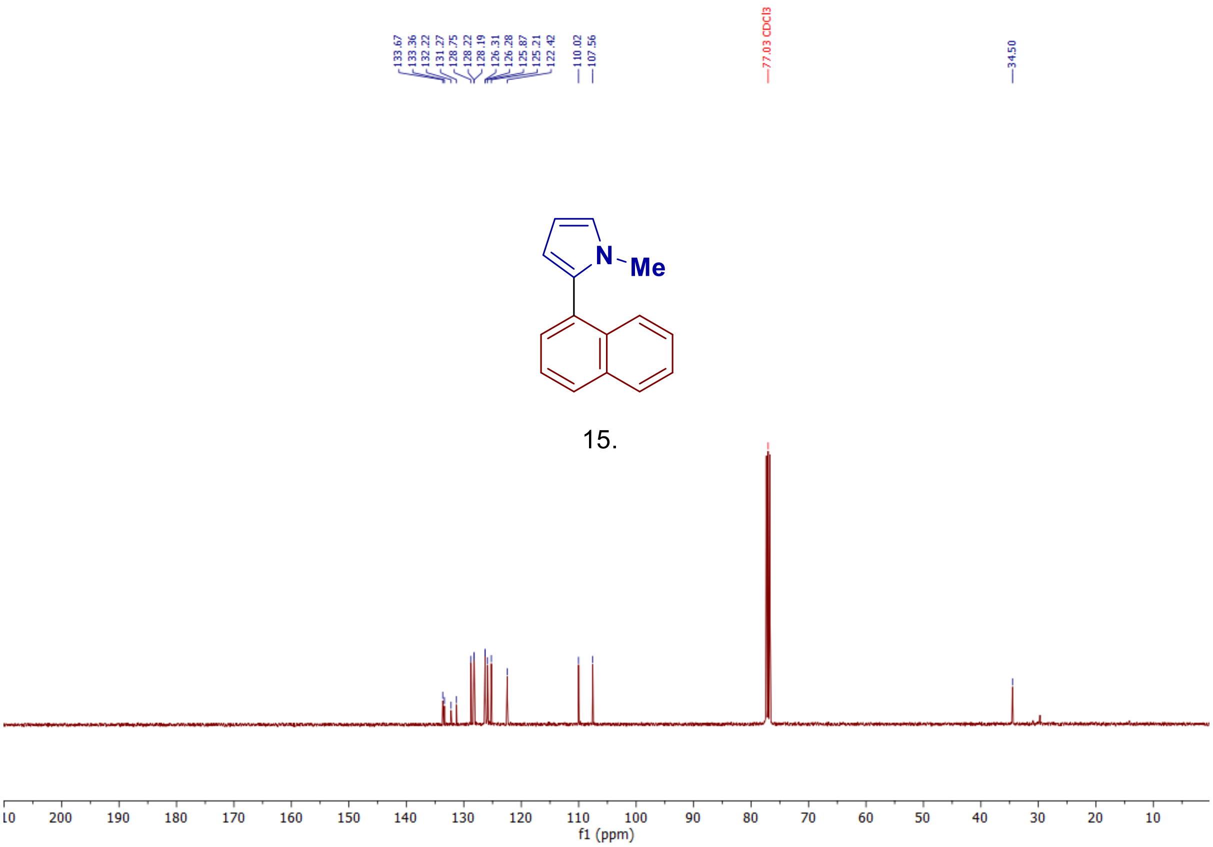

^{1}H NMR (400 MHz, $CDCl_3$)

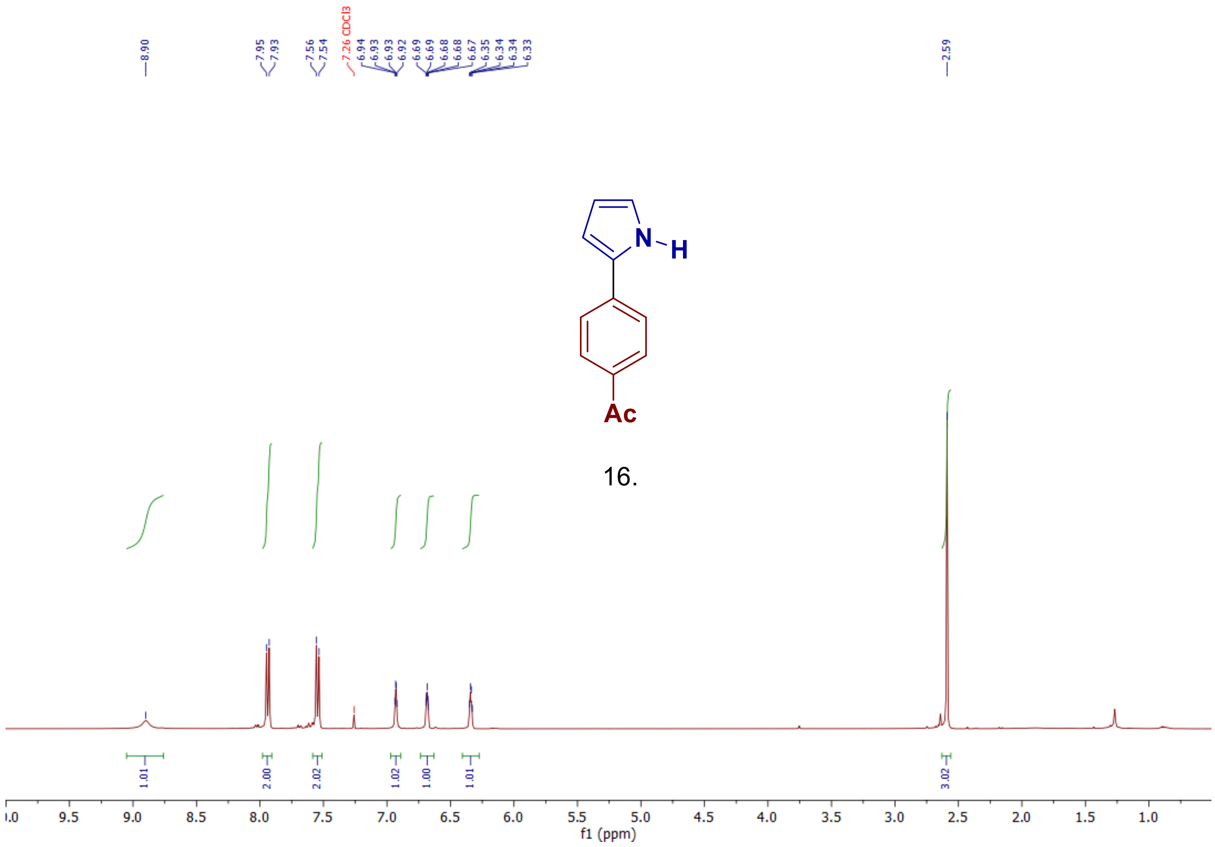

16.

^{13}C NMR (101 MHz, $CDCl_3$)

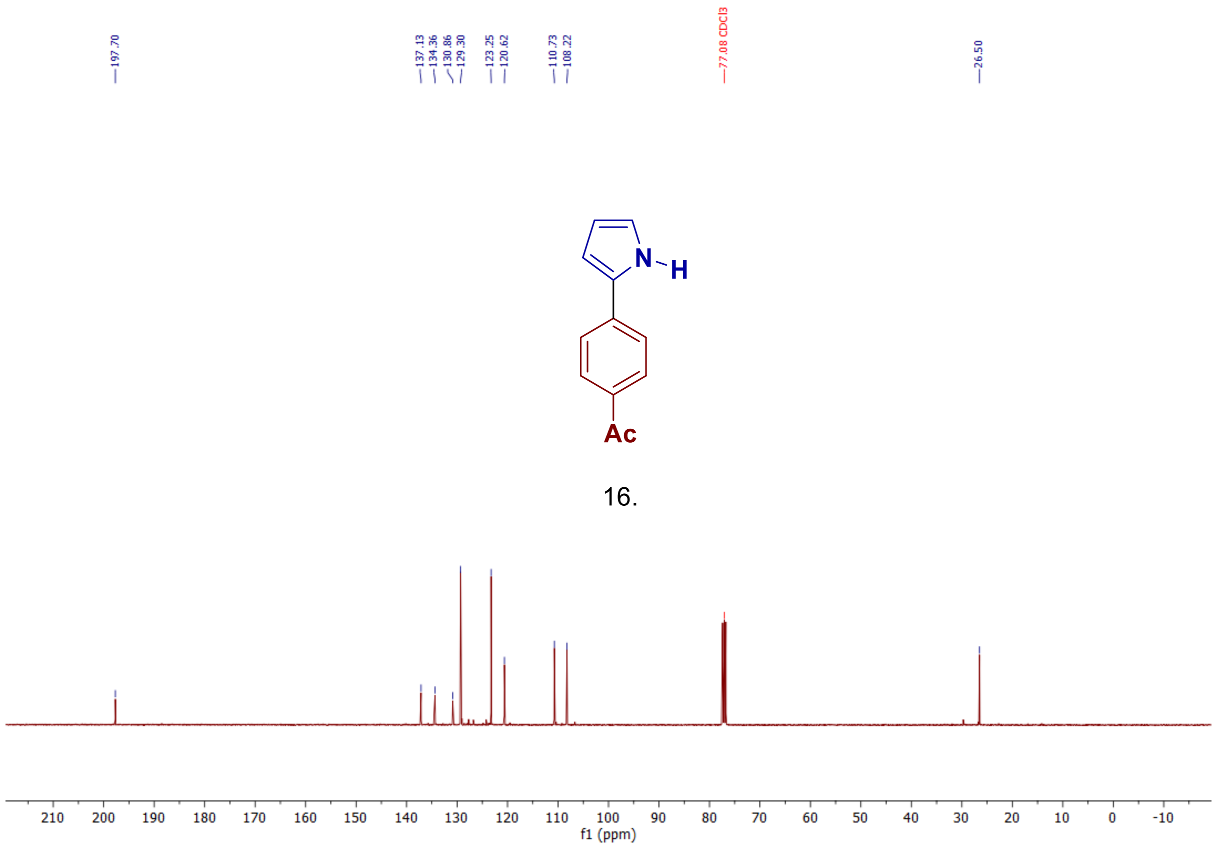

16.

^{1}H NMR (400 MHz, $CDCl_3$)

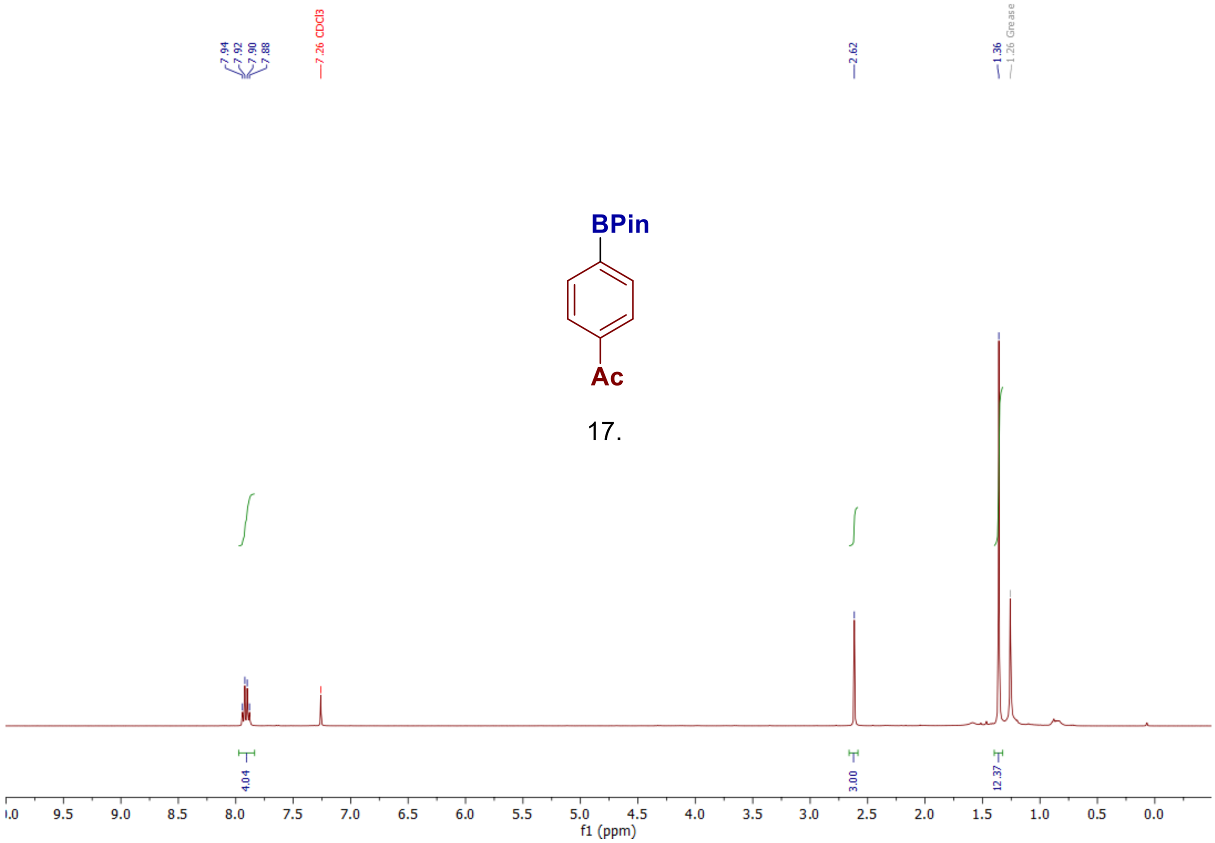

^{13}C NMR (101 MHz, $CDCl_3$)

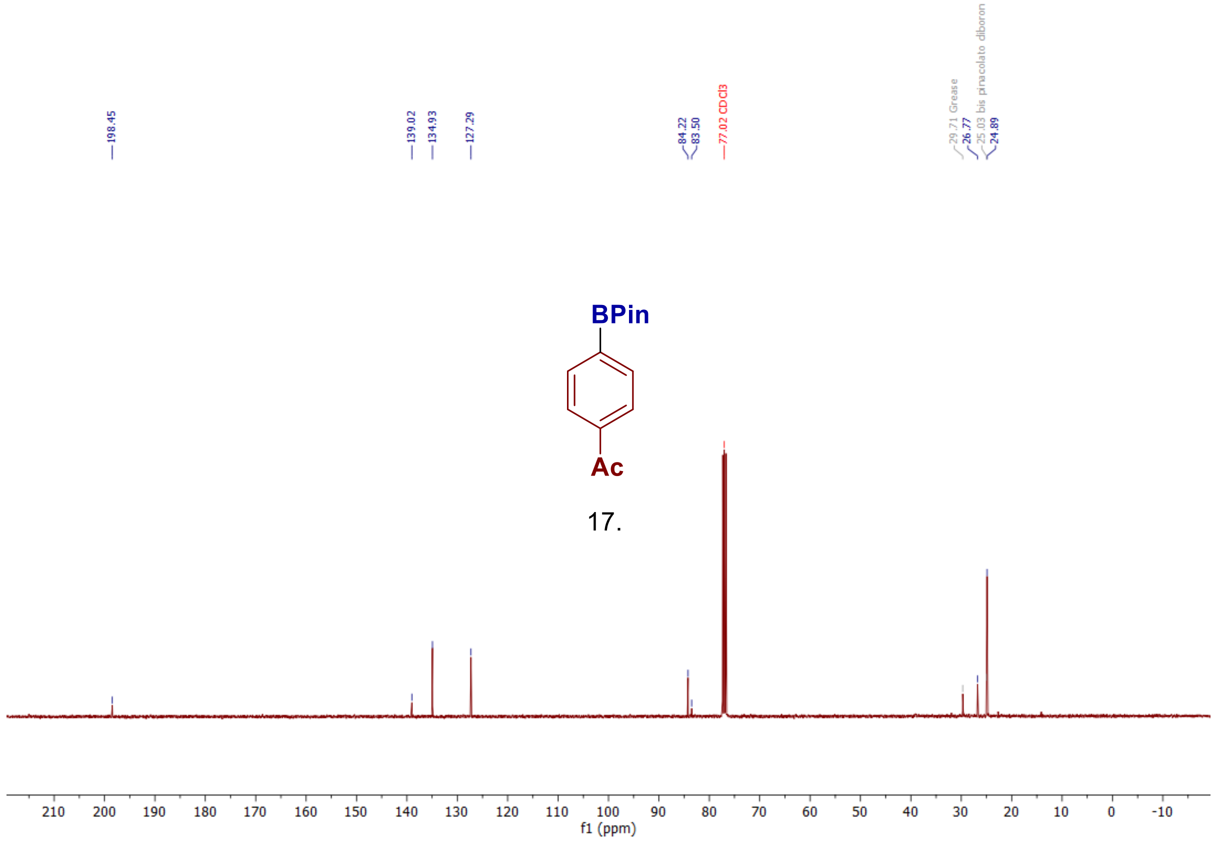

^{1}H NMR (400 MHz, $CDCl_3$)

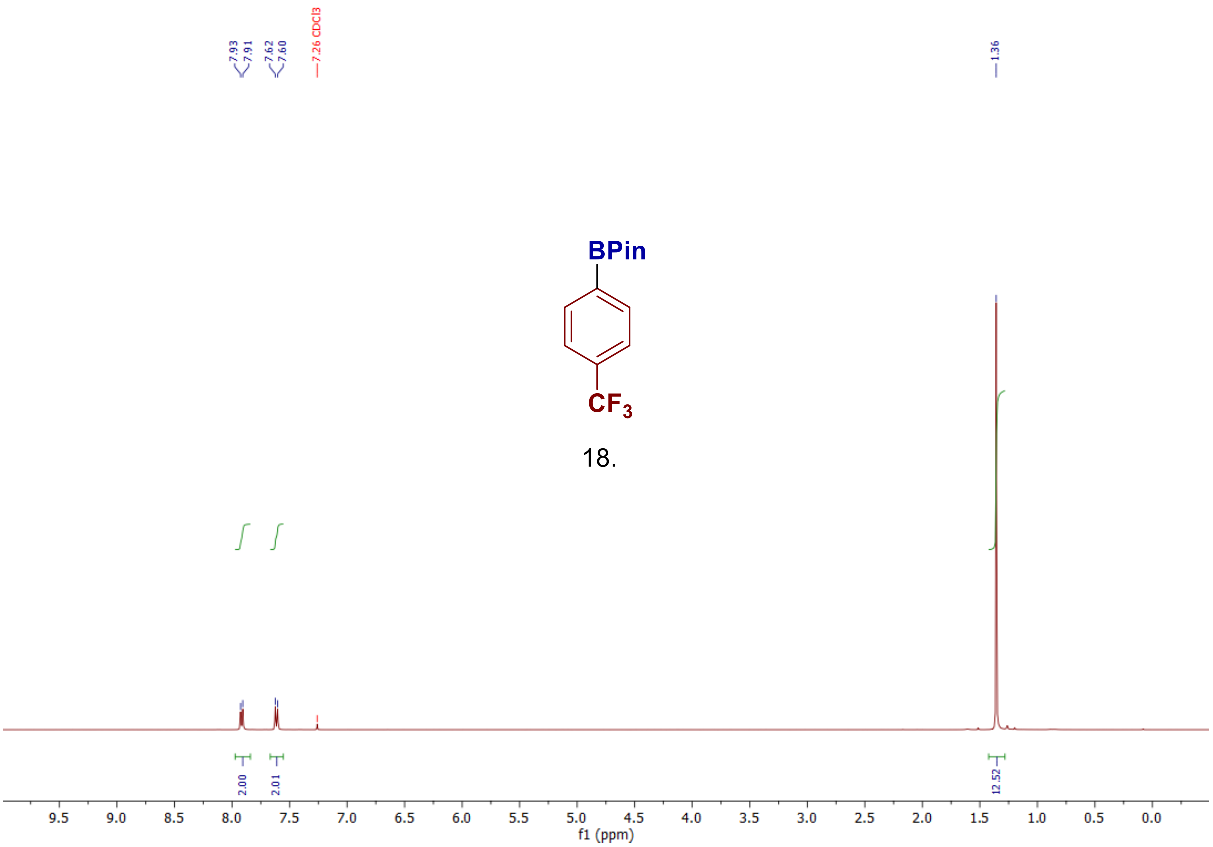

^{13}C NMR (101 MHz, $CDCl_3$)

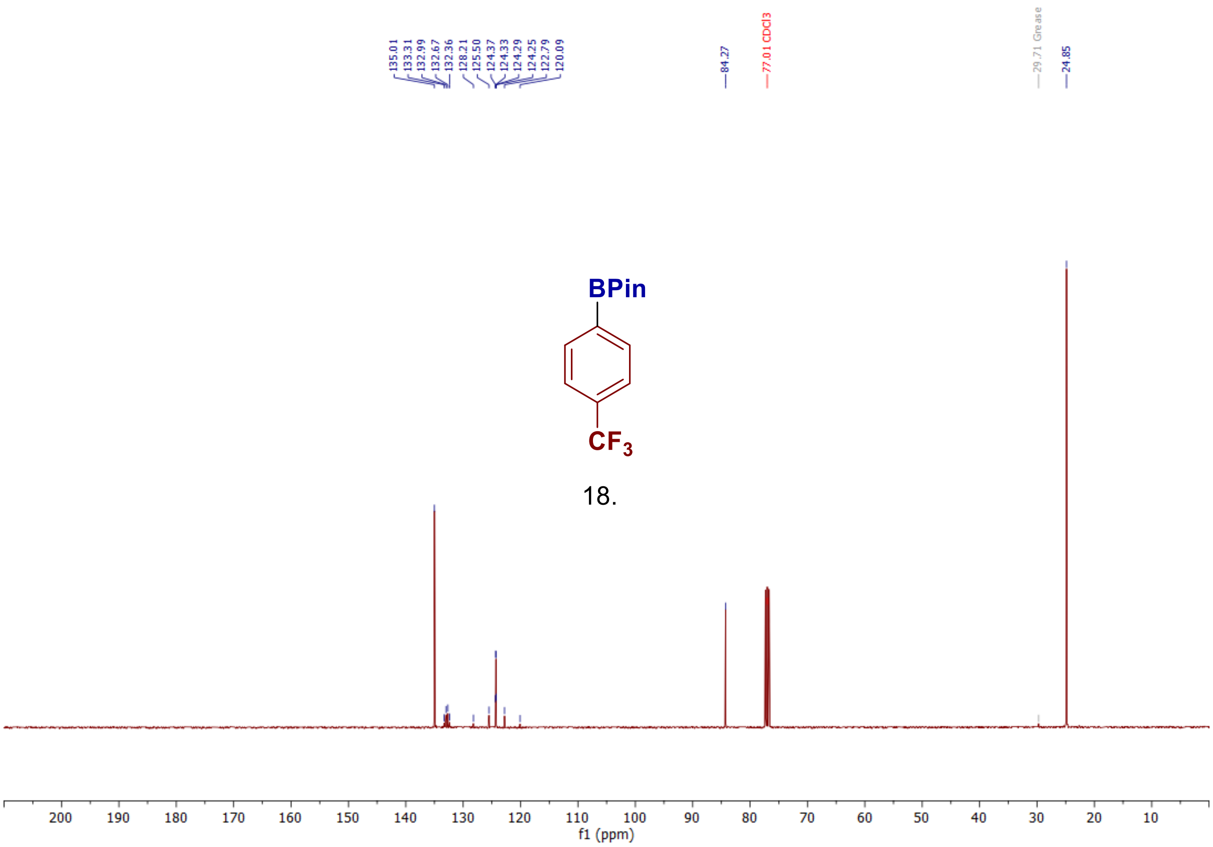

^{19}F NMR (377 MHz, $CDCl_3$)

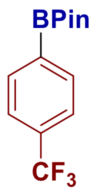

18.

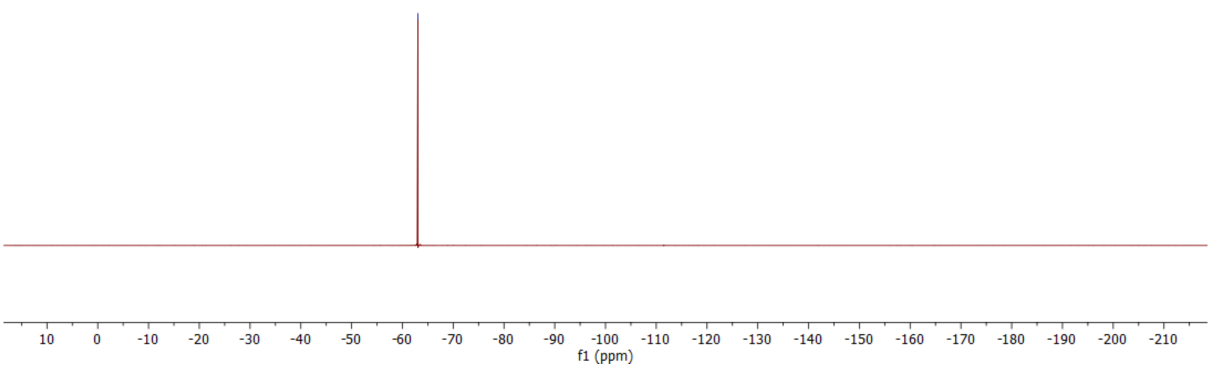

^{1}H NMR (400 MHz, $CDCl_3$)

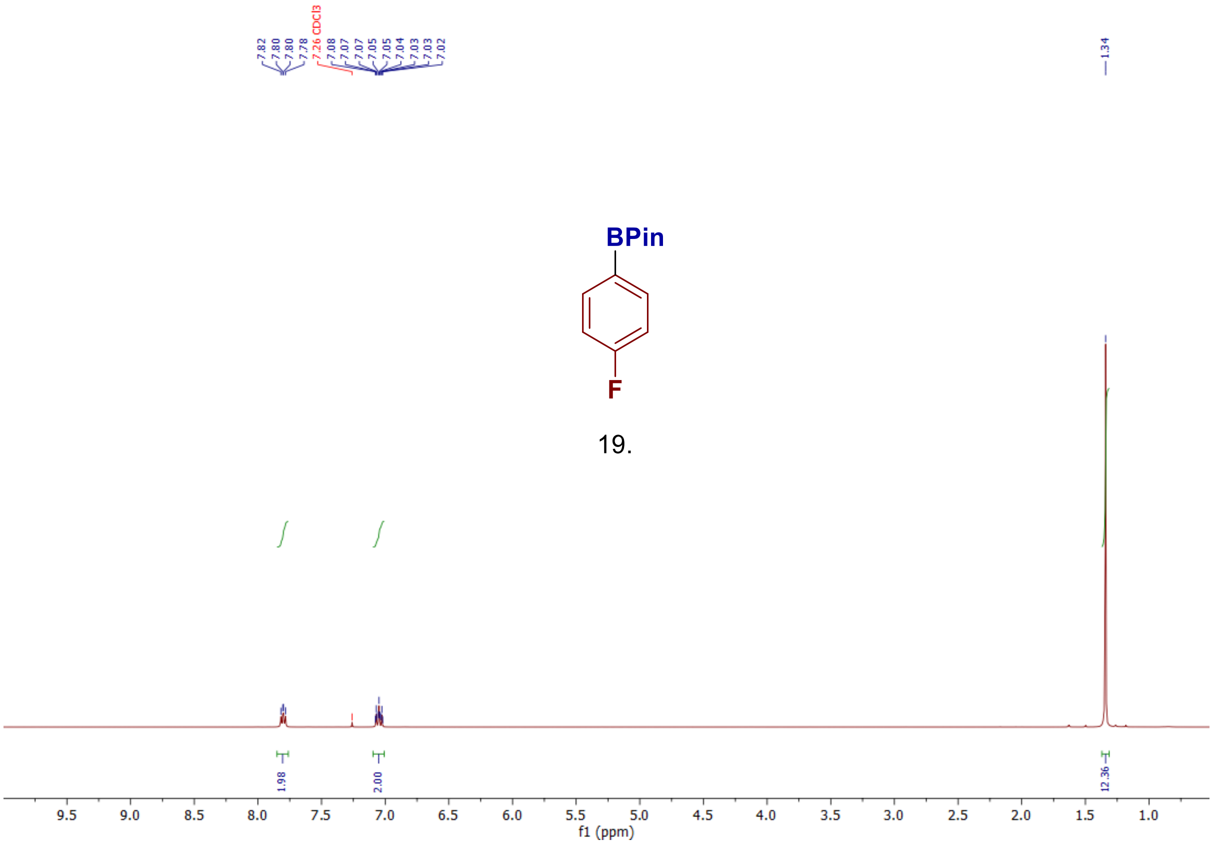

^{13}C NMR (101 MHz, $CDCl_3$)

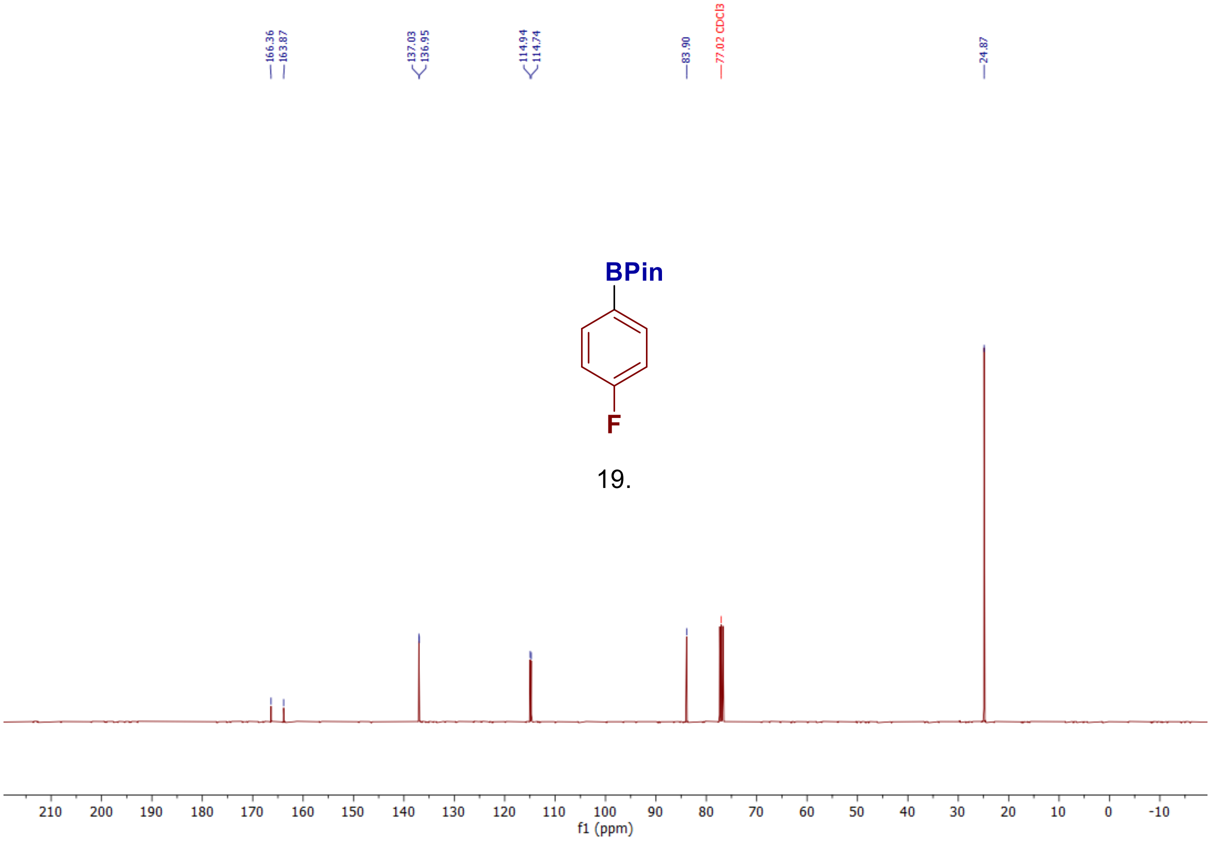

^{19}F NMR (377 MHz, $CDCl_3$)

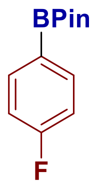

19.

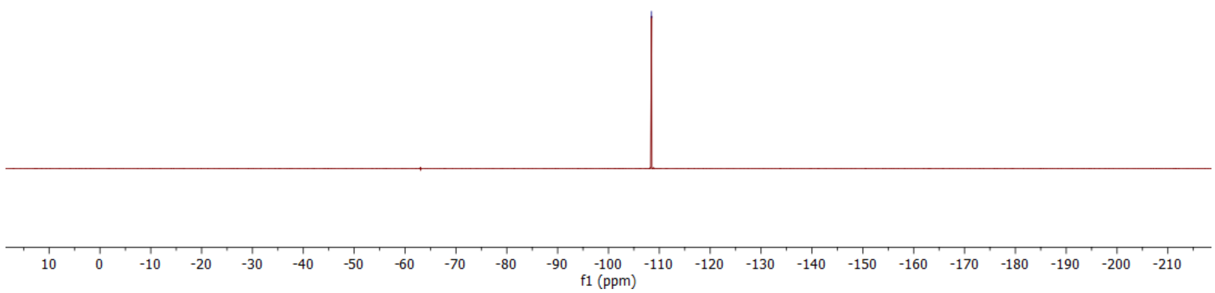

^{1}H NMR (400 MHz, $CDCl_3$)

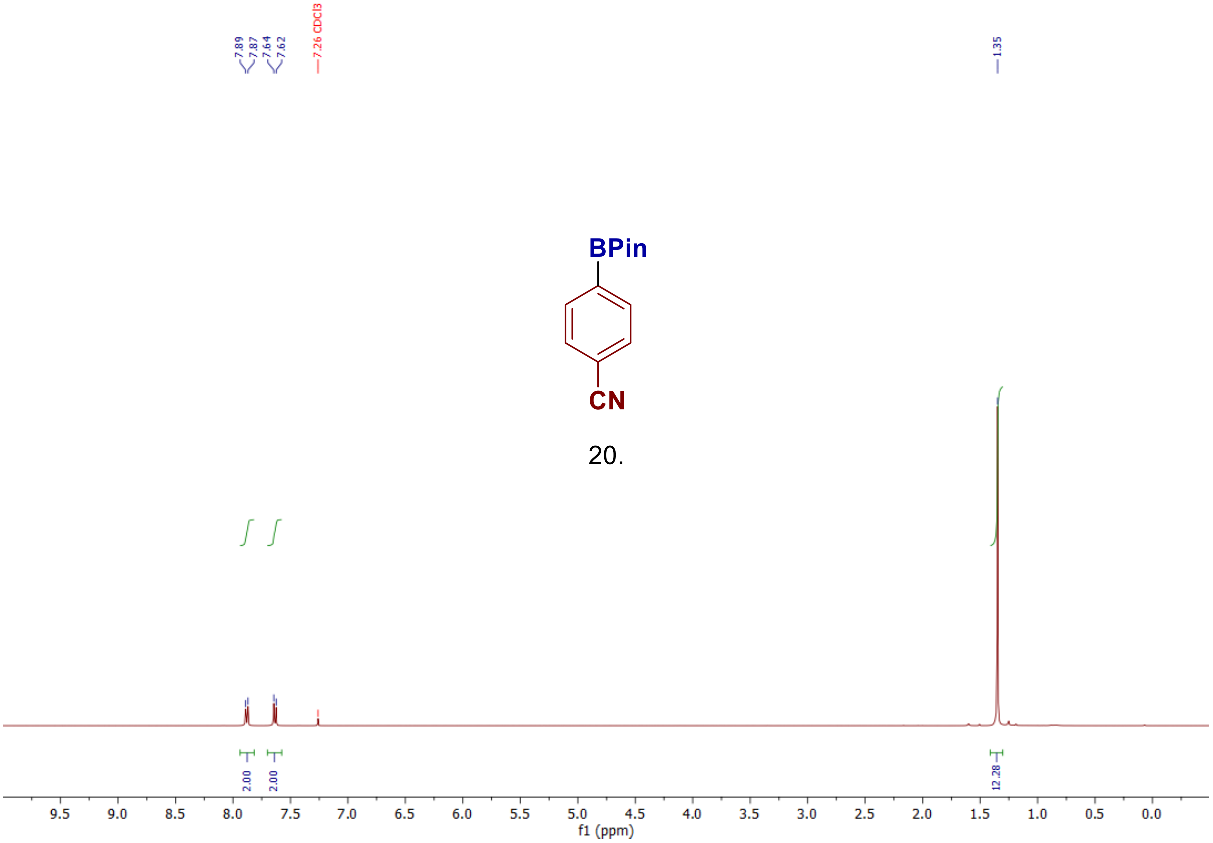

^{13}C NMR (101 MHz, $CDCl_3$)

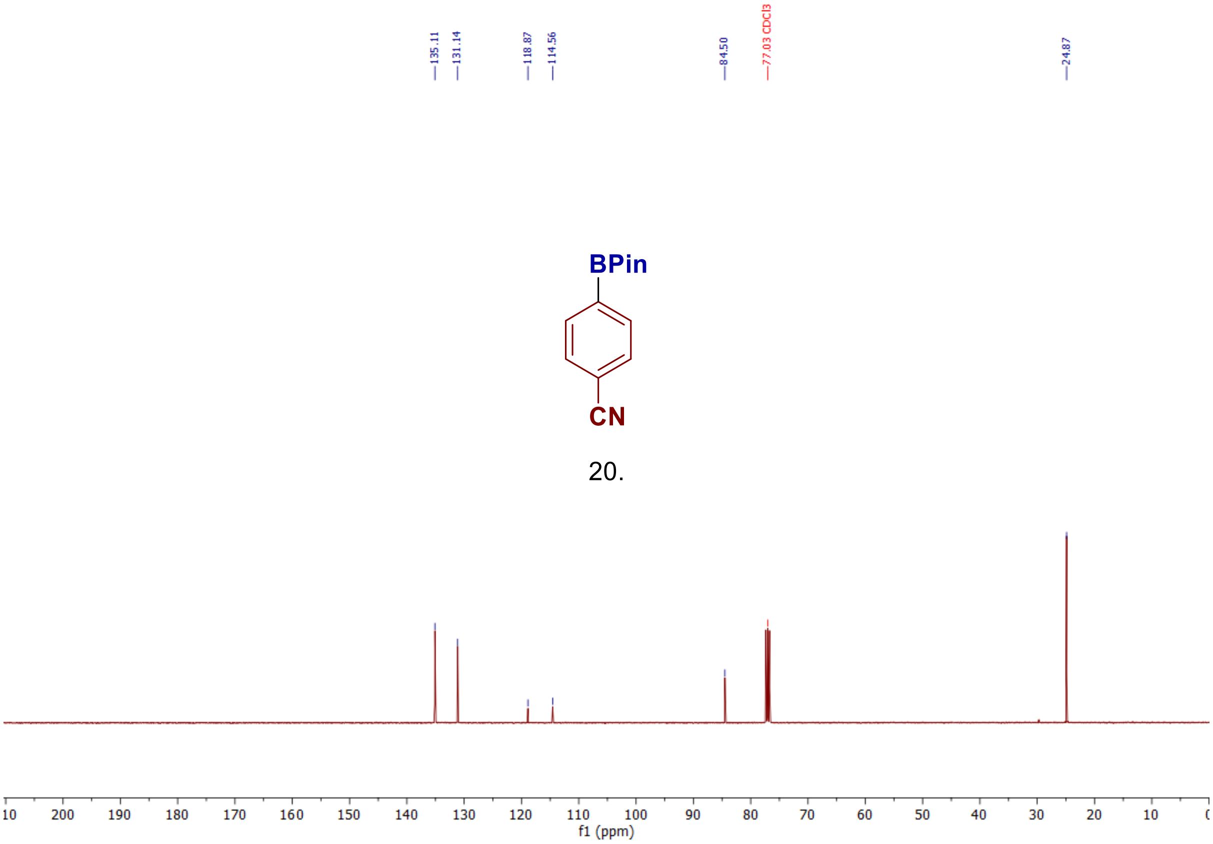

^{1}H NMR (400 MHz, $CDCl_3$)

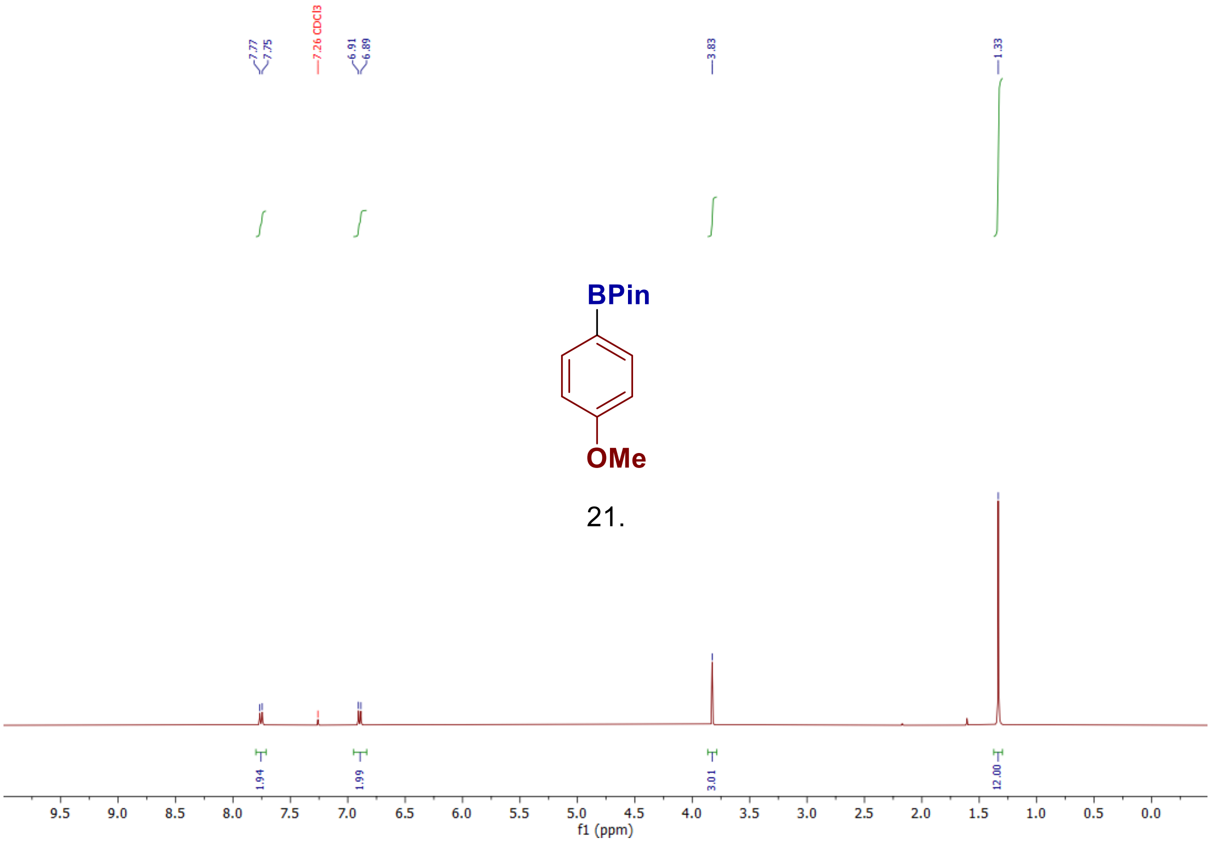

^{13}C NMR (101 MHz, $CDCl_3$)

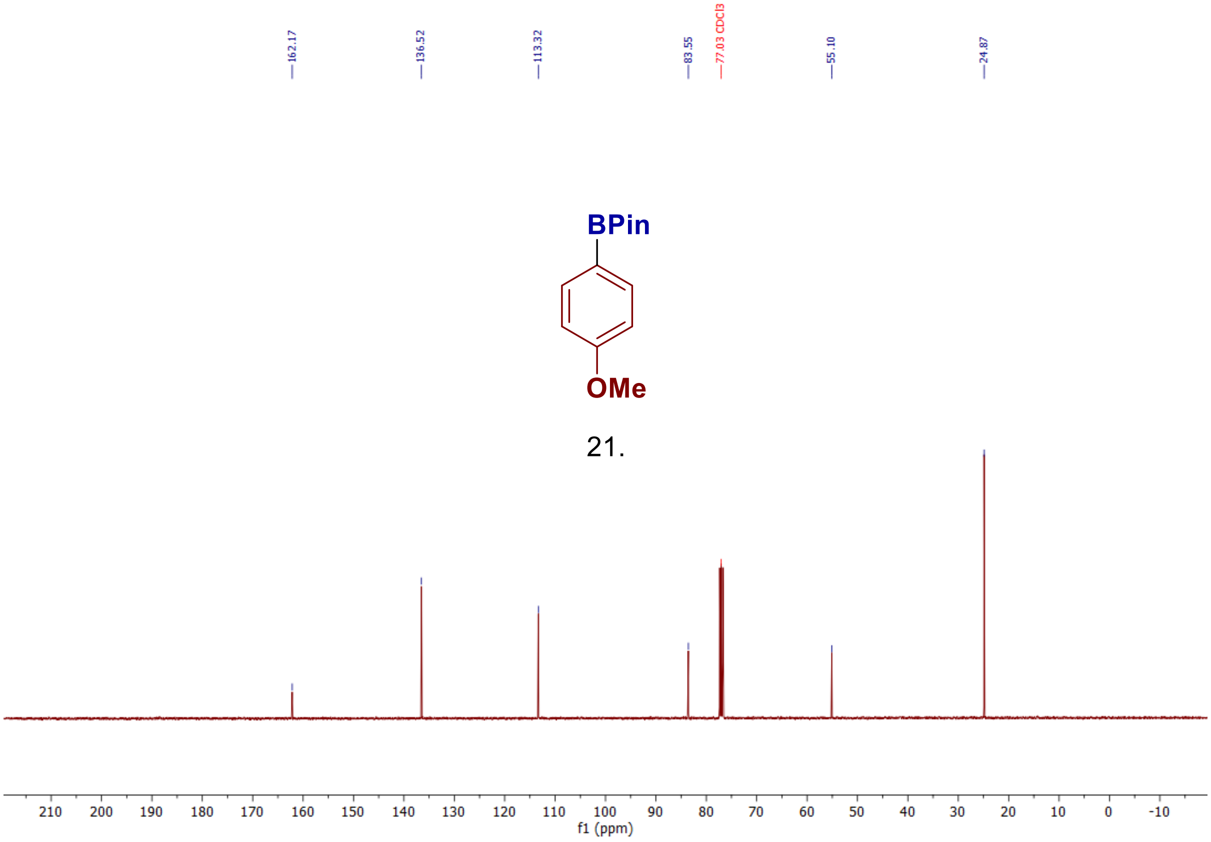

^{1}H NMR (400 MHz, $CDCl_3$)

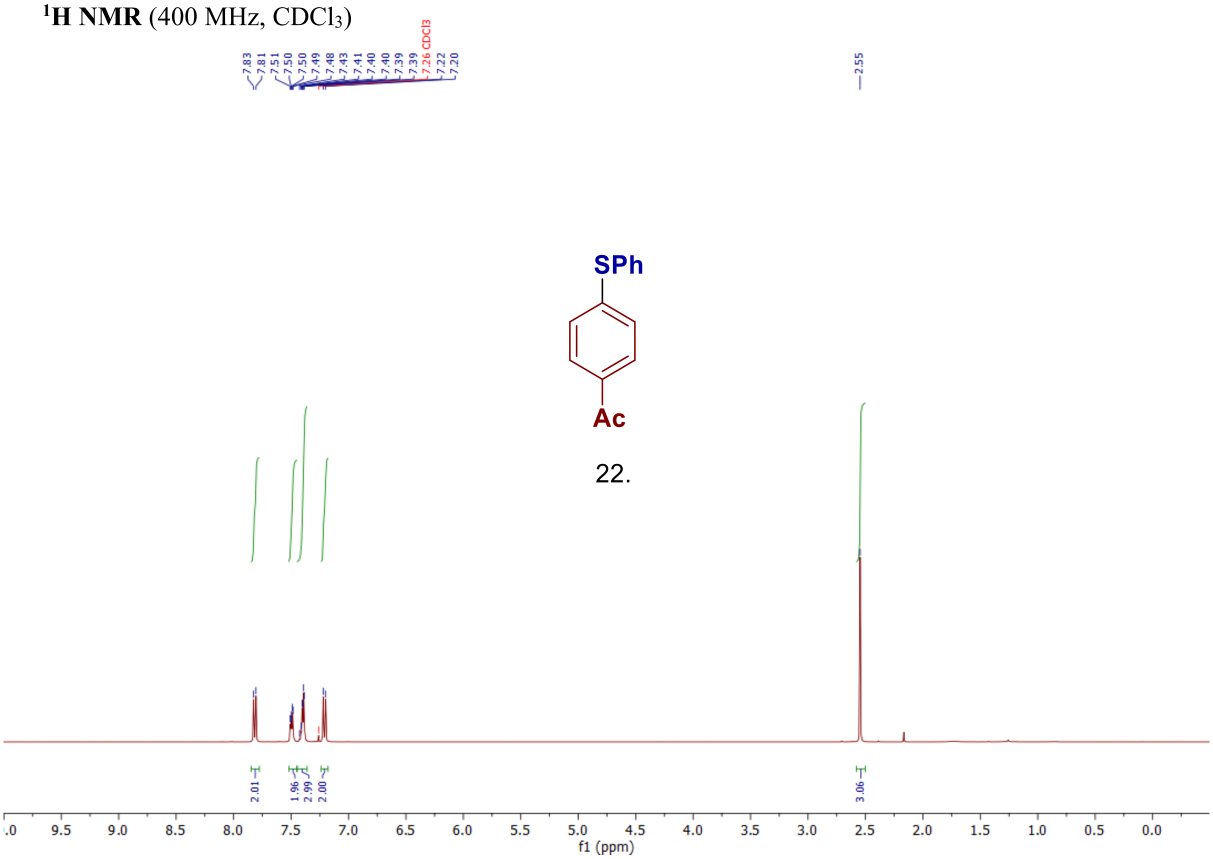

^{13}C NMR (101 MHz, $CDCl_3$)

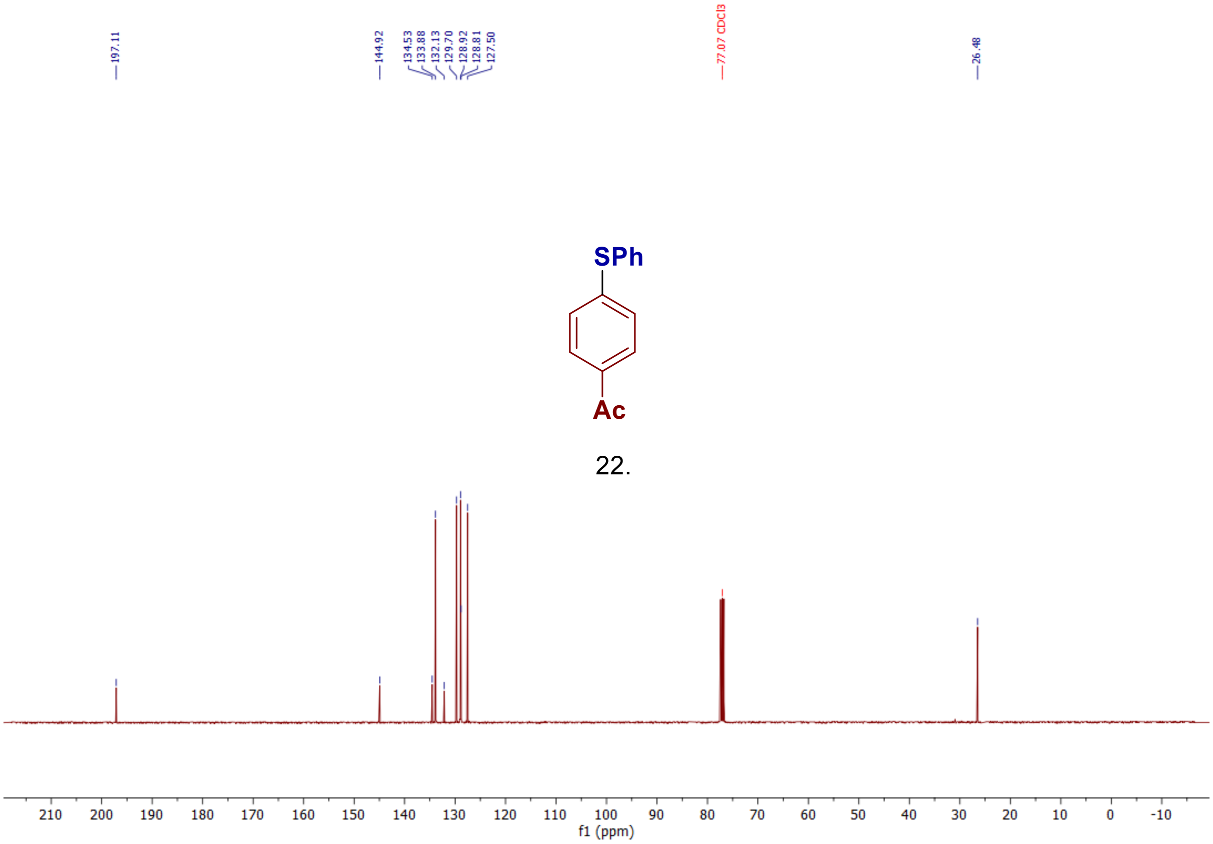

^{1}H NMR (400 MHz, $CDCl_3$)

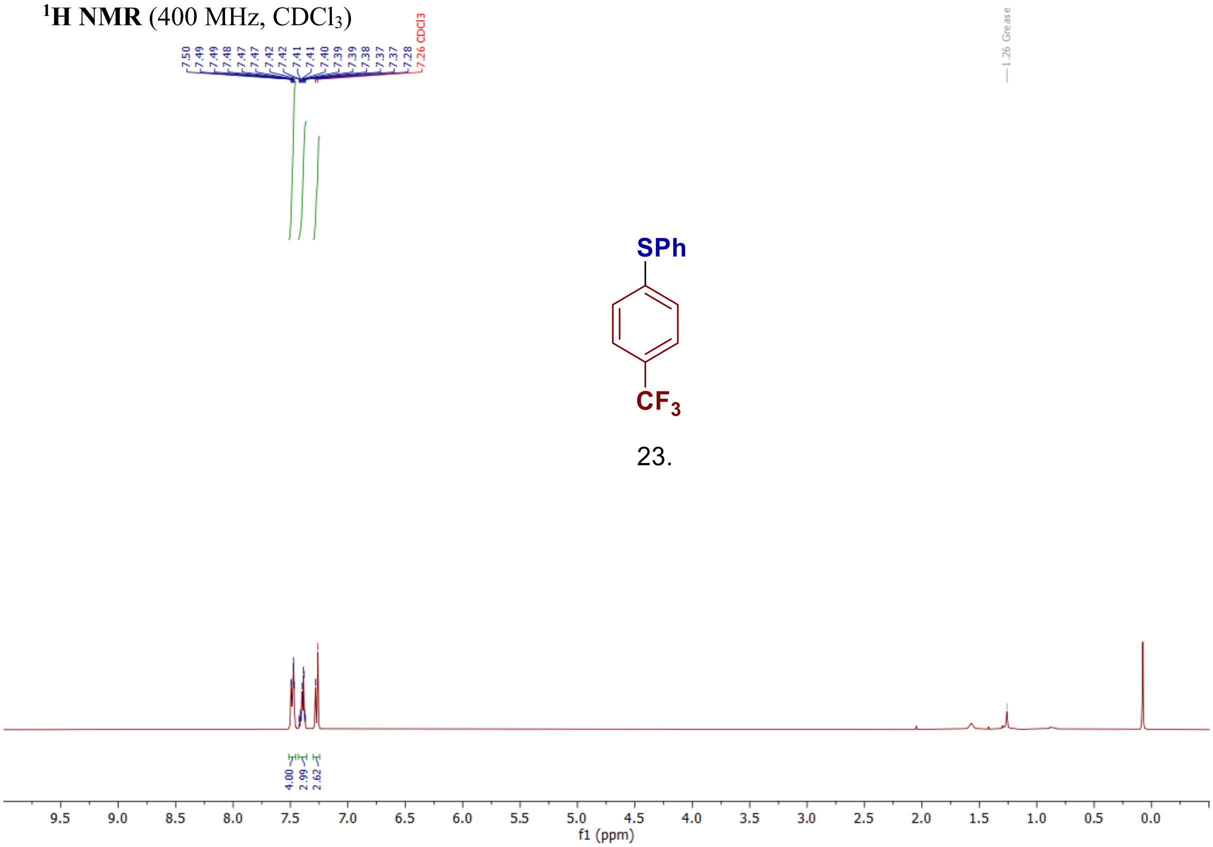

^{13}C NMR (101 MHz, $CDCl_3$)

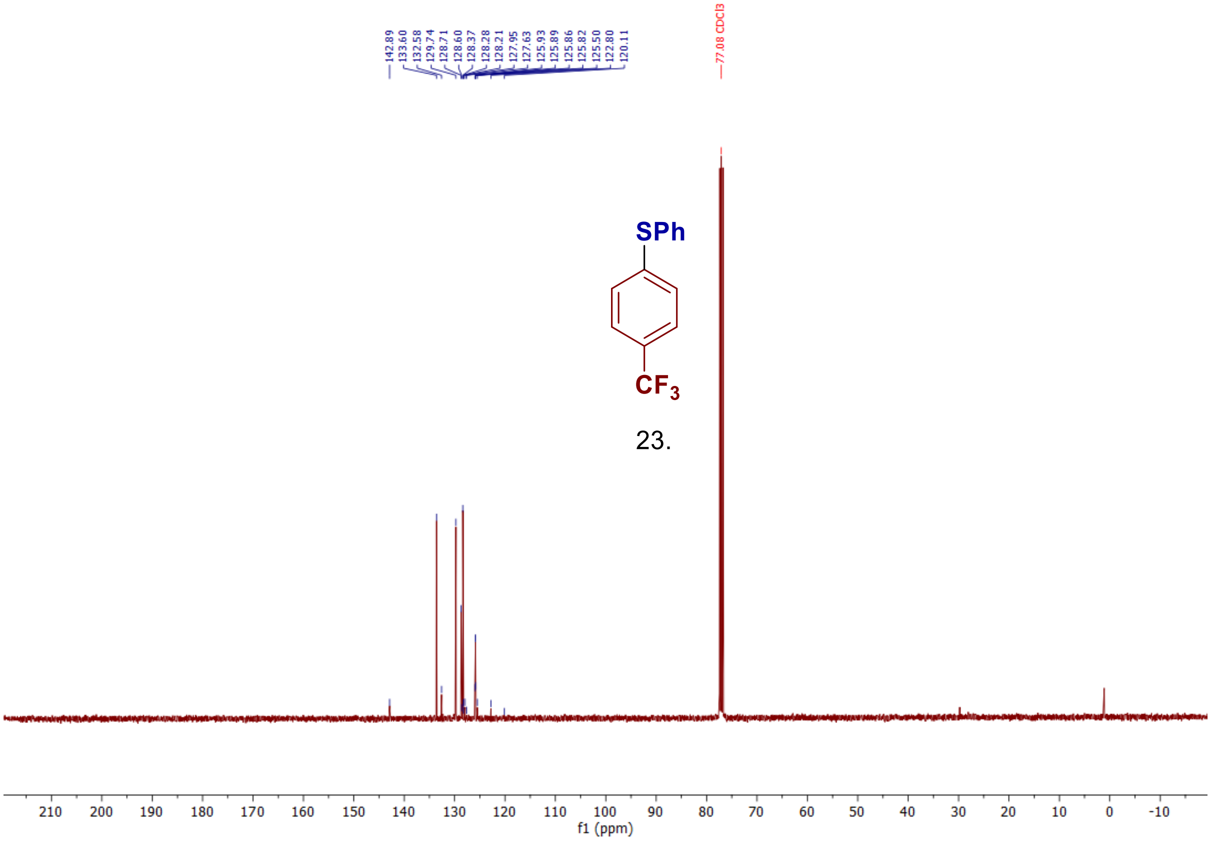

^{19}F NMR (377 MHz, $CDCl_3$)

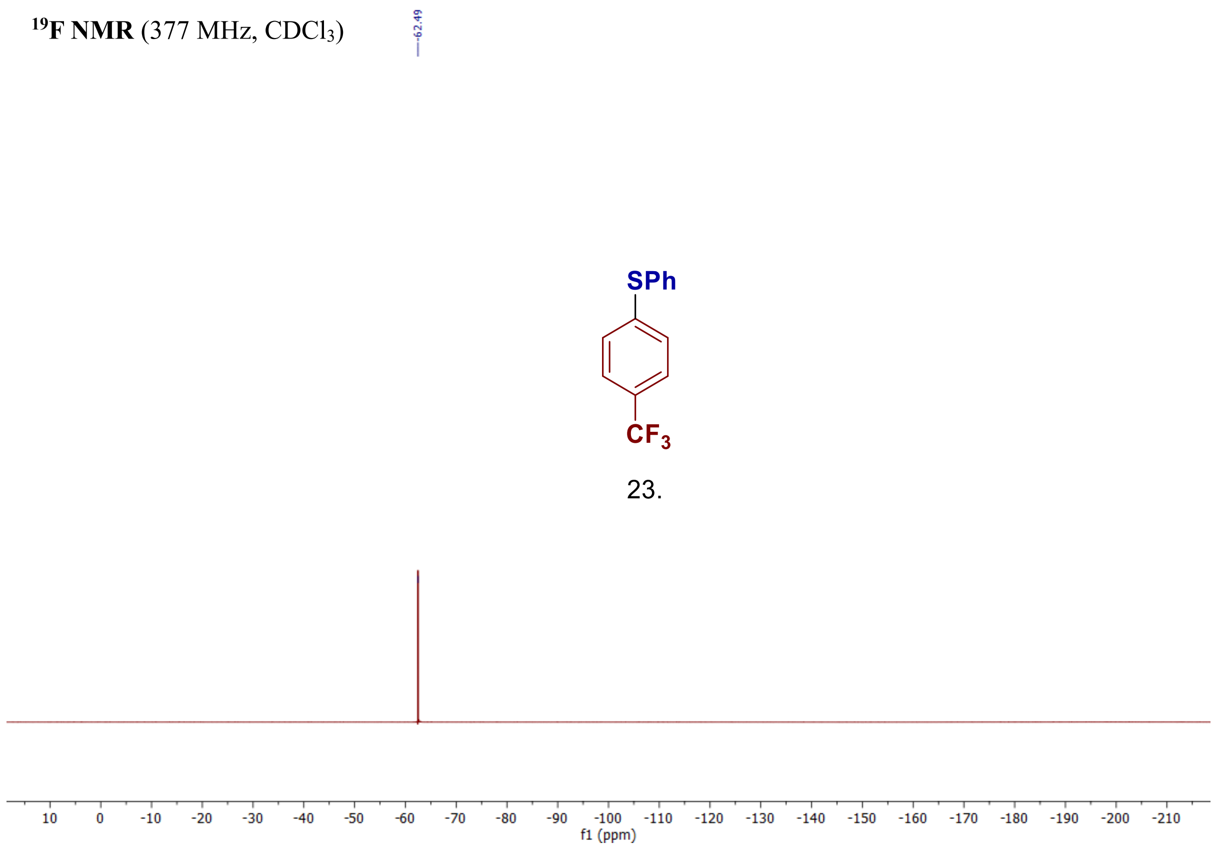

^{1}H NMR (400 MHz, $CDCl_3$)

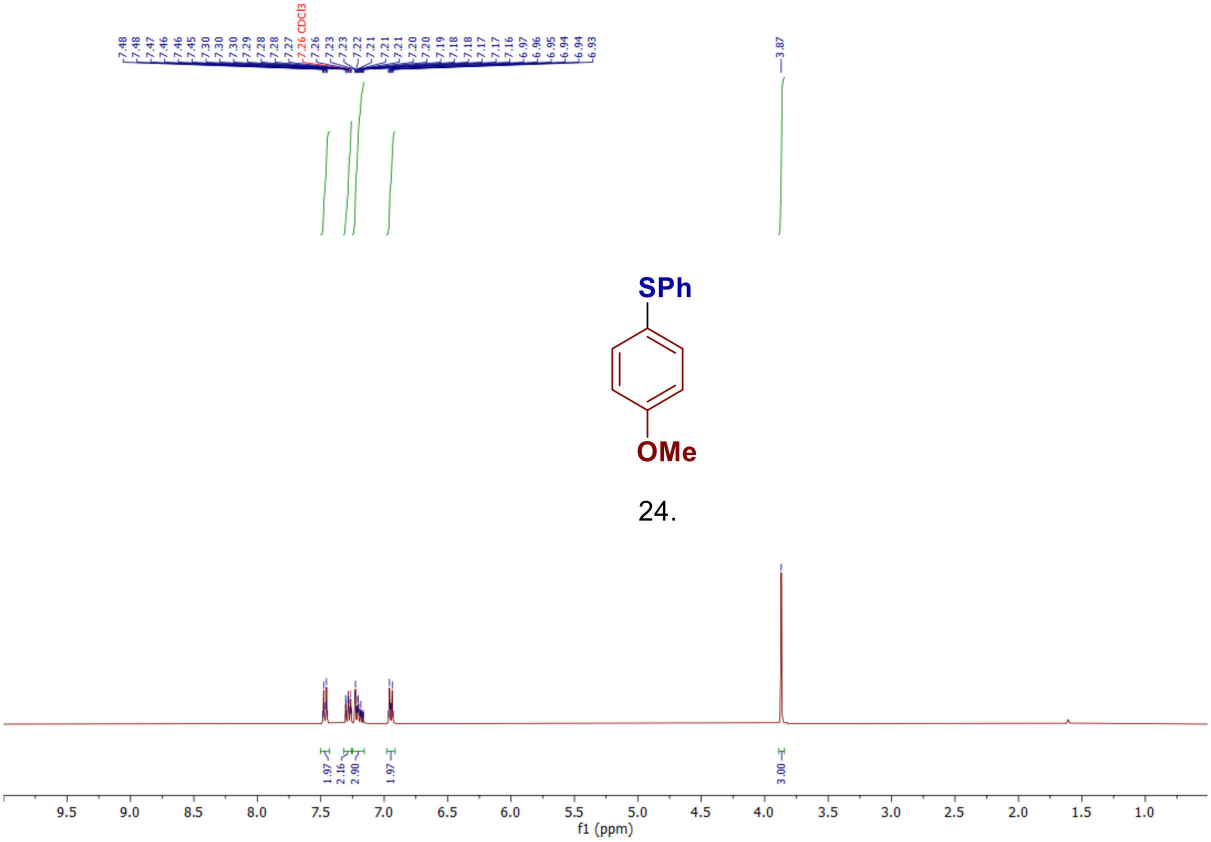

^{13}C NMR (101 MHz, $CDCl_3$)

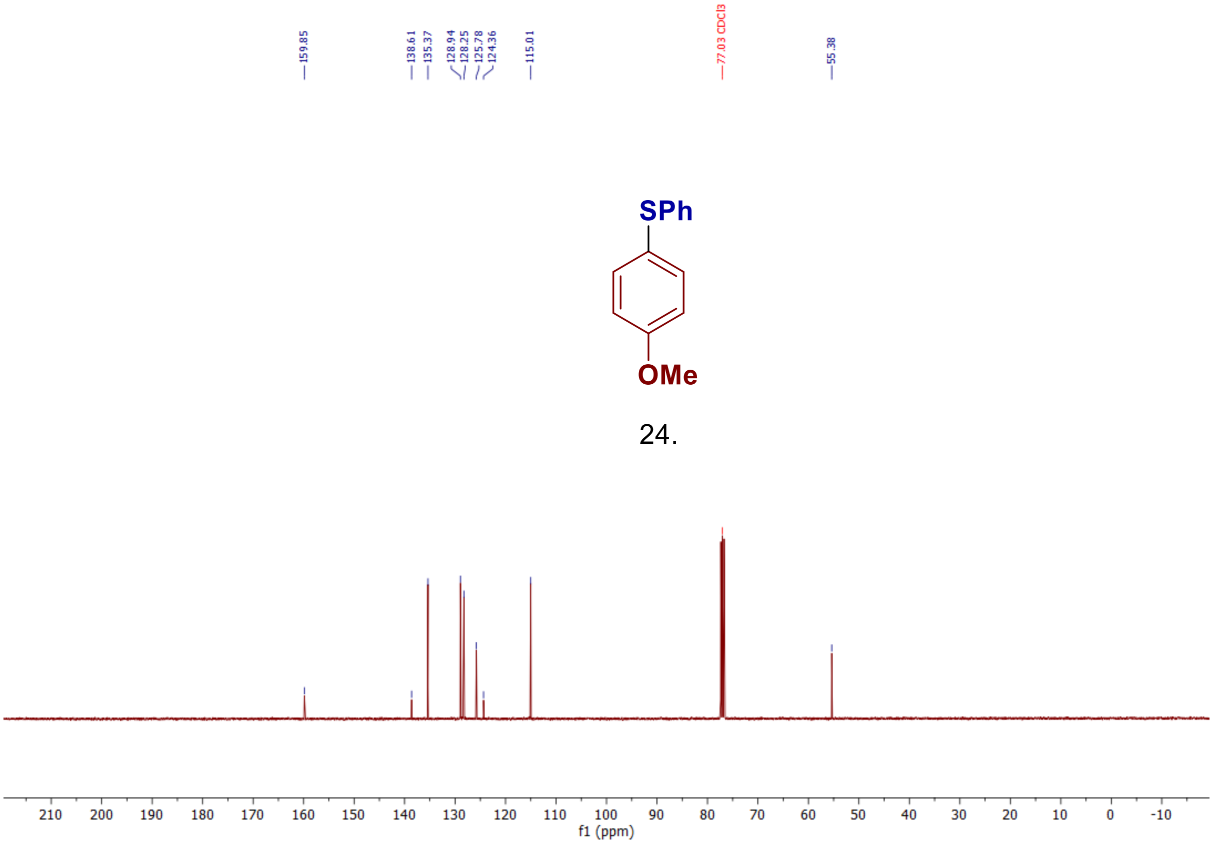

^{1}H NMR (400 MHz, $CDCl_3$)

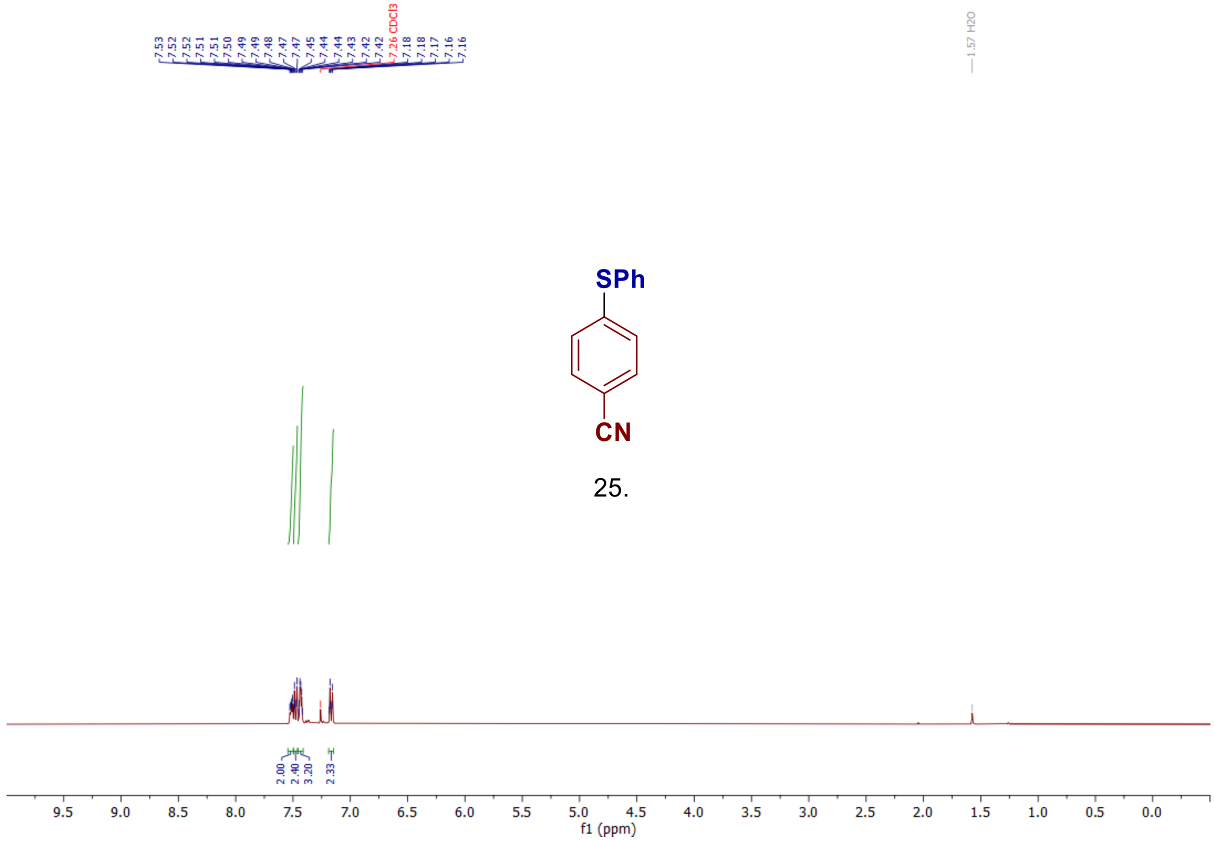

^{13}C NMR (101 MHz, $CDCl_3$)

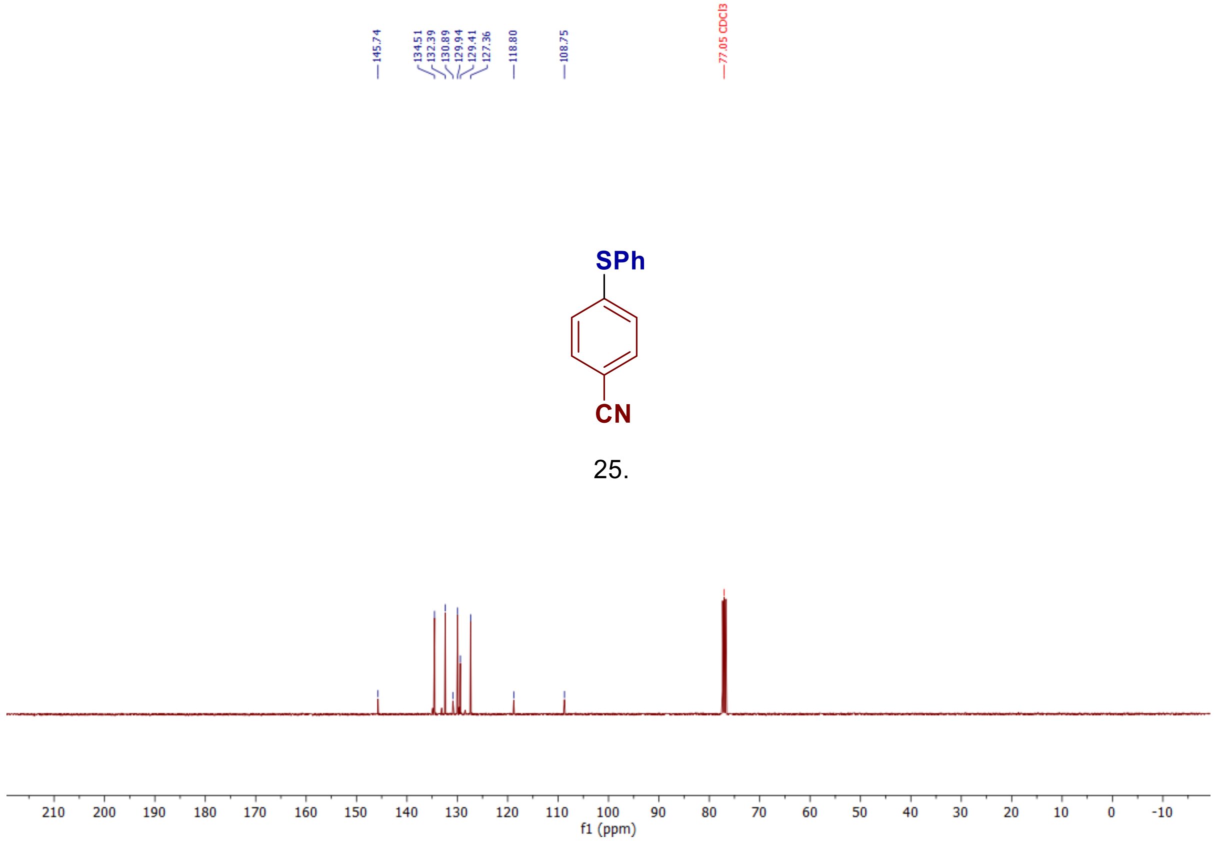

^{1}H NMR (400 MHz, $CDCl_3$)

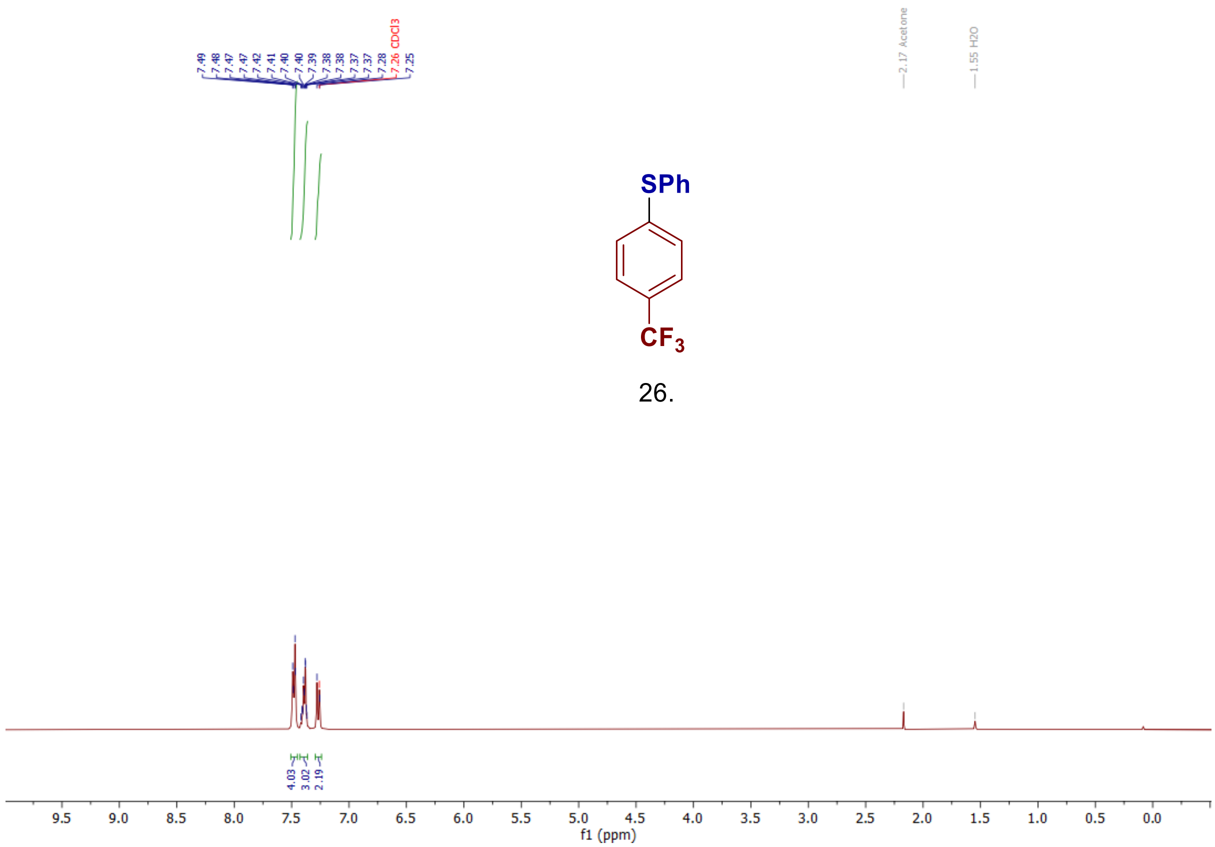

^{13}C NMR (101 MHz, $CDCl_3$)

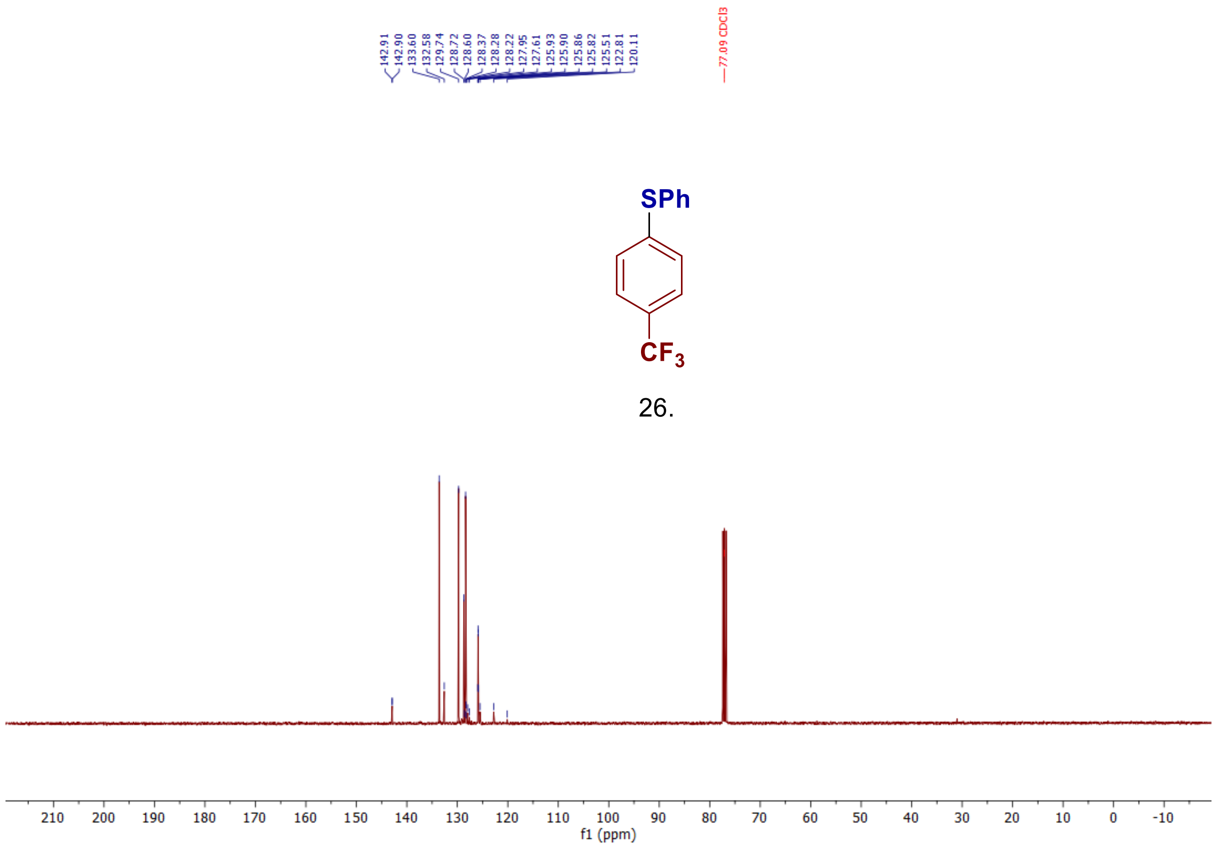

^{19}F NMR (377 MHz, $CDCl_3$)

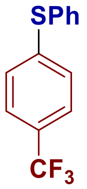

26.

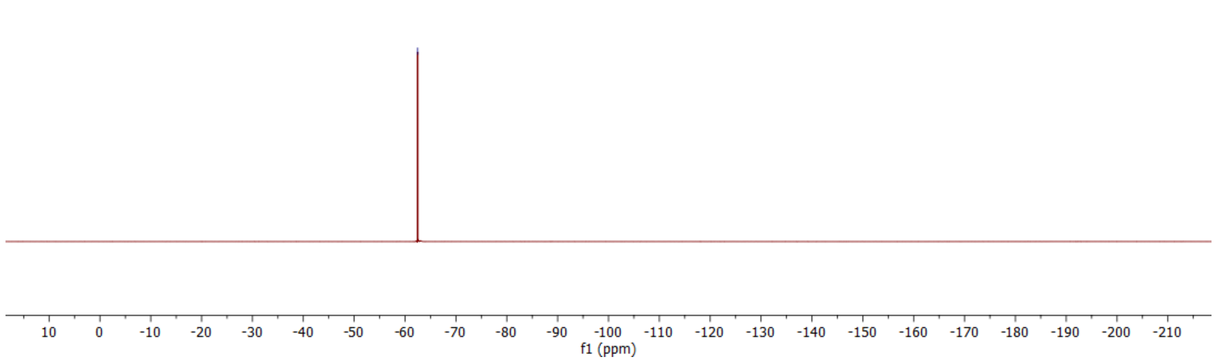

8. References

1. T. Luo, Z. Wang, Y. Chen, H. Li, M. Peng, F. Tuna, E. J. McInnes, S. J. Day, J. An, M. Schröder, *Angew. Chem. Int. Ed.* **2023**, *62*, e202306267
2. D. S. Lee, C. S. Kim, N. Iqbal, G. S. Park, K.-s. Son, E. J. Cho, *Org. Lett.* **2019**, *21*, 9950-9953.
3. F. Yu, R. Mao, M. Yu, X. Gu, Y. Wang, *J. Org. Chem.* **2019**, *84*, 9946-9956.
4. I. Ghosh, R. S. Shaikh, B. König, *Angew. Chem. Int. Ed.* **2017**, *56*, 8544-8549.
5. H. Li, X. Tang, J. H. Pang, X. Wu, E. K. Yeow, J. Wu, S. Chiba, *J. Am. Chem. Soc.* **2020**, *143*, 481-487.
6. Z. Chen, Y. Liu, C. Zeng, C. Ren, H. Li, R. V. Jagadeesh, Z. Yuan, X. Li, *Green Chem.* **2023**, *25*, 7998-8006.
7. P. Daley-Dee, J. Clarke, S. Monfette, R. B. Bedford, *Org. Lett.* **2024**, *27*, 197-201.
8. F. Bie, X. Liu, H. Cao, Y. Shi, T. Zhou, M. Szostak, C. Liu, *Org. Lett.* **2021**, *23*, 8098-8103.
9. L. Liu, J. Hou, Y. Ma, W.-H. Xu, J.-Q. Liu, D. Zhu, *Org. Lett.* **2024**, *27*, 346-351.
